# Investigating the genetic causal link between iron regulation and lung cancer risk: A 2-sample Mendelian randomization analysis

**DOI:** 10.1097/MD.0000000000045518

**Published:** 2025-10-24

**Authors:** Liqiu Yu, Zhuien Wang, Chengye Chen, Mengfan Li, Xin Sun, Yi Yang

**Affiliations:** a School of Traditional Chinese Medicine, Yantai Campus, Binzhou Medical University, Yantai, China.

**Keywords:** iron homeostasis, Mendelian randomization, NSCLC, SCLC

## Abstract

Lung cancer (LC) is among the most prevalent cancers and is the leading cause of cancer-related mortality. Smoking behavior is the primary etiological factor for LC; however, the potential causal relationship with other risk factors, such as iron status, remains unclear. Currently, there is a significant lack of research investigating the potential causal link between iron homeostasis and LC development. This study employs a 2-sample Mendelian randomization approach to explore the causal relationship between these 2 entities. Data on small cell LC (SCLC) and non-small cell LC (NSCLC) were obtained from the FinnGen R11 database, while data on iron homeostasis, encompassing 4 indicators (ferritin, serum iron, total iron binding capacity, and transferrin saturation) were sourced from the Decode Genetic Sequence Bank. The inverse variance weighted analysis demonstrated a causal genetic association between ferritin levels (β = 0.351; 95% confidence interval = 1.006–2.046; *P* = .045) and SCLC. The application of Cochran *Q* test, Rucker *Q* test, MR Egger intercept, and MR-PRESSO global tests did not reveal any evidence of heterogeneity or pleiotropy (*P* > .05). In conclusion, from a genetic perspective, elevated ferritin levels are positively correlated with an increased risk of SCLC. Furthermore, no genetic causality was observed between the other 3 indicators of iron homeostasis and either SCLC or NSCLC, nor between ferritin and NSCLC.

## 1. Introduction

Globally, lung cancer (LC) is the most prevalent type of cancer and the leading cause of cancer-related mortality.^[1]^ Lung cancer is primarily classified into 2 types based on the morphological characteristics of the cancerous tissue: small cell LC (SCLC) and non-small cell LC (NSCLC). A notable feature of SCLC is its high aggressiveness, which is characterized by early metastasis, rapid growth, and the swift emergence of drug resistance, contributing to a dismal survival rate; the approximate 5-year relative survival rate is only 6.4%. This situation underscores the urgent need for innovative therapeutic strategies.^[2]^ NSCLC accounts for 85% to 90% of all LC cases, making it the predominant subtype. This category encompasses various histological variants, including lung adenocarcinoma, squamous cell carcinoma, and large cell carcinoma.^[3]^ Tobacco smoking, particularly cigarette consumption, is the primary risk factor for the development of LC. Additionally, environmental and occupational exposures contribute to LC incidence in specific regions. Other significant risk factors include genetic predispositions and gender. LC can also present distinct characteristics in certain patient populations, such as women, individuals infected with HIV, and never-smokers.^[4]^ Currently, the primary treatment modalities for LC include surgery, along with adjuvant chemotherapy and radiotherapy.^[5]^ Iron, a pivotal nutrient in the human body, is intricately involved in numerous vital biological mechanisms. Its indispensable functions encompass facilitating mitochondrial respiration, maintaining redox homeostasis, catalyzing ATP synthesis, enhancing immune competence, facilitating DNA synthesis, and modulating hormone synthesis, thereby underpinning various essential physiological processes.^[6,7]^ To sustain adequate iron levels, cells coordinate multiple genes to maintain iron homeostasis, ensuring their collective function in regulating intracellular iron metabolism.^[8–10]^ This process is known as iron homeostasis. In recent years, the introduction of the concept of iron homeostasis has led to a significant increase in research in this area. It is widely recognized that maintaining iron homeostasis is crucial for the development of various diseases. Iron homeostasis not only regulates inflammatory responses but also plays a significant role in the immune response during host infections.^[11,12]^ Research indicates that under specific conditions of iron overload, iron enhances the recruitment of neutrophils and inflammatory responses by upregulating interleukin-1β.^[13]^ Rochette et al demonstrated that disrupted iron homeostasis in COVID-19 patients triggers oxidative stress and inflammatory reactions.^[14]^ Deferoxamine and deferasirox, both iron chelators approved by the U.S. Food and Drug Administration, have demonstrated efficacy against various cancers, including leukemia, neuroblastoma, colorectal cancer, and breast cancer.^[15–18]^ The primary instigator of LC is habitual smoking; however, the potential causal association of other risk factors has garnered extensive attention in recent years. Relevant factors include iron status, which has been the subject of considerable research regarding its interplay with cancer. Despite this, there has been a notable lack of nuanced investigation into the specific causal link between iron status and LC. Some scholars have posited that diets high in iron may elevate the risk of developing LC, thus warranting further scrutiny.^[19–23]^ Costa et al have indicated that in LC, iron-containing tumor-associated macrophages increase the levels of reactive oxygen species and secrete pro-inflammatory cytokines, such as TNF-α and IL-6, which contribute to tumor cell death.^[24]^ However, the precise pathological mechanisms underlying the role of iron homeostasis in the development of LC remain elusive. Therefore, robust causal inference methods are essential for analyzing the causal relationship between iron homeostasis and LC, thereby establishing a theoretical foundation for prospective studies and clinical applications.

Mendelian randomization (MR) analysis is an effective approach for analyzing biological information. MR represents a technique that harnesses observed genetic variations in functionally characterized genes as a means to interrogate the causal influence of a modifiable exposure factor on disease outcomes within the context of observational studies. It depends heavily on Mendel laws. This implies that during meiosis, when gametes are formed, alleles are randomly passed on to offspring from their parents. Consequently, the associations between genes and outcomes are not affected by typical confounding factors like postnatal environment, socioeconomic status, and behavioral habits. The causative relationships suggested by this approach are plausible, establishing the theoretical basis for MR. By utilizing randomly assigned genetic instrumental variables (IVs), specifically single nucleotide polymorphisms (SNPs), and accounting for confounding factors, this technique effectively assessing the causal link between exposure and outcomes. In this research, our primary aim is to elucidate the genetic interplay between iron regulation and the etiology of LC, employing a 2-sample MR framework to analyze aggregated data obtained from genome-wide association studies (GWAS).^[25–27]^ This approach endeavors to provide clarity on the underlying genetic connections.

## 2. Methods

### 2.1. Research design

**Figure 1.**
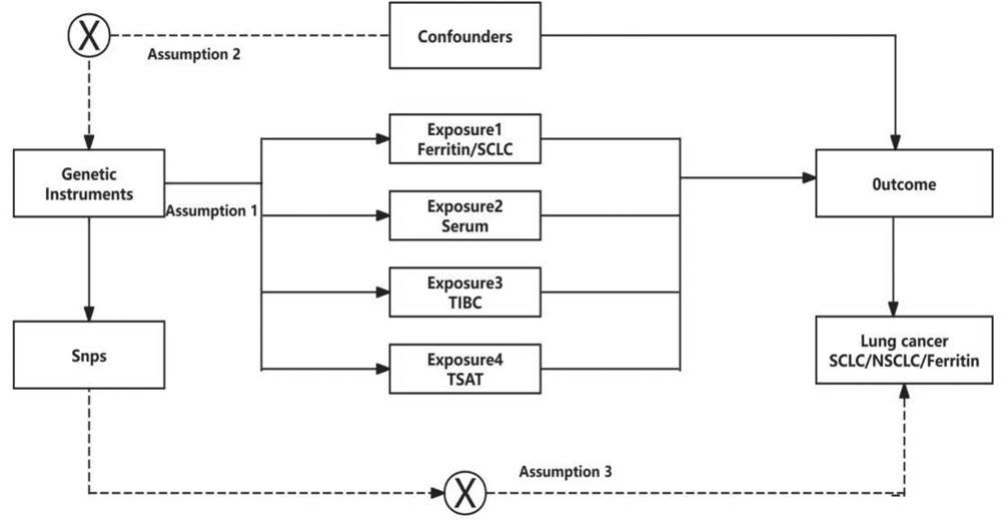
Schematic diagram of a 2-sample Mendelian randomization (MR) study design. The 3 fundamental assumptions of Mendelian randomization (MR): (1) relevance: the SNPs, serving as instrumental variables, are strongly associated with the exposure. (2) Independence: the SNPs are independent of confounding factors. (3) Exclusion restriction: the SNPs influence the outcome exclusively through the exposure, rather than via any other pathways. NSCLC = non-small cell lung cancer, SCLC = small cell lung cancer, SNPs = single nucleotide polymorphisms, TIBC = total iron binding capacity, TSAT = transferrin saturation.

In the current investigation, we utilized SNPs derived from GWAS datasets as IVs to elucidate the genetic underpinnings linking exposure to outcome. This endeavor strictly adheres to the 3 cardinal premises of MR research: firstly, ensuring that all selected IVs exhibit robust associations with the exposure variable (indicated by *P* < 5 × 10⁻^8^ and an *F*-statistic exceeding10); secondly, affirming their independence from confounding factors and the absence of direct correlations with either the exposure or the outcome, thereby ensuring neutrality; and thirdly, establishing that these variables mediate their influence on outcome events solely through the exposure, without interference from alternative biological pathways.^[28,29]^ A diagrammatic representation of this study’s methodology is presented in Figure 1.

### 2.2. Data sources

The LC-associated information was extracted from the summary statistics available in the FinnGen R11 repository (https://www.finngen.fi/en). After excluding non-Finnish ancestry samples through principal component analysis and conducting a rigorous screening for missing data, heterozygosity, sex concordance, and SNP quality, we ultimately retained high-quality samples and SNPs. This process resulted in a cohort consisting of 855 Finnish SCLC cases and 345,118 controls, as well as 2064 Finnish NSCLC cases and 345,118 controls.^[30]^ (The publicly available summary data do not provide further histological subtyping of NSCLC into adenocarcinoma, squamous cell carcinoma, or large cell carcinoma). Data on iron homeostasis were obtained from the deCODE summary data portal (https://www.decode.com/summarydata/), 4 biomarkers were included: ferritin (N = 246,139), serum iron (N = 163,511), total iron binding capacity (TIBC) (N = 135,430), and transferrin saturation (TSAT) (N = 131,471).^[31]^ All aggregated data used in this research were obtained from public databases and are freely downloadable (Table S1, Supplemental Digital Content, https://links.lww.com/MD/Q480). All individuals recruited for this study hailed from a European genetic background, ensuring a homogeneous ancestral lineage among the participants.

### 2.3. Selection of IVs

We implemented a series of quality control criteria to select appropriate genetic IVs. In the initial genome-wide association analysis, a threshold of *P* < 5 × 10⁻^8^ was first applied for screening; however, the number of obtained SNPs was limited. To acquire more robust IVs, we relaxed the genome-wide significance threshold to *P* < 1 × 10⁻^5^.^[32,33]^ To ensure the independence of the IVs, all IVs underwent linkage disequilibrium clustering (*R*² < 0.001 with a cluster window size of 10,000 kb) to minimize the influence of correlated SNPs. The samples from the European 1000 Genomes Project served as the reference panel for linkage disequilibrium calculations.^[34]^ In this study, we calculated the *F*-statistic using the formula: *F* = *R*² (N - *K* - 1)/*K*(1 - *R*²), where *R*² denotes the variance of IVs in the exposure variable. The parameter *R*² is defined as 2 × MAF × (1 - MAF) × β², with N representing the valid sample size from GWAS and *K* indicating the total number of variables. This metric quantifies both the reliability of each IV and the percentage of variance explained by these variables.^[35]^ *F* statistics > 10 is used as a threshold to prevent weak IV bias.^[36,37]^ We further screened for SNPs associated with the results and excluded invalid SNPs with *P*-values less than 1 × 10⁻^5^.^[38]^ To ensure the specificity of the identified SNPs, we conducted a comprehensive screening using LDLink (https://ldlink.nci.nih.gov/) to exclude SNPs associated with outcome-related factors, such as pulmonary function, chronic obstructive pulmonary disease, and high cholesterol (rs6704070, rs2473236, and rs1214355). This validation confirms that these SNPs do not exhibit pleiotropic effects on other phenotypes at genome-wide significance, as such effects could potentially distort the conclusions of the study.^[39]^ (Tables S2–S9, Supplemental Digital Content, https://links.lww.com/MD/Q480).

### 2.4. MR analysis

Utilizing the “TwoSampleMR” package within R software (version 4.3.3; R Core Team, Auckland, New Zealand), we conducted a 2-sample MR analysis aimed at elucidating the relationship linking iron homeostasis to LC risk. As the cornerstone approach, we leveraged the inverse variance weighted (IVW) method with random effects, which was complemented by the MR Egger regression, weighted median estimator, simple modal analysis, and weighted modal methodology. Notably, the IVW technique, through meta-analytical integration of the Wald statistics of IVs, furnishes more stabilized, precise, and trustworthy assessments of the causal linkage between the exposure variable and the outcome.^[40,41]^ Hence, the primary outcomes of our MR analysis are centered on the IVW analysis results. Furthermore, the Egger regression methodology functions as an auxiliary tool, leveraging its intercept values^[42,43]^ to authenticate and evaluate its multiple-source validity. The weighted median analysis approach enables the derivation of the median from the distribution function by ordering the weights attributed to each SNP effect value, yielding a relatively sturdy estimation, particularly when valid IVs contribute at least half of the overall information. In comparison to the IVW technique,^[44]^ both the simple mode and weighted mode methodologies exhibit diminished evaluative capabilities, hence their frequent utilization as supportive analytical strategies. To account for multiple testing, we applied a false discovery rate (FDR) correction, establishing a threshold of FDR < 0.05 for determining statistical significance. Associations with *P*-values <.05 and FDR <0.2 were classified as exploratory results.^[45,46]^

### 2.5. Sensitivity analysis

To delve deeper into the potential causal relationship between iron homeostasis and LC, we performed an exhaustive sensitivity analysis, with a keen focus on horizontal pleiotropic effects, the evaluation of heterogeneity, and the application of leave-one-out validation methodologies, as previously documented in references.^[27,47–49]^ The absence of genetic pleiotropy was inferred from the Egger intercept analysis, specifically when the regression intercept deviated from 0 with statistical significance (*P* < .05). Additionally, the MR-PRESSO global test served as a corroborative measure for horizontal pleiotropy. Heterogeneity among individual SNPs was quantified using the Cochran *Q* test and Rucker *Q* test *P*-value; a *P*-value exceeding .05 implied homogeneity among the selected IVs, negating the need to adjust for heterogeneity in the study’s conclusions. The leave-one-out approach was implemented by systematically excluding each SNP and monitoring the subsequent alterations in the outcomes of the remaining SNPs. A lack of significant alteration in the overall confidence intervals (CIs) post-exclusion signified reliable results, whereas any notable changes indicated inconsistency in the findings.

## 3. Results

In accordance with the screening criteria for IVs in this study, a coordinated analysis was conducted involving 351 SNPs associated with iron homeostasis markers and 3459,733 SNPs linked to SCLC, as well as 350 SNPs related to iron homeostasis markers and 347,182 SNPs associated with NSCLC. Among these SNPs, 165 related to SCLC were found to be associated with ferritin, 72 with serum iron, 62 with TIBC, and 62 with TSAT. For NSCLC, 167 SNPs were associated with ferritin, 72 with serum iron, 63 with TIBC, and 58 with TSAT. Notably, the *F*-statistic values for all SNPs exceeded 10, effectively mitigating the risk of weak instrument bias.

### 3.1. Results of MR analysis

**Figure 2.**
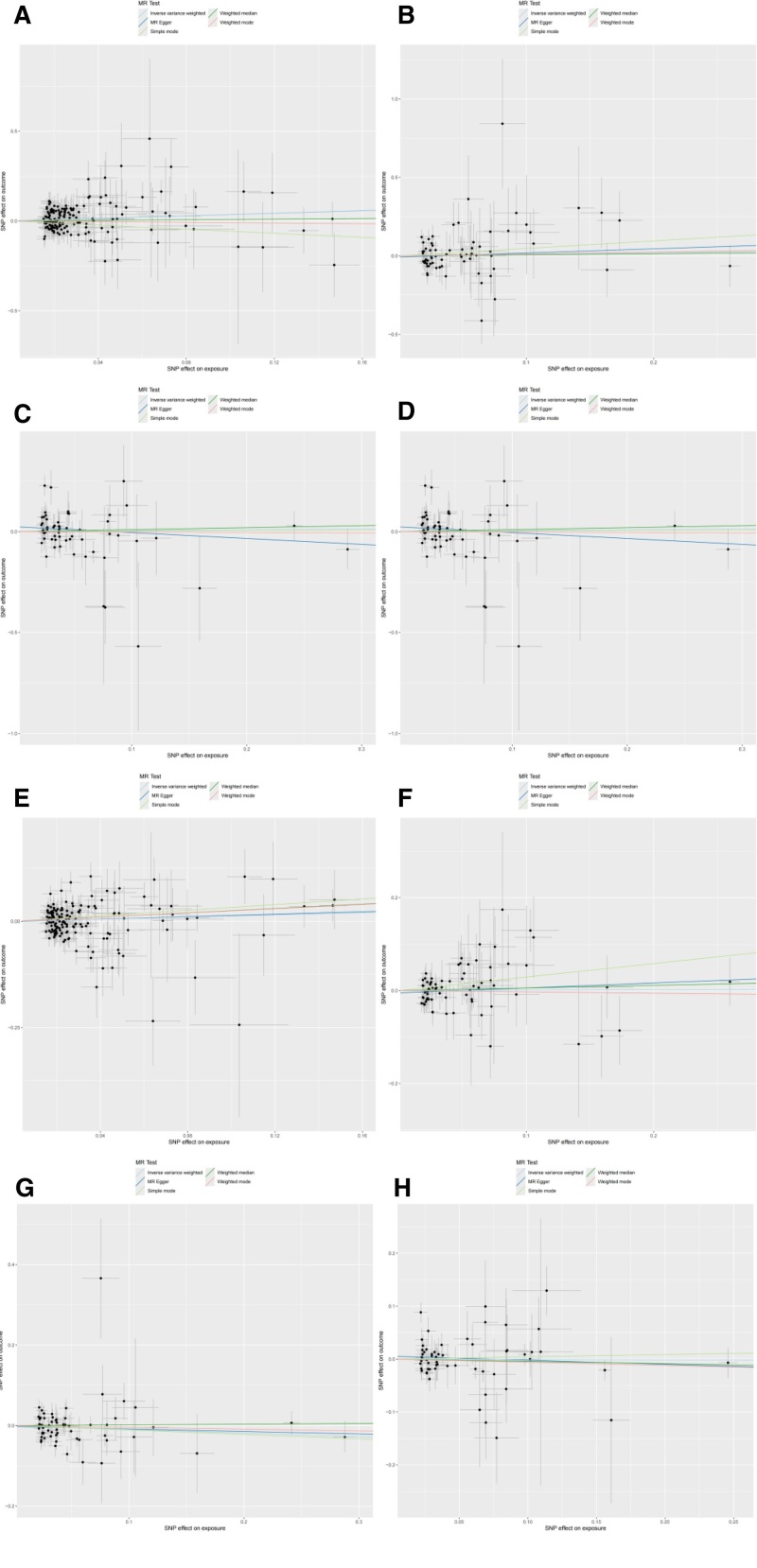
(A) Scatter plot of the causal effect of ferritin on SCLC. (B) Scatter plot of the causal effect of serum on SCLC. (C) Scatter plot of the causal effect of TIBC on SCLC. (D) Scatter plot of the causal effect of TSAT on SCLC. (E) Scatter plot of the causal effect of ferritin on NSCLC. (F) Scatter plot of the causal effect of serum on NSCLC. (G) Scatter plot of the causal effect of TIBC on NSCLC. (H) Scatter plot of the causal effect of TSAT on NSCLC. NSCLC = non-small cell lung cancer, SCLC = small cell lung cancer, TIBC = total iron binding capacity, TSAT = transferrin saturation.

**Figure 3.**
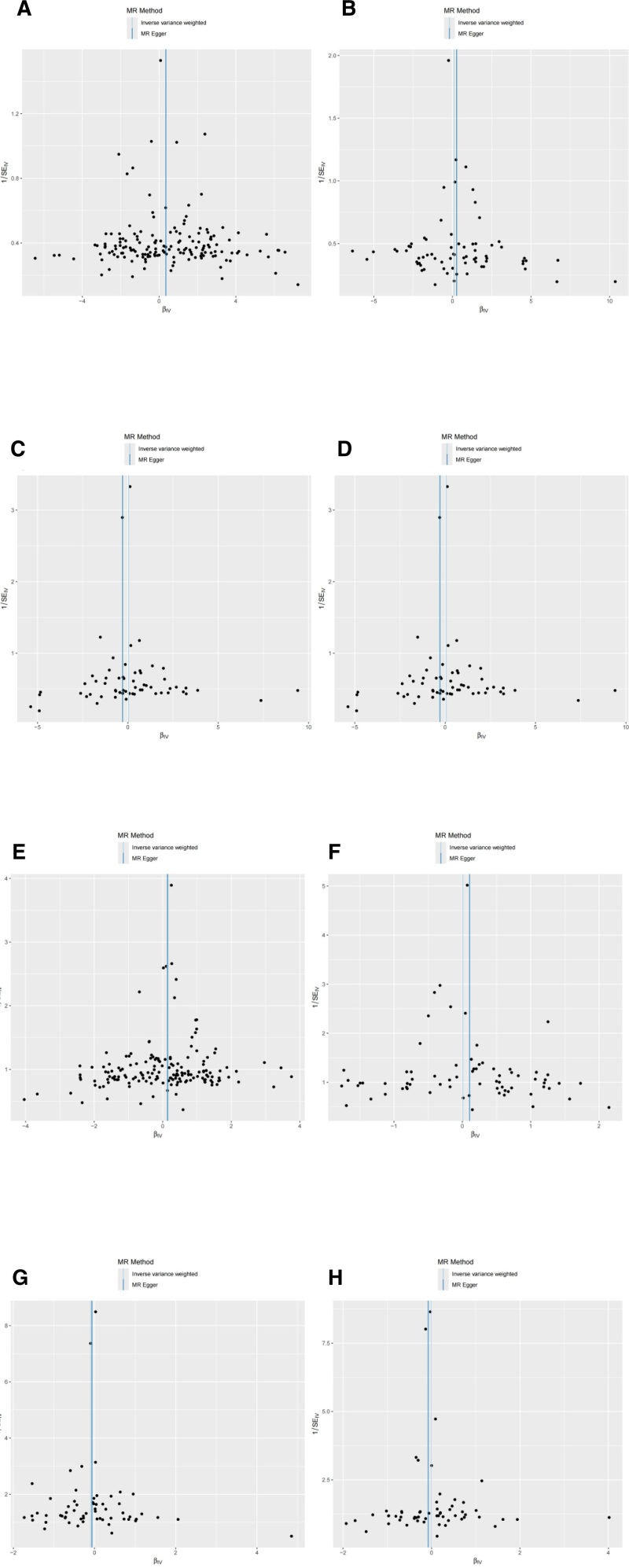
(A) Funnel charts of the causal effect of ferritin on SCLC. (B) Funnel charts of the causal effect of serum on SCLC. (C) Funnel charts of the causal effect of TIBC on SCLC. (D) Funnel charts of the causal effect of TSAT on SCLC. (E) Funnel charts of the causal effect of ferritin on NSCLC. (F) Funnel charts of the causal effect of serum on NSCLC. (G) Funnel charts of the causal effect of TIBC on NSCLC. (H) Funnel charts of the causal effect of TSAT on NSCLC. NSCLC = non-small cell lung cancer, SCLC = small cell lung cancer, TIBC = total iron binding capacity, TSAT = transferrin saturation.

**Figure 4.**
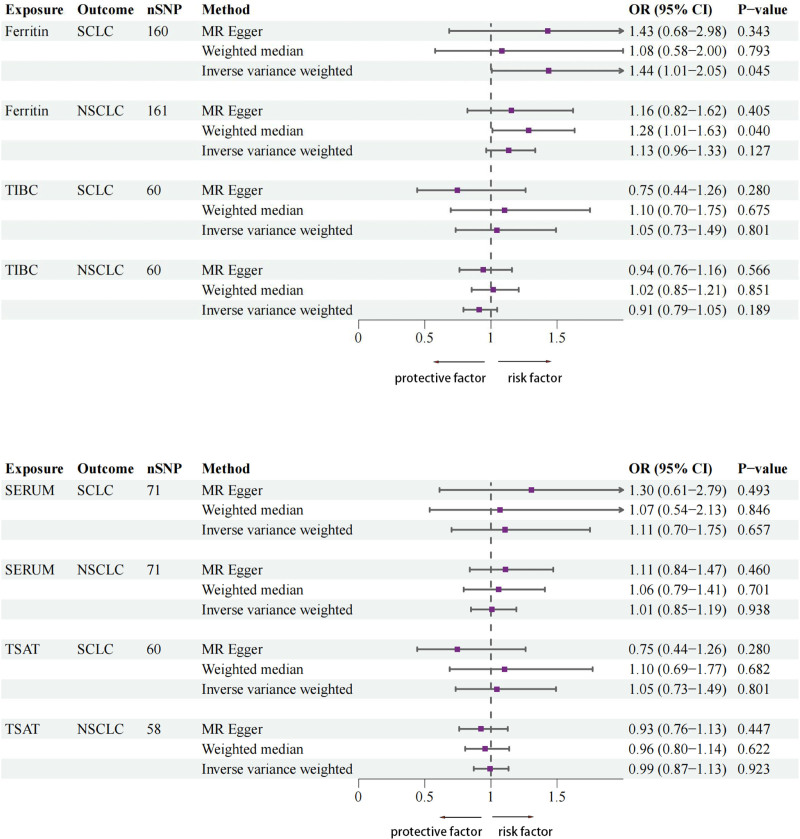
Forest plot of causal effects of iron homeostasis markers (ferritin, TIBC, serum iron, TSAT) on lung cancer and small cell lung cancer. The IVW method is the primary method used for calculating MR (*P*-value < .05 was considered statistically significant). 95% CI = 95% confidence interval, NSCLC = non-small cell lung cancer, OR = odds ratio, SCLC = small cell lung cancer, TIBC = total iron binding capacity, TSAT = transferrin saturation.

The IVW analysis outcomes indicate that ferritin levels (β = 0.351; 95% CI = 1.006–2.046; *P* = .045) have a genetic causal association with SCLC and is a positive correlation with the risk of SCLC. TIBC, TSAT, and serum iron levels do not have a genetic causal association with SCLC (*P* > .05). Additionally, the 4 indicators of iron homeostasis do not have a genetic causal association with NSCLC (*P* > .05). The results are visualized using a scatter plot and funnel charts (Figs. 2 and 3). The specific MR analysis outcome are detailed in (Table 1). The graphical representation of the forest plot distinctly demonstrates that, among the various indices of iron homeostasis, solely the black solid line depicting ferritin stands to the right of the “0” mark, suggesting a positive correlation between elevated ferritin concentrations and an augmented risk of developing SCLC (Fig. 4).

**Table 1:** Analysis results of MR on iron homeostasis and SCLC and NSCLC.

Exposure	Outcome	Method	nSNP	β	95% CI	*P*-val
Ferritin	SCLC	MR Egger	160	0.356	0.684–2.979	.343
		Weighted median	160	0.081	0.588–1.999	.793
		Inverse variance weighted	160	0.361	1.006–2.046	.045
		Simple mode	160	-0.568	0.110–2.890	.495
		Weighted mode	160	-0.097	0.313–2.625	.857
Serum	SCLC	MR Egger	71	0.266	0.611–2.788	.493
		Weighted median	71	0.068	0.537–2.134	.846
		Inverse variance weighted	71	0.103	0.702–1.749	.657
		Simple mode	71	0.476	0.444–5.827	.47
		Weighted mode	71	0.123	0.557–2.295	.734
TIBC	SCLC	MR Egger	60	-0.29	0.442–1.262	.28
		Weighted median	60	0.098	0.696–1.749	.675
		Inverse variance weighted	60	0.045	0.733–1.492	.801
		Simple mode	60	0.016	0.362–2.848	.974
		Weighted mode	60	-0.199	0.640–1.499	.926
TSAT	SCLC	MR Egger	60	-0.29	0.442–1.262	.280
		Weighted median	60	0.098	0.688–1.769	.682
		Inverse variance weighted	60	0.045	0.733–1.492	.801
		Simple mode	60	0.016	0.361–2.857	.974
		Weighted mode	60	-0.019	0.651–1.475	.924
Ferritin	NSCLC	MR Egger	161	0.144	0.822–1.621	.405
		Weighted median	161	0.25	1.011–1.633	.04
		Inverse variance weighted	161	0.126	0.964–1.335	.127
		Simple mode	161	0.325	0.759–2.525	.289
		Weighted mode	161	0.246	0.937–1.745	.122
Serum	NSCLC	MR Egger	71	0.106	0.840–1.471	.46
		Weighted median	71	0.056	0.794–1.408	.701
		Inverse variance weighted	71	0.006	0.849–1.192	.938
		Simple mode	71	0.29	0.777–2.298	.297
		Weighted mode	71	-0.28	0.735–1.285	.844
TIBC	NSCLC	MR Egger	60	-0.061	0.762–1.159	.566
		Weighted median	60	0.016	0.854–1.210	.851
		Inverse variance weighted	60	-0.093	0.792–1.047	.189
		Simple mode	60	-0.109	0.589–1.361	.608
		Weighted mode	60	-0.044	0.817–1.119	.582
TSAT	NSCLC	MR Egger	58	-0.076	0.760–1.127	.447
		Weighted median	58	-0.043	0.805–1.138	.622
		Inverse variance weighted	58	-0.006	0.870–1.133	.923
		Simple mode	58	0.042	0.713–1.524	.827
		Weighted mode	58	-0.059	0.807–1.098	.447

NSCLC = non-small cell lung cancer, nSNP = number of single nucleotide polymorphisms, SCLC = small cell lung cancer, TIBC = total iron binding capacity, TSAT = transferrin saturation.

### 3.2. Reverse MR analysis results

To evaluate the potential impact of reverse causality on the main findings of this study, we conducted a reverse MR analysis. The IVW analysis revealed no genetic causal association between SCLC and ferritin (β = −0.001; 95% CI = 0.993–1.005; *P* = .68). Additionally, the MR Egger and weighted median analyses yielded results consistent with the IVW direction and effect size, thereby supporting the robustness of our findings. The intercept term in the MR Egger regression indicated a *P*-value >.05, suggesting no directional level heterogeneity. Furthermore, both Cochrane *Q* test and Rucker *Q* test showed no significant heterogeneity (*P* > .05), confirming the consistency of the IV estimates. The MR-PRESSO global test also indicated no significant global level heterogeneity (*P* > .05), with no outliers identified. Based on consistent evidence from the primary analysis and a series of sensitivity analyses, we conclude that serum ferritin levels are unlikely to be causally influenced by SCLC. (Tables S10 and S11, Supplemental Digital Content, https://links.lww.com/MD/Q480).

### 3.3. Sensitivity analysis of MR results

The results of the heterogeneity analysis indicate that, across all analyses of iron homeostatic indicators and the risk of SCLC, the *P*-values for both Cochran *Q* test and Rucker *Q* test exceeded 0.05. This suggests the absence of significant heterogeneity among the IVs. In contrast, a highly significant heterogeneity was observed in the analysis of ferritin concerning NSCLC, with Cochran *Q* test yielding a *P*-value of .001 and Rucker *Q* test yielding a *P*-value of .0009. This indicates variations in the magnitude of the effects of genetic IVs on ferritin, potentially mediated by multiple biological pathways. The intercept term in the MR Egger regression exhibited a *P*-value >.05, suggesting the absence of directional horizontal pleiotropy. Furthermore, the global test results corroborate this conclusion, demonstrating that, with the exception of the ferritin NSCLC analysis, all other analyses presented non-significant global test *P*-values (*P* > .05), indicating no severe global horizontal pleiotropy. Detailed results are presented in Table 2.

**Table 2:** Sensitivity analysis results of MR on iron homeostasis with SCLC and NSCLC.

Exposure	Outcome	Heterogeneity test		Pleiotropy test	MR-PRESSO
		Cochran *Q* test (*P*-value) Inverse variance weighted	Rucker *Q* test (*P*-value) MR Egger	Egger intercept (*P*-value) MR Egger	Global test (*P*-value)
FERRITIN	SCLC	.925	.917	.987	.921
SERUM	SCLC	.247	.229	.598	.259
TIBC	SCLC	.052	.077	.096	.075
TSAT	SCLC	.052	.077	.096	.075
FERRITIN	NSCLC	.001	.0009	.906	.001
SERUM	NSCLC	.699	.692	.386	.687
TIBC	NSCLC	.053	.046	.695	.060
TSAT	NSCLC	.111	.11	.347	.105

NSCLC = non-small cell lung cancer, SCLC = small cell lung cancer, TIBC = total iron binding capacity, TSAT = transferrin saturation.

Based on a comprehensive analysis, the examination of ferritin and NSCLC revealed low reliability due to significant heterogeneity and pleiotropy. In contrast, other iron homeostasis biomarkers exhibited no substantial impact from heterogeneity or pleiotropy in their meta-analysis results concerning LC risk. To further validate the robustness of these conclusions, we conducted a leave-one-out analysis. The results indicated that the removal of each SNP individually did not alter the outcomes of the meta-analysis, while the direction of causal estimates remained consistent. This suggests that our primary findings are not influenced by any single SNP, thereby confirming the robustness and reliability of the conclusions (Figures S1–S8, Supplemental Digital Content, https://links.lww.com/MD/Q481).

## 4. Discussion

The core findings of this study indicate that serum ferritin levels exhibit a positive genetic association with the risk of SCLC, as determined through IVW analysis (β = 0.351, 95% CI: 1.006–2.046, *P* = .045). However, after applying FDR correction, this association did not meet the stringent statistical significance threshold (*P*_FDR_ = .18) (Table S12, Supplemental Digital Content, https://links.lww.com/MD/Q480). A review of the literature shows that previous exploratory studies often relaxed the FDR threshold to 0.2.^[45,46]^ Hence this study is fundamentally exploratory, aiming to utilize genetic IVs to preliminarily identify novel causal associations between iron homeostasis and LC risk, rather than to conduct definitive hypothesis verification. The findings provide direction and hypotheses for further research and are consistently supported by multiple complementary analytical methods, including MR-Egger regression, the weighted median method, and the hold-out method, thereby enhancing the credibility of the overall conclusions. Furthermore, the findings are biologically plausible, Wang et al demonstrated that elevated serum ferritin levels correlate with cancer progression and immune regulation.^[50]^ Ramirez-Carmona et al showed that iron overload contributes to oxidative stress and the regulation of the tumor microenvironment, particularly in aggressive cancers such as SCLC.^[51]^ Additionally, the sensitivity analysis revealed no horizontal multivariate effects or significant heterogeneity, further reinforcing the robustness of the association between ferritin and SCLC. Reverse MR analysis also rules out the possibility of reverse causality, specifically that SCLC causes ferritin elevation. Although current evidence is insufficient to establish causality, this finding holds potential biological significance and research value, warranting further validation in larger independent cohorts or future meta-analyses. Additionally, this study did not demonstrate a significant association between ferritin levels and the risk of NSCLC. Other indicators of iron homeostasis, including TIBC and TSAT, also showed no significant correlation with the risk of SCLC or NSCLC. Collectively, these results suggest that serum ferritin may exhibit relatively specific associations with SCLC risk within the iron metabolism pathway. If future studies can confirm a causal relationship, serum ferritin may become a potential specific biomarker for SCLC. Based on existing evidence, we propose the following hypothesis: ferritin may promote the progression of SCLC by influencing cellular iron homeostasis rather than systemic iron balance. Elevated ferritin levels could reflect iron overload in the tumor microenvironment, thereby providing essential nutrients for rapid cancer cell proliferation.

Ferritin is a high molecular weight protein primarily responsible for storing and releasing iron in the body, thereby maintaining intracellular iron balance. It is present in almost all cells, particularly in hepatocytes, splenocytes, and bone marrow cells, and can also be found in small amounts in serum. The importance of ferritin lies not only in its role as the primary means of iron storage but also in its critical roles in various physiological and pathological processes. The measurement of ferritin levels is commonly used to assess the body’s iron storage status. Abnormal serum ferritin levels can be markers of various diseases, such as iron-deficiency anemia, iron overload disorders (thalassemia, hemochromatosis), infections, inflammation, and cancer.^[52–55]^ The specific mechanisms of ferritin in the development of SCLC are not yet fully understood. A meta-analysis showed that serum levels of ferritin in LC patients were higher than in healthy adults, and that these levels increased with disease progression, suggesting that ferritin levels could serve as a biomarker for LC and be a sensitive indicator for detecting advanced-stage tumors.^[51]^ In a study conducted by Fei Wang et al, a cohort of 107 LC patients receiving immune checkpoint inhibitor treatment were appraised. Utilizing electrochemiluminescence techniques, serum ferritin concentrations were quantified pre- and post-treatment, with subsequent analysis exploring their correlation with various treatment response indicators, including response rates, survival durations, PD-L1 expression status, tumor staging, and pathological subtypes. Notably, the investigation revealed that individuals with initially lower serum ferritin levels prior to immunotherapy experienced longer progression-free survival (PFS) intervals and achieved higher disease control rates. Similarly, a decline in serum ferritin levels during therapy was indicative of superior PFS and disease control rates outcomes. A comprehensive analysis incorporating both baseline and treatment-course serum ferritin levels further underscored their association with prolonged PFS. As a result, the investigation determined that serum ferritin concentrations emerge as a significant prognostic indicator for forecasting the effectiveness of immunotherapy in LC patients, potentially aiding in the anticipation of therapeutic responses, as documented in the cited reference.^[50]^ Many epidemiological studies also suggest that a high-iron diet increases the risk of LC.^[19–23]^ As it increases the body’s iron levels, thereby affecting ferritin. Studies have shown that most of the dietary iron absorbed is transported to the liver, which senses the increased iron levels and synthesizes more ferritin to store this iron.^[56]^ This is consistent with our findings from MR analysis, suggesting that ferritin might promote LC progression by affecting iron homeostasis. However, in our study, we found no significant association between iron homeostasis and NSCLC. These robust clinical correlations and epidemiological evidence collectively highlight a central question: through which molecular mechanisms does ferritin contribute to the progression of LC, particularly in SCLC? We hypothesize that dysfunctional ferritin may act as a primary driver of ferroptosis susceptibility in SCLC, a process likely regulated synergistically by the characteristic loss of TP53/RB1 and MYC amplification in this malignancy. In SCLC, the inactivation rates of the TP53 gene range from 75% to 90%, often coexisting with deletions of the RB1 gene. Mouse models have demonstrated that the targeted knockout of both TP53 and RB1 genes can induce tumors that closely resemble human SCLC.^[57]^ Furthermore, the deletion of TP53 and RB1 contributes to chemotherapy resistance in tumor cells, significantly impairing treatment efficacy.^[58]^ Recent studies have highlighted the pivotal role of TP53 in maintaining cellular iron metabolism through the regulation of ferritin synthesis and degradation. Experimental evidence indicates that mutant p53 (mut-p53) diminishes the expression of ferritin heavy chain 1 and the coregulator of nuclear receptor activation factor 4, thus increasing the susceptibility of pancreatic ductal adenocarcinoma cells to ferroptosis.^[59]^ Similarly, in breast cancer, silencing of ferritin heavy chain 1 promotes cell growth and tumor spheroid formation, increases c-MYC expression, and decreases cellular sensitivity to chemotherapy.^[60]^ The MYC gene is frequently amplified in SCLC and other malignancies, showing a significant negative correlation with ferritin levels.^[61–63]^ The mechanism operates in a manner where MYC amplification temporarily upregulates ferritin expression to satisfy elevated metabolic demands. Concurrently, it activates iron uptake pathways, such as transferrin receptor, while accelerating iron consumption. This process leads to a rapid depletion of ferritin storage, effectively converting it into a source of free iron release. This imbalance in iron homeostasis may indirectly promote tumor progression by inducing oxidative stress and increasing susceptibility to ferroptosis, thereby favoring the selection of drug-resistant cancer cell clones.^[64]^ It is regrettable that research on ferritin dysregulation in SCLC remains underexplored, necessitating further investigation to elucidate its mechanisms of action in SCLC. In contrast, NSCLC exhibits distinct progression mechanisms due to the lack of dynamic ferritin reprogramming. This fundamental difference in ferritin regulation mechanisms opens new avenues for precision treatment strategies in SCLC. Therefore, further research is required to clarify how iron homeostasis influences different types of LC, thereby providing a comprehensive understanding of the complex role of iron in the development and progression of lung malignancies.

MR research starts from the genetic perspective by using GWAS databases to select candidate genes and introduce IVs that are strongly associated with the exposure factors, namely, SNPs. The types of the SNPs are allocated to a person prior to being influenced by exposure or results, making them unchangeable and eliminating the influence of extraneous variables from cellular transcription processes. Therefore, it allows for a more accurate evaluation of the association between exposure and outcomes, and can even be used to confirm the results of randomized controlled trials (RCTs). RCTs, as prospective studies, are essential for evaluating causal relationships, however, they are not appropriate for all research contexts because of challenges related to implementation and ethic.^[25–27]^ In contrast, observational studies cannot generally provide true causal association results between exposure and outcomes through regression methods due to the uncontrollable nature of confounding factor.^[65]^

Thus, employing MR analysis not only mitigates the influence of confounding variables and reverse causality, but also boasts superior feasibility and a heightened evaluative merit. Consequently, utilizing this methodology to delve into the nexus between iron regulation and LC holds promise for generating genetically robust and reliable findings.

However, this study has several limitations. Firstly, our GWAS data were exclusively sourced from European populations, with no inclusion of other ethnic groups. Even within the same racial group, analytical results may vary across different databases. Consequently, the generalizability of these findings is restricted to the specific database and population analyzed.

Secondly in this study, the positive association between ferritin and SCLC was quantified, revealing an IVW odds ratio of 1.44 (95% CI: 1.01–2.05). The broad CI indicates a lack of statistical precision and heightened uncertainty, primarily due to 2 factors: limited valid sample size: despite utilizing aggregated data from extensive biobanks such as FinnGen and deCODE, the relatively small number of SCLC cases (n = 855) diminished statistical power, thereby naturally widening the CI; complex phenotypic nature: both iron homeostasis and LC are polygenic traits, influenced by a multitude of genetic and environmental factors, with heterogeneity further complicating causal estimations. Nonetheless, various MR methods (IVW, MR Egger, weighted median) and a series of sensitivity analyses (leave-one-out method, heterogeneity testing, and multivariate analysis of covariance) consistently corroborated the primary findings, providing robust support for the conclusions drawn.

The current limitations in the data are further underscored by the absence of detailed histological annotations. The meta-analysis presented in version R11 of the FinnGen database did not yield reliable subtypes for NSCLC, which includes adenocarcinoma, squamous cell carcinoma, and large cell carcinoma. This shortcoming hinders our ability to ascertain whether the association between ferritin and SCLC is confined to SCLC subtypes or if it extends to other specific subtypes through stratified analysis. Future research should fully leverage individual data from the most recent versions of the FinnGen database or The Cancer Genome Atlas, or utilize the comprehensive phenotypic data from the UK Biobank to systematically assess the influence of confounding factors such as body mass index, smoking status, and inflammatory status on the estimated values.

Third, while the threshold for SNP selection (*P* < 1 × 10⁻^5^) provides sufficient IVs under the constraint of an *F*-value >10, the small marginal effect values of SNPs, along with multicollinearity and wider CIs, may still understate the true causal effect. Although we validated the consistency of result directions through sensitivity analyses, including the hold-out method and weighted median estimation, further replication in larger GWAS samples is necessary to narrow CIs and enhance instrumental strength.

Fourth, we employed MR Egger regression and MR-PRESSO to detect and correct for horizontal pleiotropy; neither test indicated significant directional pleiotropy. We further acknowledge that multivariable MR (MVMR) can simultaneously adjust for multiple correlated exposures, thereby attenuating residual pleiotropy and isolating the specific causal effect of ferritin. Owing to current limitations in the accessibility and harmonization of GWAS summary statistics, MVMR analyses could not be implemented in the present study. Upon acquisition of comprehensive GWAS data, MVMR will be prioritized to corroborate the present findings.

Fifth, this study utilized serum ferritin as a genetic proxy for iron storage, without concurrently assessing inflammatory markers (e.g., CRP, IL-6) or distinguishing between ferritin subtypes (e.g., glycosylated ferritin). This limitation complicates the exclusion of the possibility that elevated ferritin levels may result from inflammatory responses. Future research should consider incorporating inflammatory factors such as IL-6 and CRP as covariates in multivariate meta-analyses or establishing longitudinal cohorts to dynamically compare changes in ferritin levels between precancerous lesions (e.g., pulmonary intraepithelial neoplasia) and purely inflammatory states, thereby clarifying the relative contributions of “iron storage” and “acute phase response.” Although reverse MR analysis does not support reverse causality, we cannot entirely dismiss the possibility that undiagnosed early-stage SCLC may affect ferritin levels through inflammatory or metabolic feedback mechanisms. As this study relies on cross-sectional GWAS data, it does not capture dynamic changes in ferritin levels prior to tumor development. Future studies that combine prospective cohort research or early tumor multi-omics data will help elucidate the temporal progression and functional roles of ferritin in SCLC pathogenesis.

## 5. Conclusion

In conclusion, this study provides the first genetic-level evidence through bidirectional 2-sample MR analysis, confirming that elevated serum ferritin independently and unidirectionally drives SCLC, with no significant association observed in NSCLC or other iron metabolism biomarkers. This causal link can be partially attributed to iron overload-induced oxidative stress, ferroptosis escape in the context of TP53/RB1 deficiency, and MYC-driven metabolic reprogramming. Reverse MR analysis further rules out the possibility of reverse causality, specifically that SCLC causes ferritin elevation. Clinically, serum ferritin serves as a practical biomarker for early screening and risk stratification, supporting the initiation of low-dose CT screening for individuals with persistently high levels, the incorporation of approved iron chelators or hepcidin modulators into high-risk LC prevention trials, and the use of ferritin dynamics as real-time efficacy biomarkers in immunotherapy. Notably, after conducting multiple testing corrections, the FDR for this association remained below the 0.2 exploration threshold, indicating that the findings are preliminary. Future studies should aim to validate the specific association between ferritin and SCLC, a subtype of aggressive LC, across diverse ethnic populations. Integrating prospective cohort studies, functional experiments, and RCTs will facilitate the translation of these genetic findings into precise preventive strategies and personalized therapeutic targets.

## Acknowledgments

We appreciate the generosity and contribution of all participants and relevant GWAS databases.

## Author contributions

**Conceptualization:** Zhuien Wang.

**Data curation:** Xin Sun.

**Formal analysis:** Liqiu Yu, Yi Yang.

**Investigation:** Liqiu Yu, Yi Yang.

**Methodology:** Liqiu Yu.

**Project administration:** Zhuien Wang.

**Software:** Liqiu Yu.

**Supervision:** Zhuien Wang.

**Validation:** Liqiu Yu, Xin Sun.

**Visualization:** Liqiu Yu.

**Writing – original draft:** Liqiu Yu, Chengye Chen, Mengfan Li.

**Writing – review & editing:** Chengye Chen, Mengfan Li.

## Supplementary Material


