# Supplementary material for: Investigating the genetic causal link between iron regulation and lung cancer risk: A 2-sample Mendelian randomization analysis
Source: Medicine (Baltimore). 2025 Oct 24;104(43):e45518. doi: 10.1097/MD.0000000000045518 (PMC12558328; doi:10.1097/MD.0000000000045518)
Supplement: Supplementary file 1 [file medi-104-e45518-s001.docx]

| **Supplementary Table 1 Descriptions of datasets included in this study.** | | | | | | |
| --- | --- | --- | --- | --- | --- | --- |
| **Exposure** | **Abbreviation** | **Phenotype** | **Sample Size** | **Ancestry** | **Web Source** | **Reference** |
| Ferritin | Fe | Ferritin | 246,139 participants | European | <https://www.decode.com/summarydata/> | [1] |
| Serum Iron | Serum | Serum Iron | 163,511 participants | European | <https://www.decode.com/summarydata/> | [1] |
| Total Iron Binding Capacity | TIBC | Total Iron Binding Capacity | 135,430 participants | European | <https://www.decode.com/summarydata/> | [1] |
| Transferrin Saturation | TSAT | Transferrin Saturation | 131,471 participants | European | <https://www.decode.com/summarydata/> | [1] |
| **Outcome** | **Abbreviation** | **Phenotype** | **Sample Size** | **Ancestry** | **Web Source** | **Reference** |
| small cell lung cancer | SCLC | small cell lung cancer | 345,973 participants | Finnish | <https://www.finngen.fi/en/> | [2] |
| non-small cell lung cancer | NSCLC | non-small cell lung cancer | 347,182 participants | Finnish | <https://www.finngen.fi/en/> | [2] |
| **References** | | | | | | |
| [1] Bell S, Rigas AS, Magnusson MK, et al. A genome-wide meta-analysis yields 46 new loci associating with biomarkers of iron homeostasis. Commun Biol. 2021;4(1):156.  [2] Kurki MI, Karjalainen J, Palta P, et al. FinnGen provides genetic insights from a well-phenotyped isolated population. Nature. 2023;613(7944):508-518. | | | | | | |

| **Supplementary Table 2 Statistical characteristics of eligible instrumental variables in forward MR analysis.** | | | | | | | | | | | | | | | | | | |
| --- | --- | --- | --- | --- | --- | --- | --- | --- | --- | --- | --- | --- | --- | --- | --- | --- | --- | --- |
| **FERRITIN AND SCLC** | | | | | | | | | | | | | | | | | | |
| **id.exposure** | **exposure** | **id.outcome** | **outcome** | **SNP** | **effect_allele.exposure** | **other_allele.exposure** | **effect_allele.outcome** | **other_allele.outcome** | **beta.exposure** | **beta.outcome** | **eaf.exposure** | **eaf.outcome** | **pval.outcome** | **se.outcome** | **pval.exposure** | **se.exposure** | **F** | **R^2^** |
| m6enaM | FERRITIN | iLY8vs | SCLC | rs10184004 | T | C | T | C | -0.0159 | 0.0133997 | 0.4707 | 0.358247 | 0.79208 | 0.0508314 | 5.13E-06 | 0.003487301 | 20.78812789 | 0.000125971 |
| m6enaM | FERRITIN | iLY8vs | SCLC | rs10220355 | A | T | A | T | 0.0157 | 0.0229645 | 0.4252 | 0.42462 | 0.642313 | 0.0494427 | 7.29E-06 | 0.003500526 | 20.11558603 | 0.000120487 |
| m6enaM | FERRITIN | iLY8vs | SCLC | rs10263500 | T | C | T | C | 0.0225 | 0.0465219 | 0.4605 | 0.498523 | 0.341004 | 0.0488582 | 6.59E-11 | 0.003445792 | 42.63702748 | 0.000251545 |
| m6enaM | FERRITIN | iLY8vs | SCLC | rs1065853 | T | G | T | G | 0.0361 | 0.137043 | 0.0594 | 0.0519569 | 0.199031 | 0.106704 | 1.25E-07 | 0.006829323 | 27.94210347 | 0.000145625 |
| m6enaM | FERRITIN | iLY8vs | SCLC | rs107421 | G | A | G | A | -0.0224 | 0.0297881 | 0.2209 | 0.182225 | 0.634577 | 0.0626727 | 1.15E-07 | 0.004225403 | 28.10345819 | 0.000172709 |
| m6enaM | FERRITIN | iLY8vs | SCLC | rs10750215 | T | G | T | G | 0.0213 | -0.0432559 | 0.4199 | 0.393325 | 0.386399 | 0.0499395 | 3.66E-08 | 0.003868143 | 30.32174317 | 0.000221023 |
| m6enaM | FERRITIN | iLY8vs | SCLC | rs10801913 | A | G | A | G | 0.0237 | 0.0677176 | 0.3004 | 0.301033 | 0.203913 | 0.0533007 | 2.63E-10 | 0.003750502 | 39.93170824 | 0.000236089 |
| m6enaM | FERRITIN | iLY8vs | SCLC | rs10804630 | C | T | C | T | -0.0335 | -0.0835404 | 0.1022 | 0.144865 | 0.227911 | 0.0692846 | 3.71E-09 | 0.005681211 | 34.77024698 | 0.000205944 |
| m6enaM | FERRITIN | iLY8vs | SCLC | rs10838599 | T | G | T | G | -0.0182 | -0.103596 | 0.2579 | 0.227063 | 0.0738839 | 0.057961 | 3.85E-06 | 0.003939974 | 21.33811563 | 0.00012679 |
| m6enaM | FERRITIN | iLY8vs | SCLC | rs10842942 | G | A | G | A | -0.0192 | 0.027119 | 0.2173 | 0.155962 | 0.685141 | 0.066885 | 5.19E-06 | 0.004213338 | 20.7658569 | 0.000125397 |
| m6enaM | FERRITIN | iLY8vs | SCLC | rs11125069 | A | G | A | G | 0.0157 | -0.00908155 | 0.4607 | 0.404311 | 0.855273 | 0.0497908 | 6.41E-06 | 0.003479306 | 20.36170086 | 0.000122484 |
| m6enaM | FERRITIN | iLY8vs | SCLC | rs111405904 | A | G | A | G | 0.0798 | -0.0273483 | 0.0109 | 0.019496 | 0.878113 | 0.178325 | 1.76E-06 | 0.016697379 | 22.84068754 | 0.00013731 |
| m6enaM | FERRITIN | iLY8vs | SCLC | rs1121985 | A | C | A | C | 0.018 | 0.0297872 | 0.3231 | 0.324907 | 0.569601 | 0.0523832 | 1.12E-06 | 0.003696638 | 23.70997866 | 0.000141722 |
| m6enaM | FERRITIN | iLY8vs | SCLC | rs112229166 | T | G | T | G | 0.0224 | -0.0162044 | 0.1285 | 0.153945 | 0.811564 | 0.0679692 | 9.02E-06 | 0.0050457 | 19.7084825 | 0.000112382 |
| m6enaM | FERRITIN | iLY8vs | SCLC | rs11230848 | T | C | T | C | -0.031 | 0.0770639 | 0.0895 | 0.155852 | 0.256143 | 0.0678645 | 2.98E-07 | 0.006049126 | 26.26262462 | 0.000156623 |
| m6enaM | FERRITIN | iLY8vs | SCLC | rs112579095 | T | C | T | C | 0.0357 | -0.0620417 | 0.0596 | 0.0878368 | 0.47214 | 0.0862887 | 3.83E-07 | 0.007031409 | 25.7781479 | 0.000142865 |
| m6enaM | FERRITIN | iLY8vs | SCLC | rs114007478 | G | A | G | A | 0.0379 | 0.000284829 | 0.0535 | 0.0470246 | 0.998049 | 0.116501 | 2.73E-06 | 0.008080759 | 21.99753973 | 0.000145473 |
| m6enaM | FERRITIN | iLY8vs | SCLC | rs11614077 | T | C | T | C | 0.022 | 0.0595043 | 0.2255 | 0.329848 | 0.253039 | 0.0520599 | 6.59E-08 | 0.004072562 | 29.18165726 | 0.000169061 |
| m6enaM | FERRITIN | iLY8vs | SCLC | rs11634990 | C | T | C | T | -0.0265 | -0.0465919 | 0.1708 | 0.167887 | 0.469633 | 0.0644358 | 9.40E-09 | 0.004615748 | 32.96154556 | 0.000198916 |
| m6enaM | FERRITIN | iLY8vs | SCLC | rs11708600 | T | C | T | C | 0.03 | 0.0187547 | 0.0865 | 0.108868 | 0.812726 | 0.079164 | 1.53E-06 | 0.006240539 | 23.10991277 | 0.000142232 |
| m6enaM | FERRITIN | iLY8vs | SCLC | rs117940828 | C | G | C | G | -0.0641 | 0.0486924 | 0.0206 | 0.00821409 | 0.85469 | 0.265879 | 4.41E-07 | 0.012692188 | 25.50604176 | 0.000165796 |
| m6enaM | FERRITIN | iLY8vs | SCLC | rs11977873 | G | A | G | A | -0.0276 | 0.0775864 | 0.1463 | 0.123041 | 0.29405 | 0.0739426 | 6.76E-08 | 0.005113539 | 29.13231646 | 0.000190282 |
| m6enaM | FERRITIN | iLY8vs | SCLC | rs12200559 | T | C | T | C | 0.0189 | 0.0555129 | 0.2305 | 0.217991 | 0.345598 | 0.0588582 | 4.94E-06 | 0.004138094 | 20.86041311 | 0.000126716 |
| m6enaM | FERRITIN | iLY8vs | SCLC | rs12419620 | G | T | G | T | -0.0312 | -0.0494831 | 0.1621 | 0.142174 | 0.486456 | 0.0711011 | 3.43E-11 | 0.004708142 | 43.91471638 | 0.000264432 |
| m6enaM | FERRITIN | iLY8vs | SCLC | rs1250244 | G | C | G | C | -0.0247 | -0.0511051 | 0.3476 | 0.200487 | 0.400273 | 0.0607573 | 3.56E-11 | 0.003730373 | 43.84190214 | 0.000276705 |
| m6enaM | FERRITIN | iLY8vs | SCLC | rs12568930 | C | T | C | T | 0.0345 | -0.0349259 | 0.185 | 0.0899993 | 0.68363 | 0.0857044 | 1.88E-14 | 0.004504751 | 58.65386151 | 0.00035892 |
| m6enaM | FERRITIN | iLY8vs | SCLC | rs1260326 | T | C | T | C | 0.0254 | 0.0843253 | 0.3414 | 0.349905 | 0.0998504 | 0.0512437 | 1.48E-12 | 0.003589421 | 50.07473211 | 0.000290123 |
| m6enaM | FERRITIN | iLY8vs | SCLC | rs12685912 | A | C | A | C | 0.0193 | -0.00881467 | 0.2185 | 0.249307 | 0.875965 | 0.0564733 | 3.58E-06 | 0.004164522 | 21.47752814 | 0.000127211 |
| m6enaM | FERRITIN | iLY8vs | SCLC | rs12807014 | C | T | C | T | -0.0286 | -0.0613257 | 0.2431 | 0.223088 | 0.292036 | 0.0582022 | 2.72E-13 | 0.003913745 | 53.40070795 | 0.000301013 |
| m6enaM | FERRITIN | iLY8vs | SCLC | rs12892462 | G | C | G | C | 0.0186 | -0.0266828 | 0.3278 | 0.326903 | 0.609926 | 0.0523009 | 4.43E-07 | 0.003683543 | 25.49731213 | 0.000152463 |
| m6enaM | FERRITIN | iLY8vs | SCLC | rs12916360 | T | G | T | G | -0.027 | -0.0160461 | 0.1601 | 0.228781 | 0.781601 | 0.0578793 | 1.98E-08 | 0.004809628 | 31.51407425 | 0.000196054 |
| m6enaM | FERRITIN | iLY8vs | SCLC | rs12979144 | A | G | A | G | 0.0197 | -0.00392485 | 0.3689 | 0.440987 | 0.936328 | 0.0491304 | 2.68E-08 | 0.003542432 | 30.92640277 | 0.000180705 |
| m6enaM | FERRITIN | iLY8vs | SCLC | rs13253974 | A | G | A | G | 0.0244 | -0.0674536 | 0.3396 | 0.291803 | 0.206228 | 0.053365 | 2.51E-11 | 0.003656646 | 44.52601375 | 0.000267045 |
| m6enaM | FERRITIN | iLY8vs | SCLC | rs13408333 | T | A | T | A | -0.0287 | -0.069082 | 0.0951 | 0.0888381 | 0.410469 | 0.0839321 | 1.64E-06 | 0.005987433 | 22.97642523 | 0.000141767 |
| m6enaM | FERRITIN | iLY8vs | SCLC | rs139977145 | T | G | T | G | 0.0288 | 0.0312644 | 0.0771 | 0.190472 | 0.618241 | 0.0627365 | 6.71E-06 | 0.006396182 | 20.27418242 | 0.000118039 |
| m6enaM | FERRITIN | iLY8vs | SCLC | rs143854232 | G | A | G | A | 0.0834 | -0.0454647 | 0.0112 | 0.0132011 | 0.8375 | 0.221679 | 7.98E-06 | 0.018675601 | 19.94265278 | 0.00015406 |
| m6enaM | FERRITIN | iLY8vs | SCLC | rs143922167 | A | G | A | G | 0.1148 | -0.146556 | 0.015 | 0.00951892 | 0.559853 | 0.251356 | 7.69E-16 | 0.014244884 | 64.94802394 | 0.000389441 |
| m6enaM | FERRITIN | iLY8vs | SCLC | rs144861591 | T | C | T | C | 0.1333 | -0.0532474 | 0.0671 | 0.0377865 | 0.68121 | 0.129614 | 7.35E-85 | 0.006828715 | 381.0503138 | 0.002224579 |
| m6enaM | FERRITIN | iLY8vs | SCLC | rs161044 | T | C | T | C | 0.0731 | 0.302117 | 0.0415 | 0.023466 | 0.0555776 | 0.157819 | 1.25E-19 | 0.00806429 | 82.16795527 | 0.000425114 |
| m6enaM | FERRITIN | iLY8vs | SCLC | rs165316 | G | A | G | A | 0.0226 | -0.0123999 | 0.2224 | 0.214968 | 0.836403 | 0.0600489 | 8.18E-08 | 0.004213971 | 28.7629743 | 0.00017666 |
| m6enaM | FERRITIN | iLY8vs | SCLC | rs16932140 | G | A | G | A | 0.0412 | 0.13218 | 0.0464 | 0.0218651 | 0.423114 | 0.165012 | 3.89E-07 | 0.008119405 | 25.74814953 | 0.000150213 |
| m6enaM | FERRITIN | iLY8vs | SCLC | rs1694067 | T | C | T | C | -0.0211 | 0.00339236 | 0.3983 | 0.371521 | 0.946806 | 0.050846 | 2.53E-09 | 0.003540561 | 35.5157302 | 0.000213395 |
| m6enaM | FERRITIN | iLY8vs | SCLC | rs17050272 | A | G | A | G | 0.0208 | 0.0557528 | 0.4169 | 0.537037 | 0.254529 | 0.048931 | 4.13E-09 | 0.003538076 | 34.56148184 | 0.000210345 |
| m6enaM | FERRITIN | iLY8vs | SCLC | rs17112021 | G | T | G | T | 0.0244 | 0.0249343 | 0.2784 | 0.202806 | 0.679457 | 0.0603439 | 7.66E-10 | 0.003966308 | 37.84484936 | 0.000239208 |
| m6enaM | FERRITIN | iLY8vs | SCLC | rs174548 | G | C | G | C | -0.0193 | -0.0494199 | 0.3532 | 0.390199 | 0.320867 | 0.0497844 | 1.30E-07 | 0.003656102 | 27.86621677 | 0.000170191 |
| m6enaM | FERRITIN | iLY8vs | SCLC | rs17476364 | C | T | C | T | 0.0431 | 0.241273 | 0.0948 | 0.0588654 | 0.0110146 | 0.0949058 | 3.57E-14 | 0.00568919 | 57.39230461 | 0.000318814 |
| m6enaM | FERRITIN | iLY8vs | SCLC | rs17505399 | G | A | G | A | 0.0647 | 0.0525063 | 0.015 | 0.0357376 | 0.688632 | 0.131032 | 5.05E-06 | 0.014180201 | 20.81822929 | 0.000123699 |
| m6enaM | FERRITIN | iLY8vs | SCLC | rs17540759 | T | A | T | A | 0.0435 | 0.142759 | 0.0424 | 0.00997825 | 0.555581 | 0.242202 | 5.70E-07 | 0.008698062 | 25.01114165 | 0.000153659 |
| m6enaM | FERRITIN | iLY8vs | SCLC | rs1765028 | G | A | G | A | -0.0186 | -0.00470138 | 0.4679 | 0.474136 | 0.923226 | 0.0487845 | 7.54E-08 | 0.003458661 | 28.92077288 | 0.000172267 |
| m6enaM | FERRITIN | iLY8vs | SCLC | rs17676097 | G | A | G | A | -0.0272 | -0.0352014 | 0.3577 | 0.32083 | 0.502329 | 0.0524744 | 6.06E-14 | 0.003623392 | 56.35171863 | 0.000339958 |
| m6enaM | FERRITIN | iLY8vs | SCLC | rs181804134 | G | T | G | T | 0.0488 | -0.217031 | 0.0303 | 0.0224171 | 0.179448 | 0.161667 | 7.89E-06 | 0.010921753 | 19.96433933 | 0.000139943 |
| m6enaM | FERRITIN | iLY8vs | SCLC | rs1848797 | G | A | G | A | -0.0167 | -0.0765072 | 0.4119 | 0.357276 | 0.134461 | 0.0511159 | 1.85E-06 | 0.003501668 | 22.74484636 | 0.000135116 |
| m6enaM | FERRITIN | iLY8vs | SCLC | rs1848887 | C | T | C | T | -0.0706 | -0.0439071 | 0.02 | 0.00655016 | 0.886625 | 0.307954 | 5.61E-08 | 0.012999911 | 29.49365827 | 0.000195387 |
| m6enaM | FERRITIN | iLY8vs | SCLC | rs1894692 | G | A | G | A | 0.1472 | -0.245082 | 0.019 | 0.020101 | 0.168111 | 0.177815 | 1.63E-37 | 0.011499594 | 163.8515691 | 0.000807734 |
| m6enaM | FERRITIN | iLY8vs | SCLC | rs191085215 | C | T | C | T | -0.0505 | -0.306703 | 0.0232 | 0.0104375 | 0.196438 | 0.237429 | 3.31E-06 | 0.010858857 | 21.62790874 | 0.000115586 |
| m6enaM | FERRITIN | iLY8vs | SCLC | rs191983110 | G | A | G | A | -0.0509 | -0.0367521 | 0.0271 | 0.0159987 | 0.851377 | 0.196156 | 5.39E-06 | 0.011189259 | 20.69345044 | 0.000136616 |
| m6enaM | FERRITIN | iLY8vs | SCLC | rs1929524 | T | A | T | A | -0.0183 | 0.00321892 | 0.3754 | 0.377767 | 0.948937 | 0.0502634 | 2.41E-07 | 0.003543378 | 26.67271415 | 0.000157047 |
| m6enaM | FERRITIN | iLY8vs | SCLC | rs199138 | A | G | A | G | -0.1464 | -0.0115439 | 0.0815 | 0.0707680000000001 | 0.904041 | 0.0957535 | 5.02E-115 | 0.00642217 | 519.6591259 | 0.003208846 |
| m6enaM | FERRITIN | iLY8vs | SCLC | rs2008598 | G | A | G | A | -0.0205 | 0.00156778 | 0.3355 | 0.180411 | 0.980287 | 0.0634479 | 2.95E-08 | 0.003697439 | 30.74012906 | 0.000187381 |
| m6enaM | FERRITIN | iLY8vs | SCLC | rs200896712 | C | A | C | A | -0.0416 | 0.0395768 | 0.0124 | 0.082141 | 0.654121 | 0.0883324 | 9.90E-07 | 0.008500872 | 23.94747383 | 4.23857E-05 |
| m6enaM | FERRITIN | iLY8vs | SCLC | rs2049722 | G | C | G | C | -0.0156 | 0.0970959 | 0.4284 | 0.435592 | 0.0500369 | 0.0495475 | 7.81E-06 | 0.003489677 | 19.98383037 | 0.000119185 |
| m6enaM | FERRITIN | iLY8vs | SCLC | rs2062059 | G | A | G | A | 0.0177 | 0.0180311 | 0.4535 | 0.426223 | 0.71431 | 0.0492554 | 3.48E-07 | 0.003473719 | 25.96313697 | 0.00015529 |
| m6enaM | FERRITIN | iLY8vs | SCLC | rs2072576 | C | G | C | G | 0.0256 | 0.0694456 | 0.1173 | 0.12007 | 0.357679 | 0.0755009 | 6.41E-06 | 0.005673264 | 20.36169574 | 0.000135713 |
| m6enaM | FERRITIN | iLY8vs | SCLC | rs2093202 | G | A | G | A | 0.0166 | 0.0211319 | 0.3503 | 0.430483 | 0.66787 | 0.0492501 | 3.53E-06 | 0.003579674 | 21.50449367 | 0.000125429 |
| m6enaM | FERRITIN | iLY8vs | SCLC | rs2126588 | G | A | G | A | 0.0177 | 0.109556 | 0.3995 | 0.406039 | 0.0278311 | 0.0498059 | 4.31E-07 | 0.003501671 | 25.55029118 | 0.000150316 |
| m6enaM | FERRITIN | iLY8vs | SCLC | rs2236953 | T | G | T | G | 0.0247 | 0.0347617 | 0.1129 | 0.157519 | 0.605006 | 0.0672094 | 3.01E-06 | 0.005288925 | 21.81017195 | 0.000122205 |
| m6enaM | FERRITIN | iLY8vs | SCLC | rs2529440 | T | C | T | C | -0.0346 | 0.0169984 | 0.4503 | 0.402032 | 0.731973 | 0.0496298 | 4.60E-23 | 0.003498481 | 97.81222953 | 0.000592666 |
| m6enaM | FERRITIN | iLY8vs | SCLC | rs2595582 | A | G | A | G | -0.0195 | 0.0338011 | 0.4151 | 0.329768 | 0.513902 | 0.0517807 | 2.75E-08 | 0.003509308 | 30.87637095 | 0.000184643 |
| m6enaM | FERRITIN | iLY8vs | SCLC | rs2606177 | A | G | A | G | -0.0167 | 0.00393826 | 0.3495 | 0.368319 | 0.938043 | 0.0506663 | 3.44E-06 | 0.003597098 | 21.554026 | 0.000126811 |
| m6enaM | FERRITIN | iLY8vs | SCLC | rs28406133 | G | C | G | C | -0.1036 | 0.143487 | 0.0061 | 0.00168431 | 0.790644 | 0.540492 | 4.13E-06 | 0.022498601 | 21.20354534 | 0.000130143 |
| m6enaM | FERRITIN | iLY8vs | SCLC | rs28590023 | G | A | G | A | 0.0184 | 0.0064477 | 0.4754 | 0.490968 | 0.895277 | 0.0489836 | 9.89E-08 | 0.003452978 | 28.39540212 | 0.00016887 |
| m6enaM | FERRITIN | iLY8vs | SCLC | rs28678167 | T | G | T | G | 0.0192 | -0.0538405 | 0.2673 | 0.397155 | 0.286258 | 0.0504896 | 4.92E-07 | 0.003817548 | 25.29492783 | 0.000144397 |
| m6enaM | FERRITIN | iLY8vs | SCLC | rs28715334 | T | G | T | G | 0.025 | 0.0577309 | 0.2041 | 0.153139 | 0.394099 | 0.0677427 | 8.22E-09 | 0.004337353 | 33.22235994 | 0.000203054 |
| m6enaM | FERRITIN | iLY8vs | SCLC | rs2943644 | T | C | T | C | -0.0185 | -0.0484386 | 0.4791 | 0.563056 | 0.326519 | 0.0493693 | 9.16E-08 | 0.003462704 | 28.54386111 | 0.000170826 |
| m6enaM | FERRITIN | iLY8vs | SCLC | rs2954029 | T | A | T | A | -0.0245 | -0.0333792 | 0.4905 | 0.458734 | 0.493767 | 0.0487764 | 1.42E-12 | 0.003459432 | 50.15596285 | 0.000300017 |
| m6enaM | FERRITIN | iLY8vs | SCLC | rs31474 | C | T | C | T | 0.0203 | -0.0044723 | 0.1891 | 0.379238 | 0.929191 | 0.0503282 | 2.14E-06 | 0.004282942 | 22.46506552 | 0.000126381 |
| m6enaM | FERRITIN | iLY8vs | SCLC | rs34220530 | T | C | T | C | -0.0152 | -0.0116767 | 0.4856 | 0.468967 | 0.810744 | 0.0487614 | 9.70E-06 | 0.003435995 | 19.56960814 | 0.000115424 |
| m6enaM | FERRITIN | iLY8vs | SCLC | rs34523089 | T | C | T | C | 0.0686 | 0.163721 | 0.1637 | 0.157893 | 0.0104124 | 0.0639081 | 3.16E-48 | 0.00470122 | 212.9252085 | 0.001288514 |
| m6enaM | FERRITIN | iLY8vs | SCLC | rs34787647 | G | A | G | A | 0.0192 | -0.027992 | 0.27 | 0.283095 | 0.604529 | 0.0540492 | 5.11E-07 | 0.003823075 | 25.2218432 | 0.000145318 |
| m6enaM | FERRITIN | iLY8vs | SCLC | rs34877685 | A | G | A | G | 0.0633 | 0.457991 | 0.0181 | 0.00325896 | 0.303219 | 0.444843 | 4.35E-07 | 0.012527294 | 25.53247294 | 0.000142424 |
| m6enaM | FERRITIN | iLY8vs | SCLC | rs350415 | G | A | G | A | -0.0201 | -0.0835839 | 0.2726 | 0.228374 | 0.147385 | 0.0576906 | 2.56E-07 | 0.003900445 | 26.5560696 | 0.000160222 |
| m6enaM | FERRITIN | iLY8vs | SCLC | rs35107257 | A | G | A | G | 0.0431 | -0.223983 | 0.0591 | 0.0368115 | 0.0913335 | 0.132659 | 3.27E-08 | 0.007799028 | 30.54031986 | 0.000206593 |
| m6enaM | FERRITIN | iLY8vs | SCLC | rs35583916 | A | G | A | G | 0.0671 | -0.121028 | 0.0113 | 0.0131576 | 0.573049 | 0.214754 | 6.46E-06 | 0.014875588 | 20.34683004 | 0.000100605 |
| m6enaM | FERRITIN | iLY8vs | SCLC | rs358383 | A | G | A | G | -0.0366 | 0.108977 | 0.0423 | 0.0400341 | 0.378358 | 0.123707 | 7.99E-06 | 0.008196259 | 19.94026032 | 0.000108533 |
| m6enaM | FERRITIN | iLY8vs | SCLC | rs36184164 | G | T | G | T | 0.0356 | 0.130451 | 0.1398 | 0.0908197 | 0.125124 | 0.0850614 | 6.46E-12 | 0.005182616 | 47.18477984 | 0.000304815 |
| m6enaM | FERRITIN | iLY8vs | SCLC | rs370631 | C | T | C | T | -0.0257 | 0.028988 | 0.2558 | 0.199263 | 0.634338 | 0.0609466 | 3.27E-11 | 0.003874059 | 44.00821824 | 0.00025147 |
| m6enaM | FERRITIN | iLY8vs | SCLC | rs3738182 | A | G | A | G | -0.0233 | 0.0473007 | 0.1824 | 0.240891 | 0.410377 | 0.0574575 | 1.35E-07 | 0.004419638 | 27.79319305 | 0.000161923 |
| m6enaM | FERRITIN | iLY8vs | SCLC | rs3765602 | A | C | A | C | -0.0239 | 0.0167119 | 0.1921 | 0.090653 | 0.843022 | 0.084391 | 9.00E-08 | 0.004470768 | 28.57798042 | 0.000177301 |
| m6enaM | FERRITIN | iLY8vs | SCLC | rs3791778 | C | T | C | T | -0.029 | 0.018345 | 0.0862 | 0.100584 | 0.81994 | 0.0805939 | 5.73E-06 | 0.006393136 | 20.57633914 | 0.00013249 |
| m6enaM | FERRITIN | iLY8vs | SCLC | rs4333182 | G | A | G | A | -0.0185 | -0.0604629 | 0.3548 | 0.299231 | 0.258086 | 0.0534631 | 4.58E-07 | 0.003668363 | 25.4330736 | 0.000156694 |
| m6enaM | FERRITIN | iLY8vs | SCLC | rs4433065 | A | G | A | G | -0.0159 | 0.00339799 | 0.4618 | 0.57496 | 0.945235 | 0.0494676 | 4.81E-06 | 0.003476999 | 20.91149667 | 0.000125667 |
| m6enaM | FERRITIN | iLY8vs | SCLC | rs4492136 | T | C | T | C | -0.0303 | -0.0852478 | 0.0612 | 0.0736844 | 0.371595 | 0.0954099 | 9.71E-06 | 0.006849729 | 19.56764127 | 0.000105497 |
| m6enaM | FERRITIN | iLY8vs | SCLC | rs45520632 | C | T | C | T | 0.1061 | 0.163641 | 0.0491 | 0.022254 | 0.328486 | 0.167464 | 2.39E-39 | 0.008084244 | 172.2471092 | 0.00105118 |
| m6enaM | FERRITIN | iLY8vs | SCLC | rs4655583 | A | C | A | C | 0.0198 | 0.046354 | 0.2161 | 0.334021 | 0.371384 | 0.0518569 | 2.87E-06 | 0.00423085 | 21.90156375 | 0.000132824 |
| m6enaM | FERRITIN | iLY8vs | SCLC | rs4681485 | C | T | C | T | -0.0175 | 0.0425855 | 0.3448 | 0.391909 | 0.39739 | 0.0503202 | 1.76E-06 | 0.003661706 | 22.84068656 | 0.000138372 |
| m6enaM | FERRITIN | iLY8vs | SCLC | rs4789111 | T | C | T | C | 0.0343 | -0.0706789 | 0.1454 | 0.118317 | 0.345886 | 0.0749831 | 1.33E-12 | 0.004837014 | 50.28443229 | 0.000292379 |
| m6enaM | FERRITIN | iLY8vs | SCLC | rs4793897 | T | A | T | A | 0.0155 | -0.0999928 | 0.4137 | 0.372225 | 0.048492 | 0.0506797 | 9.18E-06 | 0.003494424 | 19.67488477 | 0.000116546 |
| m6enaM | FERRITIN | iLY8vs | SCLC | rs4808802 | C | G | C | G | 0.0275 | 0.0699394 | 0.2303 | 0.218706 | 0.236433 | 0.059073 | 3.42E-11 | 0.004149535 | 43.92042394 | 0.000268109 |
| m6enaM | FERRITIN | iLY8vs | SCLC | rs4841429 | G | A | G | A | 0.0601 | 0.132699 | 0.0791 | 0.0875627 | 0.121375 | 0.0856657 | 8.21E-21 | 0.006423058 | 87.55183575 | 0.000526221 |
| m6enaM | FERRITIN | iLY8vs | SCLC | rs4910115 | A | T | A | T | -0.0183 | 0.0833799 | 0.465 | 0.447428 | 0.0893038 | 0.0490737 | 9.94E-08 | 0.003434802 | 28.38564488 | 0.000166625 |
| m6enaM | FERRITIN | iLY8vs | SCLC | rs4938939 | A | G | A | G | 0.0224 | -0.0747645 | 0.2915 | 0.226568 | 0.195094 | 0.057704 | 3.01E-09 | 0.003776733 | 35.17737841 | 0.000207255 |
| m6enaM | FERRITIN | iLY8vs | SCLC | rs55778511 | G | T | G | T | -0.0527 | -0.0745322 | 0.0584 | 0.0743262 | 0.424815 | 0.0933876 | 3.73E-12 | 0.007585952 | 48.26152597 | 0.000305443 |
| m6enaM | FERRITIN | iLY8vs | SCLC | rs56206139 | C | A | C | A | 0.0455 | 0.100926 | 0.0788 | 0.0343125 | 0.457244 | 0.135764 | 4.07E-12 | 0.006561179 | 48.09046993 | 0.000300561 |
| m6enaM | FERRITIN | iLY8vs | SCLC | rs56343080 | A | G | A | G | -0.0216 | 0.0660803 | 0.181 | 0.170778 | 0.309952 | 0.065083 | 1.78E-06 | 0.004521741 | 22.81897521 | 0.000138325 |
| m6enaM | FERRITIN | iLY8vs | SCLC | rs590097 | T | G | T | G | -0.0198 | 0.0556202 | 0.3409 | 0.38664 | 0.268056 | 0.050219 | 4.93E-08 | 0.003630485 | 29.74411743 | 0.000176173 |
| m6enaM | FERRITIN | iLY8vs | SCLC | rs6005258 | A | T | A | T | 0.0189 | 0.066285 | 0.2096 | 0.175014 | 0.30405 | 0.0644929 | 9.82E-06 | 0.004274955 | 19.54611787 | 0.000118356 |
| m6enaM | FERRITIN | iLY8vs | SCLC | rs6059696 | G | C | G | C | -0.0374 | -0.0129511 | 0.1683 | 0.206457 | 0.830606 | 0.0605404 | 1.39E-15 | 0.004682994 | 63.78168696 | 0.000391583 |
| m6enaM | FERRITIN | iLY8vs | SCLC | rs60796549 | C | T | C | T | 0.0216 | 0.0801885 | 0.1647 | 0.12574 | 0.282749 | 0.074652 | 9.38E-06 | 0.004874753 | 19.63370547 | 0.000128373 |
| m6enaM | FERRITIN | iLY8vs | SCLC | rs6089946 | G | A | G | A | 0.0205 | -0.0231722 | 0.2569 | 0.182662 | 0.716899 | 0.0639046 | 5.79E-07 | 0.004101564 | 24.98093774 | 0.000160453 |
| m6enaM | FERRITIN | iLY8vs | SCLC | rs61160702 | G | A | G | A | 0.0261 | -0.0588124 | 0.1146 | 0.134605 | 0.412152 | 0.0717126 | 1.03E-06 | 0.005341991 | 23.87122524 | 0.00013824 |
| m6enaM | FERRITIN | iLY8vs | SCLC | rs61405481 | G | A | G | A | 0.0162 | -0.0378531 | 0.4444 | 0.490825 | 0.442834 | 0.0493253 | 3.43E-06 | 0.003488949 | 21.55960436 | 0.000129597 |
| m6enaM | FERRITIN | iLY8vs | SCLC | rs62074125 | C | A | C | A | 0.0212 | -0.0318376 | 0.2904 | 0.241862 | 0.57869 | 0.0573342 | 4.79E-08 | 0.003883542 | 29.79996192 | 0.00018523 |
| m6enaM | FERRITIN | iLY8vs | SCLC | rs62337886 | A | G | A | G | 0.0228 | 0.030087 | 0.1541 | 0.18119 | 0.63717 | 0.0637896 | 9.21E-07 | 0.004645659 | 24.08657428 | 0.000135526 |
| m6enaM | FERRITIN | iLY8vs | SCLC | rs653060 | T | C | T | C | 0.0176 | 0.0399483 | 0.4448 | 0.454507 | 0.416825 | 0.049201 | 4.18E-07 | 0.003477868 | 25.60938483 | 0.000152992 |
| m6enaM | FERRITIN | iLY8vs | SCLC | rs6597 | G | T | G | T | 0.0222 | 0.0173145 | 0.1772 | 0.111597 | 0.826587 | 0.0790325 | 1.78E-06 | 0.004647345 | 22.81897439 | 0.000143712 |
| m6enaM | FERRITIN | iLY8vs | SCLC | rs672496 | A | G | A | G | 0.0239 | -0.0137671 | 0.1538 | 0.132763 | 0.849791 | 0.0726939 | 1.16E-06 | 0.004915318 | 23.64245449 | 0.000148681 |
| m6enaM | FERRITIN | iLY8vs | SCLC | rs6750720 | G | C | G | C | -0.017 | 0.032118 | 0.317 | 0.384039 | 0.523635 | 0.0503613 | 3.53E-06 | 0.003665931 | 21.50449607 | 0.000125143 |
| m6enaM | FERRITIN | iLY8vs | SCLC | rs6760824 | A | C | A | C | 0.0316 | -0.00884261 | 0.2436 | 0.245455 | 0.875302 | 0.0563483 | 8.53E-16 | 0.003927247 | 64.74373235 | 0.000367987 |
| m6enaM | FERRITIN | iLY8vs | SCLC | rs6822746 | A | G | A | G | -0.0214 | 0.0402447 | 0.3134 | 0.382304 | 0.422607 | 0.0501864 | 5.51E-09 | 0.003670047 | 34.000421 | 0.000197088 |
| m6enaM | FERRITIN | iLY8vs | SCLC | rs7009799 | T | C | T | C | -0.0172 | -0.0502755 | 0.3641 | 0.420258 | 0.310088 | 0.0495307 | 1.63E-06 | 0.003587369 | 22.98819074 | 0.000136992 |
| m6enaM | FERRITIN | iLY8vs | SCLC | rs704017 | A | G | A | G | 0.018 | -0.0223058 | 0.3742 | 0.331294 | 0.666552 | 0.0517674 | 3.09E-07 | 0.003517086 | 26.19262556 | 0.000151745 |
| m6enaM | FERRITIN | iLY8vs | SCLC | rs7042119 | T | C | T | C | 0.0194 | -0.0378806 | 0.2238 | 0.236788 | 0.510008 | 0.0574971 | 1.89E-06 | 0.004071487 | 22.7037374 | 0.000130758 |
| m6enaM | FERRITIN | iLY8vs | SCLC | rs7068127 | G | A | G | A | 0.0197 | 0.0591374 | 0.4108 | 0.375646 | 0.242096 | 0.050555 | 1.69E-08 | 0.003492247 | 31.82163914 | 0.000187869 |
| m6enaM | FERRITIN | iLY8vs | SCLC | rs708686 | T | C | T | C | -0.0307 | 0.0101459 | 0.2304 | 0.334747 | 0.845802 | 0.0521702 | 1.96E-14 | 0.004011381 | 58.57184774 | 0.000334237 |
| m6enaM | FERRITIN | iLY8vs | SCLC | rs71537957 | T | C | T | C | 0.0228 | -0.0260203 | 0.2676 | 0.334217 | 0.615034 | 0.0517403 | 5.05E-09 | 0.003900425 | 34.17006706 | 0.000203767 |
| m6enaM | FERRITIN | iLY8vs | SCLC | rs71649623 | A | C | A | C | -0.0725 | -0.0277142 | 0.0147 | 0.0127063 | 0.899102 | 0.218573 | 2.29E-07 | 0.014012072 | 26.77141292 | 0.000152262 |
| m6enaM | FERRITIN | iLY8vs | SCLC | rs72494581 | C | T | C | T | 0.0175 | 0.0194508 | 0.3179 | 0.343432 | 0.705373 | 0.0514468 | 2.83E-06 | 0.00373709 | 21.92850293 | 0.000132814 |
| m6enaM | FERRITIN | iLY8vs | SCLC | rs72606621 | A | G | A | G | 0.0214 | 0.00186732 | 0.2642 | 0.338332 | 0.971278 | 0.0518619 | 3.73E-08 | 0.00388866 | 30.28500161 | 0.000178053 |
| m6enaM | FERRITIN | iLY8vs | SCLC | rs72775768 | T | C | T | C | -0.0202 | -0.0107542 | 0.2561 | 0.253447 | 0.848016 | 0.0561137 | 1.99E-07 | 0.003884414 | 27.04280381 | 0.000155474 |
| m6enaM | FERRITIN | iLY8vs | SCLC | rs72798422 | C | T | C | T | 0.0466 | 0.00998152 | 0.0283 | 0.0347479 | 0.939829 | 0.132233 | 2.91E-06 | 0.009963498 | 21.87500482 | 0.000119432 |
| m6enaM | FERRITIN | iLY8vs | SCLC | rs72830456 | A | G | A | G | 0.0355 | 0.23293 | 0.0599 | 0.0582528 | 0.0241964 | 0.103341 | 5.85E-07 | 0.007105536 | 24.96105963 | 0.000141934 |
| m6enaM | FERRITIN | iLY8vs | SCLC | rs73034900 | C | T | C | T | 0.0179 | -0.0462421 | 0.3922 | 0.322211 | 0.380383 | 0.052716 | 7.15E-07 | 0.003610877 | 24.57427263 | 0.000152758 |
| m6enaM | FERRITIN | iLY8vs | SCLC | rs730953 | A | C | A | C | -0.0163 | 0.0138507 | 0.3714 | 0.419024 | 0.779153 | 0.0493923 | 4.60E-06 | 0.003557205 | 20.99700834 | 0.000124057 |
| m6enaM | FERRITIN | iLY8vs | SCLC | rs735831 | G | T | G | T | 0.0486 | 0.0131511 | 0.0535 | 0.0309109 | 0.925964 | 0.141525 | 2.64E-10 | 0.007691618 | 39.9242829 | 0.000239209 |
| m6enaM | FERRITIN | iLY8vs | SCLC | rs7358798 | A | C | A | C | 0.0178 | 0.111044 | 0.3028 | 0.380267 | 0.027034 | 0.0502228 | 5.29E-06 | 0.003909557 | 20.72931284 | 0.000133778 |
| m6enaM | FERRITIN | iLY8vs | SCLC | rs7435318 | G | A | G | A | 0.0161 | 0.0249629 | 0.3703 | 0.281234 | 0.644193 | 0.0540505 | 7.95E-06 | 0.003604591 | 19.94985594 | 0.000120884 |
| m6enaM | FERRITIN | iLY8vs | SCLC | rs7478136 | G | C | G | C | 0.0202 | -0.037487 | 0.2685 | 0.276141 | 0.491425 | 0.0544835 | 1.78E-07 | 0.003869022 | 27.25839876 | 0.000160284 |
| m6enaM | FERRITIN | iLY8vs | SCLC | rs7503385 | T | G | T | G | 0.0188 | -0.042721 | 0.4315 | 0.544023 | 0.38527 | 0.0492049 | 6.10E-08 | 0.003471296 | 29.33137368 | 0.000173403 |
| m6enaM | FERRITIN | iLY8vs | SCLC | rs75848318 | G | T | G | T | 0.0463 | -0.119976 | 0.0355 | 0.0158586 | 0.54093 | 0.19623 | 6.95E-06 | 0.010299843 | 20.20695026 | 0.000146799 |
| m6enaM | FERRITIN | iLY8vs | SCLC | rs75939744 | A | G | A | G | 0.0286 | 0.0311029 | 0.0823 | 0.141734 | 0.653294 | 0.0692422 | 3.02E-06 | 0.006124912 | 21.80380853 | 0.000123556 |
| m6enaM | FERRITIN | iLY8vs | SCLC | rs7596205 | A | G | A | G | 0.0843 | 0.0777309 | 0.1021 | 0.0969466 | 0.345623 | 0.0824195 | 5.62E-49 | 0.005731075 | 216.3629855 | 0.001302983 |
| m6enaM | FERRITIN | iLY8vs | SCLC | rs75965181 | A | T | A | T | -0.1191 | -0.157364 | 0.0283 | 0.0122702 | 0.47635 | 0.220959 | 3.70E-26 | 0.01125737 | 111.9308088 | 0.000780139 |
| m6enaM | FERRITIN | iLY8vs | SCLC | rs77178111 | T | C | T | C | 0.0232 | 0.0512314 | 0.1672 | 0.118488 | 0.498372 | 0.0756682 | 4.90E-07 | 0.004612154 | 25.3027876 | 0.000149894 |
| m6enaM | FERRITIN | iLY8vs | SCLC | rs7719875 | G | A | G | A | -0.0174 | 0.00303675 | 0.4187 | 0.376945 | 0.952145 | 0.0506007 | 8.60E-07 | 0.003535698 | 24.21855185 | 0.000147378 |
| m6enaM | FERRITIN | iLY8vs | SCLC | rs77805826 | T | C | T | C | 0.0249 | 0.047087 | 0.1563 | 0.0999284 | 0.557582 | 0.0802933 | 3.82E-07 | 0.00490378 | 25.78319529 | 0.000163522 |
| m6enaM | FERRITIN | iLY8vs | SCLC | rs78180894 | C | G | C | G | 0.0383 | -0.114575 | 0.0571 | 0.0179876 | 0.544491 | 0.189057 | 9.54E-08 | 0.007178637 | 28.46515558 | 0.000157954 |
| m6enaM | FERRITIN | iLY8vs | SCLC | rs7865362 | T | C | T | C | 0.0246 | 0.0221654 | 0.3458 | 0.329477 | 0.670907 | 0.0521656 | 1.03E-11 | 0.003616452 | 46.27056512 | 0.000273801 |
| m6enaM | FERRITIN | iLY8vs | SCLC | rs79092958 | C | T | C | T | 0.0407 | 0.0942041 | 0.0451 | 0.0398245 | 0.456372 | 0.126477 | 6.32E-07 | 0.008170766 | 24.81208569 | 0.000142677 |
| m6enaM | FERRITIN | iLY8vs | SCLC | rs79242214 | A | G | A | G | 0.0465 | -0.0331832 | 0.028 | 0.0236185 | 0.830953 | 0.15544 | 5.81E-06 | 0.010257681 | 20.54979661 | 0.000117696 |
| m6enaM | FERRITIN | iLY8vs | SCLC | rs79496834 | T | C | T | C | 0.0482 | 0.081947 | 0.0459 | 0.0286396 | 0.575625 | 0.14639 | 8.86E-08 | 0.009011576 | 28.60833463 | 0.000203484 |
| m6enaM | FERRITIN | iLY8vs | SCLC | rs7959071 | G | C | G | C | 0.0168 | 0.0027445 | 0.4522 | 0.466557 | 0.955303 | 0.0489665 | 1.20E-06 | 0.003459896 | 23.57721339 | 0.00013983 |
| m6enaM | FERRITIN | iLY8vs | SCLC | rs79787985 | T | A | T | A | 0.0279 | 0.0607395 | 0.0957 | 0.0864259 | 0.4867 | 0.0873236 | 5.82E-06 | 0.006155102 | 20.54650215 | 0.00013473 |
| m6enaM | FERRITIN | iLY8vs | SCLC | rs8075680 | T | C | T | C | -0.0163 | 0.0351156 | 0.3986 | 0.330311 | 0.497733 | 0.0517882 | 3.70E-06 | 0.003522375 | 21.41430721 | 0.000127381 |
| m6enaM | FERRITIN | iLY8vs | SCLC | rs815271 | T | C | T | C | -0.0224 | -0.0358652 | 0.1752 | 0.190043 | 0.567094 | 0.0626645 | 6.55E-07 | 0.004503189 | 24.74318983 | 0.000145014 |
| m6enaM | FERRITIN | iLY8vs | SCLC | rs8177259 | C | T | C | T | -0.0168 | 0.00802928 | 0.2701 | 0.305086 | 0.87946 | 0.0529446 | 8.39E-06 | 0.003771059 | 19.8468647 | 0.000111285 |
| m6enaM | FERRITIN | iLY8vs | SCLC | rs820037 | A | G | A | G | 0.0178 | 0.0937764 | 0.2803 | 0.364605 | 0.0650924 | 0.0508376 | 4.37E-06 | 0.003875495 | 21.09529693 | 0.000127833 |
| m6enaM | FERRITIN | iLY8vs | SCLC | rs830553 | G | T | G | T | 0.0158 | -0.0862749 | 0.46 | 0.503187 | 0.0772983 | 0.0488373 | 5.49E-06 | 0.003476244 | 20.65825668 | 0.000124021 |
| m6enaM | FERRITIN | iLY8vs | SCLC | rs838112 | T | C | T | C | 0.0167 | 0.0681359 | 0.4955 | 0.424792 | 0.168287 | 0.0494552 | 1.37E-06 | 0.003458044 | 23.32232873 | 0.000139434 |
| m6enaM | FERRITIN | iLY8vs | SCLC | rs855791 | A | G | A | G | -0.0444 | 0.0607898 | 0.406 | 0.34599 | 0.236556 | 0.0513584 | 6.14E-37 | 0.003496875 | 161.2151025 | 0.000950842 |
| m6enaM | FERRITIN | iLY8vs | SCLC | rs859788 | G | A | G | A | -0.0188 | 0.0381264 | 0.4544 | 0.473085 | 0.435609 | 0.0489033 | 4.58E-08 | 0.003438884 | 29.88688452 | 0.00017525 |
| m6enaM | FERRITIN | iLY8vs | SCLC | rs9346719 | G | A | G | A | -0.0225 | -0.0435617 | 0.174 | 0.200445 | 0.471785 | 0.0605379 | 4.73E-07 | 0.004466986 | 25.37089883 | 0.000145521 |
| m6enaM | FERRITIN | iLY8vs | SCLC | rs9512463 | T | C | T | C | 0.0247 | 0.0668083 | 0.2237 | 0.258101 | 0.236877 | 0.0564817 | 3.12E-09 | 0.004168666 | 35.10748397 | 0.000211894 |
| m6enaM | FERRITIN | iLY8vs | SCLC | rs970079 | G | A | G | A | -0.0199 | 0.0641023 | 0.4602 | 0.343779 | 0.216186 | 0.0518319 | 8.31E-09 | 0.003453634 | 33.20118086 | 0.00019675 |
| m6enaM | FERRITIN | iLY8vs | SCLC | rs9921222 | C | T | C | T | 0.0248 | -0.037975 | 0.4287 | 0.522523 | 0.438212 | 0.0489864 | 1.09E-12 | 0.003483813 | 50.67499224 | 0.000301267 |
| m6enaM | FERRITIN | iLY8vs | SCLC | rs996347 | C | T | C | T | 0.0486 | -0.101925 | 0.3408 | 0.344177 | 0.0466241 | 0.0512261 | 2.99E-41 | 0.003612802 | 180.960679 | 0.001061254 |

| **Supplementary Table 3 Statistical characteristics of eligible instrumental variables in forward MR analysis.** | | | | | | | | | | | | | | | | | | |
| --- | --- | --- | --- | --- | --- | --- | --- | --- | --- | --- | --- | --- | --- | --- | --- | --- | --- | --- |
| **SERUM AND SCLC** | | | | | | | | | | | | | | | | | | |
| **id.exposure** | **exposure** | **id.outcome** | **outcome** | **SNP** | **effect_allele.exposure** | **other_allele.exposure** | **effect_allele.outcome** | **other_allele.outcome** | **beta.exposure** | **beta.outcome** | **eaf.exposure** | **eaf.outcome** | **pval.outcome** | **se.outcome** | **pval.exposure** | **se.exposure** | **F** | **R2** |
| IJ0ZJz | SERUM | m6enaM | SCLC | rs10027255 | T | C | T | C | -0.0197 | -0.0293224 | 0.3055 | 0.356503 | 0.566109 | 0.0511028 | 8.17E-06 | 4.42E-03 | 1.99E+01 | 1.65E-04 |
| IJ0ZJz | SERUM | m6enaM | SCLC | rs10138634 | G | A | G | A | 0.0187 | 0.027644 | 0.3719 | 0.305219 | 0.599461 | 0.0526376 | 6.31E-06 | 4.14E-03 | 2.04E+01 | 1.63E-04 |
| IJ0ZJz | SERUM | m6enaM | SCLC | rs10159312 | T | C | T | C | -0.0258 | 0.0142016 | 0.1573 | 0.0846112 | 0.868999 | 0.0861064 | 3.48E-06 | 5.56E-03 | 2.15E+01 | 1.76E-04 |
| IJ0ZJz | SERUM | m6enaM | SCLC | rs10261327 | T | C | T | C | 0.0189 | 0.0261776 | 0.437 | 0.387187 | 0.603644 | 0.0504223 | 2.92E-06 | 4.04E-03 | 2.19E+01 | 1.76E-04 |
| IJ0ZJz | SERUM | m6enaM | SCLC | rs10421599 | A | G | A | G | -0.0295 | 0.0770894 | 0.2051 | 0.219035 | 0.192717 | 0.059182 | 1.46E-09 | 4.88E-03 | 3.66E+01 | 2.84E-04 |
| IJ0ZJz | SERUM | m6enaM | SCLC | rs10822143 | C | T | C | T | -0.0235 | 0.0650812 | 0.4548 | 0.472783 | 0.184336 | 0.0490245 | 4.62E-09 | 4.01E-03 | 3.43E+01 | 2.74E-04 |
| IJ0ZJz | SERUM | m6enaM | SCLC | rs10824743 | C | A | C | A | -0.0206 | 0.0433655 | 0.247 | 0.208973 | 0.469914 | 0.0600115 | 9.36E-06 | 4.65E-03 | 1.96E+01 | 1.58E-04 |
| IJ0ZJz | SERUM | m6enaM | SCLC | rs10922469 | A | G | A | G | 0.0468 | 0.21038 | 0.0419 | 0.0361566 | 0.104916 | 0.129746 | 6.75E-06 | 1.04E-02 | 2.03E+01 | 1.76E-04 |
| IJ0ZJz | SERUM | m6enaM | SCLC | rs114081780 | G | A | G | A | -0.0559 | 0.0184669 | 0.0252 | 0.0138291 | 0.930999 | 0.213274 | 3.15E-06 | 1.20E-02 | 2.17E+01 | 1.54E-04 |
| IJ0ZJz | SERUM | m6enaM | SCLC | rs114165349 | C | G | C | G | 0.0721 | -0.128305 | 0.0133 | 0.037681 | 0.331937 | 0.132243 | 4.83E-06 | 1.58E-02 | 2.09E+01 | 1.36E-04 |
| IJ0ZJz | SERUM | m6enaM | SCLC | rs114708114 | T | C | T | C | 0.1412 | 0.304951 | 0.0282 | 0.00399959 | 0.437554 | 0.392812 | 7.52E-32 | 1.20E-02 | 1.38E+02 | 1.09E-03 |
| IJ0ZJz | SERUM | m6enaM | SCLC | rs114786620 | T | C | T | C | -0.0608 | -0.00395508 | 0.0372 | 0.0307248 | 0.978392 | 0.146022 | 2.46E-07 | 1.18E-02 | 2.66E+01 | 2.65E-04 |
| IJ0ZJz | SERUM | m6enaM | SCLC | rs115083160 | A | G | A | G | -0.0586 | -0.0871328 | 0.0205 | 0.0454223 | 0.45779 | 0.117352 | 5.56E-06 | 1.29E-02 | 2.06E+01 | 1.38E-04 |
| IJ0ZJz | SERUM | m6enaM | SCLC | rs116009877 | A | G | A | G | 0.2599 | -0.0645675 | 0.0647 | 0.0355846 | 0.626295 | 0.132596 | 1.00E-200 | 7.88E-03 | 1.09E+03 | 8.18E-03 |
| IJ0ZJz | SERUM | m6enaM | SCLC | rs116169498 | G | C | G | C | 0.1592 | 0.274796 | 0.0144 | 0.0117544 | 0.222601 | 0.22531 | 6.24E-21 | 1.70E-02 | 8.81E+01 | 7.19E-04 |
| IJ0ZJz | SERUM | m6enaM | SCLC | rs116583876 | A | G | A | G | -0.0374 | -0.0100625 | 0.0619 | 0.0280083 | 0.945091 | 0.146103 | 6.52E-06 | 8.29E-03 | 2.03E+01 | 1.62E-04 |
| IJ0ZJz | SERUM | m6enaM | SCLC | rs117084494 | A | G | A | G | -0.0859 | -0.159661 | 0.0106 | 0.00839749 | 0.556399 | 0.27144 | 8.24E-06 | 1.93E-02 | 1.99E+01 | 1.55E-04 |
| IJ0ZJz | SERUM | m6enaM | SCLC | rs117157488 | A | G | A | G | -0.0429 | -0.19864 | 0.0453 | 0.0280461 | 0.167571 | 0.143937 | 5.46E-06 | 9.44E-03 | 2.07E+01 | 1.59E-04 |
| IJ0ZJz | SERUM | m6enaM | SCLC | rs117718169 | T | C | T | C | -0.0718 | -0.154217 | 0.0326 | 0.0190148 | 0.390107 | 0.179442 | 3.28E-11 | 1.08E-02 | 4.40E+01 | 3.25E-04 |
| IJ0ZJz | SERUM | m6enaM | SCLC | rs117753190 | G | C | G | C | 0.0727 | 0.00097025 | 0.0283 | 0.0105393 | 0.996767 | 0.239461 | 3.37E-10 | 1.16E-02 | 3.94E+01 | 2.91E-04 |
| IJ0ZJz | SERUM | m6enaM | SCLC | rs12034310 | G | A | G | A | 0.0179 | 0.120064 | 0.4938 | 0.541436 | 0.0138455 | 0.0487818 | 7.60E-06 | 4.00E-03 | 2.00E+01 | 1.60E-04 |
| IJ0ZJz | SERUM | m6enaM | SCLC | rs12143966 | A | G | A | G | -0.0205 | 0.0327134 | 0.3796 | 0.391106 | 0.513944 | 0.0501194 | 6.35E-07 | 4.12E-03 | 2.48E+01 | 1.98E-04 |
| IJ0ZJz | SERUM | m6enaM | SCLC | rs12206204 | T | C | T | C | 0.1732 | 0.225538 | 0.0107 | 0.0157298 | 0.225544 | 0.1861 | 2.94E-22 | 1.79E-02 | 9.41E+01 | 6.35E-04 |
| IJ0ZJz | SERUM | m6enaM | SCLC | rs1223763 | T | G | T | G | 0.0234 | 0.107108 | 0.2304 | 0.199067 | 0.0792027 | 0.0610187 | 1.74E-06 | 4.89E-03 | 2.29E+01 | 1.94E-04 |
| IJ0ZJz | SERUM | m6enaM | SCLC | rs12407066 | A | C | A | C | -0.0226 | 0.0297247 | 0.2551 | 0.281146 | 0.585297 | 0.0544744 | 8.95E-07 | 4.60E-03 | 2.41E+01 | 1.94E-04 |
| IJ0ZJz | SERUM | m6enaM | SCLC | rs12633819 | G | A | G | A | 0.0258 | 0.0805215 | 0.3879 | 0.276959 | 0.14045 | 0.0546236 | 5.34E-10 | 4.16E-03 | 3.85E+01 | 3.16E-04 |
| IJ0ZJz | SERUM | m6enaM | SCLC | rs12718598 | C | T | C | T | 0.0266 | -0.0444878 | 0.4286 | 0.398309 | 0.372387 | 0.0498737 | 3.69E-11 | 4.02E-03 | 4.38E+01 | 3.47E-04 |
| IJ0ZJz | SERUM | m6enaM | SCLC | rs12975762 | G | A | G | A | -0.0289 | -0.0374246 | 0.328 | 0.232208 | 0.520045 | 0.0581782 | 1.70E-11 | 4.29E-03 | 4.53E+01 | 3.68E-04 |
| IJ0ZJz | SERUM | m6enaM | SCLC | rs13007705 | T | C | T | C | 0.0289 | -0.00214102 | 0.3745 | 0.383262 | 0.966087 | 0.0503569 | 2.01E-12 | 4.11E-03 | 4.95E+01 | 3.91E-04 |
| IJ0ZJz | SERUM | m6enaM | SCLC | rs13008165 | T | A | T | A | 0.0192 | 0.0861633 | 0.3223 | 0.245261 | 0.127566 | 0.0565461 | 6.57E-06 | 4.26E-03 | 2.03E+01 | 1.61E-04 |
| IJ0ZJz | SERUM | m6enaM | SCLC | rs13209546 | C | T | C | T | -0.0626 | 0.112658 | 0.0192 | 0.0118386 | 0.59727 | 0.213234 | 7.24E-06 | 1.40E-02 | 2.01E+01 | 1.48E-04 |
| IJ0ZJz | SERUM | m6enaM | SCLC | rs13266821 | T | C | T | C | 0.0248 | -0.0501795 | 0.1701 | 0.11561 | 0.508287 | 0.0758563 | 6.06E-06 | 5.48E-03 | 2.05E+01 | 1.74E-04 |
| IJ0ZJz | SERUM | m6enaM | SCLC | rs140047638 | C | T | C | T | 0.1002 | 0.19924 | 0.0054 | 0.00544997 | 0.529546 | 0.316909 | 4.64E-06 | 2.19E-02 | 2.10E+01 | 1.08E-04 |
| IJ0ZJz | SERUM | m6enaM | SCLC | rs140393761 | G | A | G | A | 0.1057 | 0.0782903 | 0.0199 | 0.0117317 | 0.726604 | 0.223912 | 3.34E-13 | 1.45E-02 | 5.30E+01 | 4.36E-04 |
| IJ0ZJz | SERUM | m6enaM | SCLC | rs144063236 | T | C | T | C | 0.1032 | 0.149712 | 0.006 | 0.0423414 | 0.229095 | 0.12448 | 6.03E-06 | 2.28E-02 | 2.05E+01 | 1.27E-04 |
| IJ0ZJz | SERUM | m6enaM | SCLC | rs145096438 | C | G | C | G | -0.0754 | 0.276207 | 0.0135 | 0.0235232 | 0.0964273 | 0.166147 | 5.51E-06 | 1.66E-02 | 2.07E+01 | 1.51E-04 |
| IJ0ZJz | SERUM | m6enaM | SCLC | rs145496147 | T | C | T | C | 0.1634 | -0.0892896 | 0.0081 | 0.0205774 | 0.604323 | 0.172309 | 2.25E-15 | 2.06E-02 | 6.28E+01 | 4.29E-04 |
| IJ0ZJz | SERUM | m6enaM | SCLC | rs146284844 | A | G | A | G | 0.0545 | 0.362295 | 0.03 | 0.00751146 | 0.192509 | 0.278006 | 7.85E-06 | 1.22E-02 | 2.00E+01 | 1.73E-04 |
| IJ0ZJz | SERUM | m6enaM | SCLC | rs148978261 | G | C | G | C | 0.0813 | 0.842177 | 0.0153 | 0.00332093 | 0.0414763 | 0.413088 | 6.69E-06 | 1.81E-02 | 2.03E+01 | 1.99E-04 |
| IJ0ZJz | SERUM | m6enaM | SCLC | rs149830227 | T | C | T | C | 0.0566 | 0.00598976 | 0.028 | 0.00839627 | 0.982967 | 0.280555 | 8.21E-06 | 1.27E-02 | 1.99E+01 | 1.74E-04 |
| IJ0ZJz | SERUM | m6enaM | SCLC | rs16871037 | G | C | G | C | 0.0746 | -0.08251 | 0.0151 | 0.00281283 | 0.848567 | 0.432109 | 1.17E-06 | 1.53E-02 | 2.36E+01 | 1.66E-04 |
| IJ0ZJz | SERUM | m6enaM | SCLC | rs17019886 | G | T | G | T | 0.0497 | 0.0409629 | 0.0348 | 0.0294083 | 0.775581 | 0.143688 | 4.39E-06 | 1.08E-02 | 2.11E+01 | 1.66E-04 |
| IJ0ZJz | SERUM | m6enaM | SCLC | rs17767386 | C | T | C | T | -0.0219 | 0.0485071 | 0.2159 | 0.173638 | 0.45171 | 0.0644554 | 5.29E-06 | 4.81E-03 | 2.07E+01 | 1.62E-04 |
| IJ0ZJz | SERUM | m6enaM | SCLC | rs1958078 | A | C | A | C | -0.0321 | 0.0728771 | 0.133 | 0.080597 | 0.421038 | 0.0905732 | 2.48E-08 | 5.76E-03 | 3.11E+01 | 2.38E-04 |
| IJ0ZJz | SERUM | m6enaM | SCLC | rs2042919 | G | A | G | A | -0.0225 | 0.122413 | 0.2494 | 0.207613 | 0.0416342 | 0.0600902 | 1.01E-06 | 4.60E-03 | 2.39E+01 | 1.90E-04 |
| IJ0ZJz | SERUM | m6enaM | SCLC | rs2043192 | G | A | G | A | -0.0192 | 0.0357087 | 0.4445 | 0.448337 | 0.466579 | 0.0490466 | 2.58E-06 | 4.08E-03 | 2.21E+01 | 1.82E-04 |
| IJ0ZJz | SERUM | m6enaM | SCLC | rs2158799 | C | G | C | G | -0.0211 | 0.0641428 | 0.403 | 0.456642 | 0.193185 | 0.0492946 | 2.73E-07 | 4.10E-03 | 2.64E+01 | 2.14E-04 |
| IJ0ZJz | SERUM | m6enaM | SCLC | rs2613522 | G | A | G | A | -0.0212 | -0.0464105 | 0.2834 | 0.266005 | 0.402105 | 0.0553912 | 2.09E-06 | 4.47E-03 | 2.25E+01 | 1.83E-04 |
| IJ0ZJz | SERUM | m6enaM | SCLC | rs28929474 | T | C | T | C | 0.0922 | 0.273469 | 0.0082 | 0.0197877 | 0.125654 | 0.178567 | 3.66E-07 | 1.81E-02 | 2.59E+01 | 1.38E-04 |
| IJ0ZJz | SERUM | m6enaM | SCLC | rs41272158 | T | C | T | C | 0.04 | 0.035274 | 0.0675 | 0.0680187 | 0.726246 | 0.100747 | 4.31E-07 | 7.91E-03 | 2.56E+01 | 2.01E-04 |
| IJ0ZJz | SERUM | m6enaM | SCLC | rs4774514 | T | C | T | C | -0.0491 | 0.00632029 | 0.0665 | 0.0390256 | 0.960786 | 0.128546 | 2.87E-09 | 8.27E-03 | 3.53E+01 | 2.99E-04 |
| IJ0ZJz | SERUM | m6enaM | SCLC | rs4817984 | A | C | A | C | 0.0213 | -0.0236753 | 0.3116 | 0.260274 | 0.671508 | 0.0558275 | 1.19E-06 | 4.39E-03 | 2.36E+01 | 1.95E-04 |
| IJ0ZJz | SERUM | m6enaM | SCLC | rs4854760 | G | A | G | A | 0.053 | 0.00793184 | 0.2639 | 0.295787 | 0.882086 | 0.053476 | 5.67E-33 | 4.43E-03 | 1.43E+02 | 1.09E-03 |
| IJ0ZJz | SERUM | m6enaM | SCLC | rs55709272 | C | T | C | T | -0.0202 | 0.0432399 | 0.489 | 0.322249 | 0.410703 | 0.0525613 | 4.16E-07 | 3.99E-03 | 2.56E+01 | 2.04E-04 |
| IJ0ZJz | SERUM | m6enaM | SCLC | rs563027675 | G | C | G | C | -0.0718 | -0.0287076 | 0.0257 | 0.0303506 | 0.842065 | 0.144076 | 3.13E-08 | 1.30E-02 | 3.06E+01 | 2.58E-04 |
| IJ0ZJz | SERUM | m6enaM | SCLC | rs563076 | C | A | C | A | 0.0189 | 0.0135836 | 0.381 | 0.464539 | 0.781345 | 0.048938 | 4.16E-06 | 4.11E-03 | 2.12E+01 | 1.68E-04 |
| IJ0ZJz | SERUM | m6enaM | SCLC | rs56912861 | A | G | A | G | -0.0337 | 0.0244596 | 0.3658 | 0.470217 | 0.618408 | 0.0491049 | 1.02E-15 | 4.20E-03 | 6.44E+01 | 5.27E-04 |
| IJ0ZJz | SERUM | m6enaM | SCLC | rs58658771 | A | T | A | T | 0.0246 | 0.0354767 | 0.1654 | 0.26492 | 0.52065 | 0.0552302 | 3.65E-06 | 5.31E-03 | 2.14E+01 | 1.67E-04 |
| IJ0ZJz | SERUM | m6enaM | SCLC | rs7216369 | T | C | T | C | -0.0338 | 0.0655824 | 0.0923 | 0.0438915 | 0.579154 | 0.118247 | 1.88E-06 | 7.09E-03 | 2.27E+01 | 1.91E-04 |
| IJ0ZJz | SERUM | m6enaM | SCLC | rs73181000 | A | G | A | G | 0.0483 | 0.00564805 | 0.0519 | 0.0466153 | 0.961545 | 0.117142 | 8.35E-07 | 9.80E-03 | 2.43E+01 | 2.30E-04 |
| IJ0ZJz | SERUM | m6enaM | SCLC | rs7385804 | C | A | C | A | -0.0572 | -0.0129294 | 0.3516 | 0.448519 | 0.791715 | 0.0489595 | 9.42E-43 | 4.17E-03 | 1.88E+02 | 1.49E-03 |
| IJ0ZJz | SERUM | m6enaM | SCLC | rs739936 | T | C | T | C | -0.0254 | 0.00199711 | 0.2141 | 0.288979 | 0.970565 | 0.0541236 | 1.34E-07 | 4.82E-03 | 2.78E+01 | 2.17E-04 |
| IJ0ZJz | SERUM | m6enaM | SCLC | rs74293923 | C | T | C | T | -0.0635 | -0.0592492 | 0.0188 | 0.00996942 | 0.809396 | 0.245638 | 9.87E-06 | 1.44E-02 | 1.95E+01 | 1.49E-04 |
| IJ0ZJz | SERUM | m6enaM | SCLC | rs74338506 | A | G | A | G | -0.0649 | 0.412713 | 0.0201 | 0.0224054 | 0.00505627 | 0.147217 | 2.14E-06 | 1.37E-02 | 2.25E+01 | 1.66E-04 |
| IJ0ZJz | SERUM | m6enaM | SCLC | rs7574777 | C | T | C | T | 0.0266 | 0.124671 | 0.144 | 0.127678 | 0.0887769 | 0.0732544 | 7.64E-06 | 5.94E-03 | 2.00E+01 | 1.74E-04 |
| IJ0ZJz | SERUM | m6enaM | SCLC | rs77034634 | T | C | T | C | 0.0369 | -0.130186 | 0.0607 | 0.0961854 | 0.120757 | 0.0839045 | 4.13E-06 | 8.01E-03 | 2.12E+01 | 1.55E-04 |
| IJ0ZJz | SERUM | m6enaM | SCLC | rs77283192 | C | T | C | T | 0.0512 | -0.0347761 | 0.033 | 0.0274549 | 0.810876 | 0.145327 | 8.15E-06 | 1.15E-02 | 1.99E+01 | 1.67E-04 |
| IJ0ZJz | SERUM | m6enaM | SCLC | rs7837764 | C | G | C | G | 0.0223 | -0.065396 | 0.4021 | 0.378357 | 0.193661 | 0.0503113 | 4.98E-08 | 4.09E-03 | 2.97E+01 | 2.39E-04 |
| IJ0ZJz | SERUM | m6enaM | SCLC | rs9265878 | A | G | A | G | -0.0265 | -0.0664215 | 0.2404 | 0.279328 | 0.221849 | 0.0543713 | 9.33E-09 | 4.61E-03 | 3.30E+01 | 2.56E-04 |
| IJ0ZJz | SERUM | m6enaM | SCLC | rs9295767 | A | G | A | G | -0.0649 | 0.17301 | 0.0531 | 0.0316876 | 0.20619 | 0.136863 | 4.90E-12 | 9.39E-03 | 4.77E+01 | 4.24E-04 |
| IJ0ZJz | SERUM | m6enaM | SCLC | rs9399136 | C | T | C | T | 0.0574 | 0.0490675 | 0.2695 | 0.343948 | 0.341927 | 0.0516303 | 1.08E-36 | 4.54E-03 | 1.60E+02 | 1.30E-03 |
| IJ0ZJz | SERUM | m6enaM | SCLC | rs983828 | G | A | G | A | -0.0223 | 0.112246 | 0.2625 | 0.350267 | 0.0287455 | 0.0513254 | 9.56E-07 | 4.55E-03 | 2.40E+01 | 1.93E-04 |

| **Supplementary Table 4 Statistical characteristics of eligible instrumental variables in forward MR analysis.** | | | | | | | | | | | | | | | | | | |
| --- | --- | --- | --- | --- | --- | --- | --- | --- | --- | --- | --- | --- | --- | --- | --- | --- | --- | --- |
| **TIBC AND SCLC** | | | | | | | | | | | | | | | | | | |
| id.exposure | exposure | id.outcome | outcome | SNP | effect_allele.exposure | other_allele.exposure | effect_allele.outcome | other_allele.outcome | beta.exposure | beta.outcome | eaf.exposure | eaf.outcome | pval.outcome | se.outcome | pval.exposure | se.exposure | F | R2 |
| YgJTec | TIBC | ItGEB2 | SCLC | rs10045483 | G | A | G | A | -0.0755 | 0.37012 | 0.0196 | 0.00411611 | 0.336479 | 0.385082 | 4.12E-06 | 0.016394385 | 21.20819187 | 0.00021907 |
| YgJTec | TIBC | ItGEB2 | SCLC | rs1106735 | G | A | G | A | 0.0791 | 0.0505154 | 0.1078 | 0.152312 | 0.451865 | 0.0671471 | 2.92E-27 | 0.007313854 | 116.9662035 | 0.001203549 |
| YgJTec | TIBC | ItGEB2 | SCLC | rs11125072 | A | T | A | T | 0.0259 | 0.0167704 | 0.4578 | 0.456463 | 0.733562 | 0.0492681 | 3.25E-08 | 0.004685742 | 30.55222076 | 0.000333016 |
| YgJTec | TIBC | ItGEB2 | SCLC | rs111302943 | A | G | A | G | 0.0328 | 0.0293721 | 0.1258 | 0.225581 | 0.617565 | 0.0588262 | 3.54E-06 | 0.007073983 | 21.49906951 | 0.00023663 |
| YgJTec | TIBC | ItGEB2 | SCLC | rs112466891 | C | A | C | A | 0.0453 | 0.0891618 | 0.1791 | 0.24367 | 0.12008 | 0.0573595 | 1.63E-13 | 0.006141461 | 54.40677799 | 0.00060341 |
| YgJTec | TIBC | ItGEB2 | SCLC | rs112727702 | T | G | T | G | 0.0433 | 0.0172185 | 0.1963 | 0.215021 | 0.772263 | 0.0594936 | 2.08E-14 | 0.0056634 | 58.45495355 | 0.000591589 |
| YgJTec | TIBC | ItGEB2 | SCLC | rs115421711 | G | A | G | A | -0.0598 | 0.124424 | 0.0402 | 0.029657 | 0.37743 | 0.140968 | 3.66E-07 | 0.011758133 | 25.86578309 | 0.000275956 |
| YgJTec | TIBC | ItGEB2 | SCLC | rs116272812 | C | T | C | T | -0.2414 | -0.0288596 | 0.1181 | 0.128951 | 0.690691 | 0.0725268 | 1.00E-200 | 0.007032765 | 1178.208957 | 0.012138744 |
| YgJTec | TIBC | ItGEB2 | SCLC | rs118030488 | T | G | T | G | -0.0808 | -0.0825249 | 0.0253 | 0.0297424 | 0.575565 | 0.147399 | 4.08E-07 | 0.015952028 | 25.65611627 | 0.000321991 |
| YgJTec | TIBC | ItGEB2 | SCLC | rs118066080 | T | C | T | C | 0.0769 | -0.37556 | 0.0206 | 0.0174915 | 0.040679 | 0.183488 | 8.78E-06 | 0.017299462 | 19.7600248 | 0.000238622 |
| YgJTec | TIBC | ItGEB2 | SCLC | rs11918732 | T | C | T | C | 0.0223 | 0.00198262 | 0.361 | 0.355282 | 0.968995 | 0.0510078 | 4.61E-06 | 0.004867087 | 20.99285506 | 0.000229429 |
| YgJTec | TIBC | ItGEB2 | SCLC | rs1198439 | A | T | A | T | -0.0371 | 0.0253647 | 0.1232 | 0.112153 | 0.741679 | 0.0769489 | 2.14E-07 | 0.007152847 | 26.90233325 | 0.000297364 |
| YgJTec | TIBC | ItGEB2 | SCLC | rs12206077 | A | G | A | G | -0.0665 | 0.101209 | 0.2838 | 0.282633 | 0.0622673 | 0.0542854 | 2.07E-38 | 0.005131283 | 167.9543883 | 0.001797711 |
| YgJTec | TIBC | ItGEB2 | SCLC | rs12879801 | G | A | G | A | 0.0252 | 0.0810363 | 0.4676 | 0.510488 | 0.0987825 | 0.0490897 | 6.11E-08 | 0.004653265 | 29.32820678 | 0.000316187 |
| YgJTec | TIBC | ItGEB2 | SCLC | rs12976652 | C | T | C | T | -0.0446 | -0.0996439 | 0.0979 | 0.0633589 | 0.319279 | 0.10005 | 2.55E-09 | 0.007485457 | 35.50038619 | 0.000351348 |
| YgJTec | TIBC | ItGEB2 | SCLC | rs13008704 | C | T | C | T | -0.0369 | -0.0246759 | 0.4876 | 0.512609 | 0.613462 | 0.0488499 | 3.44E-15 | 0.004686422 | 61.99689599 | 0.000680386 |
| YgJTec | TIBC | ItGEB2 | SCLC | rs13084306 | A | C | A | C | 0.1043 | -0.0465054 | 0.0253 | 0.0118741 | 0.840584 | 0.231202 | 1.32E-10 | 0.016233843 | 41.27869262 | 0.000536525 |
| YgJTec | TIBC | ItGEB2 | SCLC | rs143530446 | T | C | T | C | -0.1591 | 0.280556 | 0.0299 | 0.00920302 | 0.283126 | 0.26139 | 2.96E-26 | 0.015008551 | 112.3732208 | 0.001468446 |
| YgJTec | TIBC | ItGEB2 | SCLC | rs143878994 | A | G | A | G | 0.0567 | -0.0391551 | 0.0451 | 0.0285819 | 0.7905 | 0.147387 | 8.26E-07 | 0.011503063 | 24.29624292 | 0.000276905 |
| YgJTec | TIBC | ItGEB2 | SCLC | rs1672992 | C | T | C | T | -0.033 | 0.0221061 | 0.1513 | 0.140139 | 0.752813 | 0.070193 | 5.33E-07 | 0.006581524 | 25.14055964 | 0.000279673 |
| YgJTec | TIBC | ItGEB2 | SCLC | rs1680695 | G | T | G | T | -0.0247 | -0.0954405 | 0.3473 | 0.343548 | 0.0629202 | 0.0513189 | 3.68E-07 | 0.004857608 | 25.85526398 | 0.000276594 |
| YgJTec | TIBC | ItGEB2 | SCLC | rs174547 | C | T | C | T | 0.0462 | -0.0376517 | 0.3883 | 0.414333 | 0.445755 | 0.0493785 | 6.57E-22 | 0.004802392 | 92.54836215 | 0.001013958 |
| YgJTec | TIBC | ItGEB2 | SCLC | rs17580 | A | T | A | T | 0.0762 | -0.129623 | 0.0333 | 0.00915574 | 0.611091 | 0.254905 | 1.19E-10 | 0.011831191 | 41.48136238 | 0.000373831 |
| YgJTec | TIBC | ItGEB2 | SCLC | rs17767742 | G | C | G | C | 0.0305 | -0.0384261 | 0.2828 | 0.310028 | 0.466526 | 0.0527728 | 1.95E-09 | 0.005081709 | 36.02301798 | 0.000377354 |
| YgJTec | TIBC | ItGEB2 | SCLC | rs192458696 | C | T | C | T | -0.1056 | 0.568051 | 0.0139 | 0.00301113 | 0.172948 | 0.416828 | 1.78E-07 | 0.020226173 | 27.25840258 | 0.000305699 |
| YgJTec | TIBC | ItGEB2 | SCLC | rs1927693 | A | G | A | G | 0.0367 | 0.0262961 | 0.382 | 0.369671 | 0.603544 | 0.0506366 | 2.02E-14 | 0.004797794 | 58.51253778 | 0.000635937 |
| YgJTec | TIBC | ItGEB2 | SCLC | rs1930884 | T | C | T | C | -0.0239 | 0.0350347 | 0.289 | 0.212083 | 0.565361 | 0.060941 | 7.36E-06 | 0.005331248 | 20.09731459 | 0.000234743 |
| YgJTec | TIBC | ItGEB2 | SCLC | rs199138 | A | G | A | G | 0.0807 | -0.0115439 | 0.0815 | 0.070768 | 0.904041 | 0.0957535 | 9.59E-21 | 0.008639815 | 87.24452509 | 0.000975021 |
| YgJTec | TIBC | ItGEB2 | SCLC | rs2088102 | T | C | T | C | -0.0213 | -0.0329212 | 0.4962 | 0.517011 | 0.501359 | 0.0489643 | 4.17E-06 | 0.004627694 | 21.18507172 | 0.000226832 |
| YgJTec | TIBC | ItGEB2 | SCLC | rs2236252 | T | C | T | C | 0.0359 | 0.0459945 | 0.1788 | 0.147783 | 0.501951 | 0.0685029 | 4.02E-09 | 0.006101947 | 34.61401957 | 0.000378473 |
| YgJTec | TIBC | ItGEB2 | SCLC | rs2305268 | T | C | T | C | 0.0956 | 0.129755 | 0.016 | 0.0472368 | 0.263421 | 0.116025 | 2.34E-07 | 0.018491028 | 26.72967462 | 0.00028778 |
| YgJTec | TIBC | ItGEB2 | SCLC | rs28411900 | T | C | T | C | -0.0319 | -0.0218911 | 0.1141 | 0.166606 | 0.738108 | 0.0654717 | 6.64E-06 | 0.007081157 | 20.29424594 | 0.000205722 |
| YgJTec | TIBC | ItGEB2 | SCLC | rs2875251 | C | T | C | T | -0.0257 | 0.124234 | 0.2637 | 0.247238 | 0.0275588 | 0.0563799 | 2.18E-06 | 0.005426544 | 22.42949983 | 0.000256485 |
| YgJTec | TIBC | ItGEB2 | SCLC | rs35570672 | T | C | T | C | -0.0429 | 0.0440149 | 0.1877 | 0.251006 | 0.433038 | 0.0561412 | 1.26E-13 | 0.005789228 | 54.9127605 | 0.00056121 |
| YgJTec | TIBC | ItGEB2 | SCLC | rs35769520 | G | A | G | A | 0.0264 | 0.0109256 | 0.4885 | 0.63611 | 0.829106 | 0.0506173 | 1.34E-08 | 0.004647164 | 32.27244397 | 0.000348296 |
| YgJTec | TIBC | ItGEB2 | SCLC | rs4671609 | G | A | G | A | -0.0219 | -0.0707414 | 0.3875 | 0.362292 | 0.162524 | 0.0506514 | 4.20E-06 | 0.004759595 | 21.17133433 | 0.000227665 |
| YgJTec | TIBC | ItGEB2 | SCLC | rs469882 | C | A | C | A | -0.0367 | -0.0112329 | 0.2221 | 0.21455 | 0.851727 | 0.0600959 | 1.03E-10 | 0.005678933 | 41.76366838 | 0.000465409 |
| YgJTec | TIBC | ItGEB2 | SCLC | rs4764934 | T | C | T | C | -0.0265 | -0.0209661 | 0.2013 | 0.282269 | 0.700156 | 0.0544419 | 6.90E-06 | 0.005893145 | 20.2207609 | 0.000225813 |
| YgJTec | TIBC | ItGEB2 | SCLC | rs4846335 | A | C | A | C | 0.0445 | 0.0904884 | 0.1105 | 0.144657 | 0.195083 | 0.0698381 | 2.87E-09 | 0.007493013 | 35.27012914 | 0.000389277 |
| YgJTec | TIBC | ItGEB2 | SCLC | rs4849902 | C | T | C | T | 0.0241 | -0.00693187 | 0.386 | 0.277878 | 0.899549 | 0.0549138 | 6.48E-07 | 0.004842923 | 24.76389518 | 0.000275309 |
| YgJTec | TIBC | ItGEB2 | SCLC | rs4854737 | G | C | G | C | 0.1444 | -0.0482375 | 0.4575 | 0.362928 | 0.33987 | 0.050541 | 1.00E-200 | 0.004692648 | 946.8876 | 0.010350354 |
| YgJTec | TIBC | ItGEB2 | SCLC | rs56195124 | A | G | A | G | 0.0882 | -0.0191208 | 0.0543 | 0.0321954 | 0.888466 | 0.136337 | 3.45E-16 | 0.010813521 | 66.52776205 | 0.000798951 |
| YgJTec | TIBC | ItGEB2 | SCLC | rs59950280 | A | G | A | G | 0.0328 | -0.078012 | 0.3195 | 0.242968 | 0.171853 | 0.0570984 | 5.87E-11 | 0.00500992 | 42.86334951 | 0.000467818 |
| YgJTec | TIBC | ItGEB2 | SCLC | rs6025 | T | C | T | C | -0.093 | -0.249639 | 0.0188 | 0.0201998 | 0.158164 | 0.176889 | 1.76E-08 | 0.016506701 | 31.74280695 | 0.000319089 |
| YgJTec | TIBC | ItGEB2 | SCLC | rs60721435 | C | T | C | T | 0.0372 | 0.0134329 | 0.1152 | 0.0857709 | 0.877312 | 0.087013 | 8.41E-08 | 0.006942755 | 28.70927332 | 0.000282107 |
| YgJTec | TIBC | ItGEB2 | SCLC | rs62183592 | T | C | T | C | 0.0496 | -0.113395 | 0.077 | 0.0397008 | 0.367278 | 0.125773 | 3.31E-08 | 0.008978684 | 30.51672885 | 0.000349692 |
| YgJTec | TIBC | ItGEB2 | SCLC | rs637828 | C | A | C | A | -0.0221 | -0.0409612 | 0.4011 | 0.40633 | 0.408955 | 0.0496057 | 2.76E-06 | 0.004714247 | 21.97656848 | 0.000234651 |
| YgJTec | TIBC | ItGEB2 | SCLC | rs715 | C | T | C | T | 0.0247 | -0.00293581 | 0.2726 | 0.313693 | 0.955828 | 0.0530025 | 1.54E-06 | 0.005139437 | 23.09738497 | 0.000241949 |
| YgJTec | TIBC | ItGEB2 | SCLC | rs722740 | C | T | C | T | 0.0236 | -0.00606721 | 0.3965 | 0.474954 | 0.901463 | 0.0490026 | 6.98E-07 | 0.004756224 | 24.62064385 | 0.000266547 |
| YgJTec | TIBC | ItGEB2 | SCLC | rs72840508 | T | A | T | A | -0.1212 | 0.0324469 | 0.034 | 0.0182603 | 0.85872 | 0.182283 | 3.40E-20 | 0.013166004 | 84.74171993 | 0.00096492 |
| YgJTec | TIBC | ItGEB2 | SCLC | rs7297861 | C | T | C | T | 0.0479 | -0.0236922 | 0.0794 | 0.122245 | 0.746956 | 0.0734287 | 3.99E-09 | 0.00813988 | 34.62860702 | 0.000335423 |
| YgJTec | TIBC | ItGEB2 | SCLC | rs7432894 | C | T | C | T | -0.0377 | 0.0739785 | 0.2955 | 0.272396 | 0.179877 | 0.0551612 | 1.52E-13 | 0.005104669 | 54.54406825 | 0.000591768 |
| YgJTec | TIBC | ItGEB2 | SCLC | rs7500731 | G | A | G | A | 0.0229 | 0.00618618 | 0.4891 | 0.303077 | 0.906871 | 0.0528794 | 9.45E-07 | 0.00467084 | 24.03703983 | 0.00026208 |
| YgJTec | TIBC | ItGEB2 | SCLC | rs780093 | T | C | T | C | 0.0235 | 0.0721465 | 0.3416 | 0.352396 | 0.15792 | 0.0510916 | 1.10E-06 | 0.00482264 | 23.74466445 | 0.000248412 |
| YgJTec | TIBC | ItGEB2 | SCLC | rs79364962 | A | G | A | G | 0.0364 | -0.00341346 | 0.1005 | 0.0635962 | 0.973219 | 0.101679 | 8.50E-06 | 0.008175757 | 19.82197014 | 0.000239552 |
| YgJTec | TIBC | ItGEB2 | SCLC | rs8062982 | C | G | C | G | 0.0255 | 0.0711767 | 0.3938 | 0.454514 | 0.147097 | 0.0490921 | 1.01E-07 | 0.004788808 | 28.35472965 | 0.000310457 |
| YgJTec | TIBC | ItGEB2 | SCLC | rs8177257 | T | C | T | C | -0.2878 | 0.0879017 | 0.0735 | 0.0661752 | 0.376352 | 0.0993646 | 1.39E-169 | 0.010368133 | 770.5138685 | 0.011280915 |
| YgJTec | TIBC | ItGEB2 | SCLC | rs854762 | G | A | G | A | 0.0216 | -0.0564814 | 0.3862 | 0.48629 | 0.250596 | 0.0491612 | 5.38E-06 | 0.004747883 | 20.6970041 | 0.000221196 |
| YgJTec | TIBC | ItGEB2 | SCLC | rs855791 | A | G | A | G | 0.026 | 0.0607898 | 0.406 | 0.34599 | 0.236556 | 0.0513584 | 2.88E-08 | 0.004685884 | 30.7867347 | 0.000326054 |
| YgJTec | TIBC | ItGEB2 | SCLC | rs9261848 | C | G | C | G | 0.0297 | 0.218816 | 0.1582 | 0.0859398 | 0.012096 | 0.0872008 | 5.63E-06 | 0.006542097 | 20.61004502 | 0.000234941 |
| YgJTec | TIBC | ItGEB2 | SCLC | rs9389269 | C | T | C | T | -0.0336 | 0.0405719 | 0.2792 | 0.342575 | 0.430678 | 0.0514851 | 9.10E-11 | 0.005184231 | 42.00585853 | 0.0004544 |
| YgJTec | TIBC | ItGEB2 | SCLC | rs968155 | C | T | C | T | -0.0547 | -0.00884124 | 0.4638 | 0.439937 | 0.857869 | 0.0493684 | 3.45E-31 | 0.004709348 | 134.9127779 | 0.001488203 |

| **Supplementary Table 5 Statistical characteristics of eligible instrumental variables in forward MR analysis.** | | | | | | | | | | | | | | | | | | |
| --- | --- | --- | --- | --- | --- | --- | --- | --- | --- | --- | --- | --- | --- | --- | --- | --- | --- | --- |
| **TSAT AND SCLC** | | | | | | | | | | | | | | | | | | |
| id.exposure | exposure | id.outcome | outcome | SNP | effect_allele.exposure | other_allele.exposure | effect_allele.outcome | other_allele.outcome | beta.exposure | beta.outcome | eaf.exposure | eaf.outcome | pval.outcome | se.outcome | pval.exposure | se.exposure | F | R2 |
| 25xisD | TSAT | vokvCt | SCLC | rs10045483 | G | A | G | A | -0.0755 | 0.37012 | 0.0196 | 0.00411611 | 0.336479 | 0.385082 | 4.12E-06 | 0.016394385 | 21.20819187 | 0.00021907 |
| 25xisD | TSAT | vokvCt | SCLC | rs1106735 | G | A | G | A | 0.0791 | 0.0505154 | 0.1078 | 0.152312 | 0.451865 | 0.0671471 | 2.92E-27 | 0.007313854 | 116.9662035 | 0.001203549 |
| 25xisD | TSAT | vokvCt | SCLC | rs11125072 | A | T | A | T | 0.0259 | 0.0167704 | 0.4578 | 0.456463 | 0.733562 | 0.0492681 | 3.25E-08 | 0.004685742 | 30.55222076 | 0.000333016 |
| 25xisD | TSAT | vokvCt | SCLC | rs111302943 | A | G | A | G | 0.0328 | 0.0293721 | 0.1258 | 0.225581 | 0.617565 | 0.0588262 | 3.54E-06 | 0.007073983 | 21.49906951 | 0.00023663 |
| 25xisD | TSAT | vokvCt | SCLC | rs112466891 | C | A | C | A | 0.0453 | 0.0891618 | 0.1791 | 0.24367 | 0.12008 | 0.0573595 | 1.63E-13 | 0.006141461 | 54.40677799 | 0.00060341 |
| 25xisD | TSAT | vokvCt | SCLC | rs112727702 | T | G | T | G | 0.0433 | 0.0172185 | 0.1963 | 0.215021 | 0.772263 | 0.0594936 | 2.08E-14 | 0.0056634 | 58.45495355 | 0.000591589 |
| 25xisD | TSAT | vokvCt | SCLC | rs115421711 | G | A | G | A | -0.0598 | 0.124424 | 0.0402 | 0.029657 | 0.37743 | 0.140968 | 3.66E-07 | 0.011758133 | 25.86578309 | 0.000275956 |
| 25xisD | TSAT | vokvCt | SCLC | rs116272812 | C | T | C | T | -0.2414 | -0.0288596 | 0.1181 | 0.128951 | 0.690691 | 0.0725268 | 1.00E-200 | 0.007032765 | 1178.208957 | 0.012138744 |
| 25xisD | TSAT | vokvCt | SCLC | rs118030488 | T | G | T | G | -0.0808 | -0.0825249 | 0.0253 | 0.0297424 | 0.575565 | 0.147399 | 4.08E-07 | 0.015952028 | 25.65611627 | 0.000321991 |
| 25xisD | TSAT | vokvCt | SCLC | rs118066080 | T | C | T | C | 0.0769 | -0.37556 | 0.0206 | 0.0174915 | 0.040679 | 0.183488 | 8.78E-06 | 0.017299462 | 19.7600248 | 0.000238622 |
| 25xisD | TSAT | vokvCt | SCLC | rs11918732 | T | C | T | C | 0.0223 | 0.00198262 | 0.361 | 0.355282 | 0.968995 | 0.0510078 | 4.61E-06 | 0.004867087 | 20.99285506 | 0.000229429 |
| 25xisD | TSAT | vokvCt | SCLC | rs1198439 | A | T | A | T | -0.0371 | 0.0253647 | 0.1232 | 0.112153 | 0.741679 | 0.0769489 | 2.14E-07 | 0.007152847 | 26.90233325 | 0.000297364 |
| 25xisD | TSAT | vokvCt | SCLC | rs12206077 | A | G | A | G | -0.0665 | 0.101209 | 0.2838 | 0.282633 | 0.0622673 | 0.0542854 | 2.07E-38 | 0.005131283 | 167.9543883 | 0.001797711 |
| 25xisD | TSAT | vokvCt | SCLC | rs12879801 | G | A | G | A | 0.0252 | 0.0810363 | 0.4676 | 0.510488 | 0.0987825 | 0.0490897 | 6.11E-08 | 0.004653265 | 29.32820678 | 0.000316187 |
| 25xisD | TSAT | vokvCt | SCLC | rs12976652 | C | T | C | T | -0.0446 | -0.0996439 | 0.0979 | 0.0633589 | 0.319279 | 0.10005 | 2.55E-09 | 0.007485457 | 35.50038619 | 0.000351348 |
| 25xisD | TSAT | vokvCt | SCLC | rs13008704 | C | T | C | T | -0.0369 | -0.0246759 | 0.4876 | 0.512609 | 0.613462 | 0.0488499 | 3.44E-15 | 0.004686422 | 61.99689599 | 0.000680386 |
| 25xisD | TSAT | vokvCt | SCLC | rs13084306 | A | C | A | C | 0.1043 | -0.0465054 | 0.0253 | 0.0118741 | 0.840584 | 0.231202 | 1.32E-10 | 0.016233843 | 41.27869262 | 0.000536525 |
| 25xisD | TSAT | vokvCt | SCLC | rs143530446 | T | C | T | C | -0.1591 | 0.280556 | 0.0299 | 0.00920302 | 0.283126 | 0.26139 | 2.96E-26 | 0.015008551 | 112.3732208 | 0.001468446 |
| 25xisD | TSAT | vokvCt | SCLC | rs143878994 | A | G | A | G | 0.0567 | -0.0391551 | 0.0451 | 0.0285819 | 0.7905 | 0.147387 | 8.26E-07 | 0.011503063 | 24.29624292 | 0.000276905 |
| 25xisD | TSAT | vokvCt | SCLC | rs1672992 | C | T | C | T | -0.033 | 0.0221061 | 0.1513 | 0.140139 | 0.752813 | 0.070193 | 5.33E-07 | 0.006581524 | 25.14055964 | 0.000279673 |
| 25xisD | TSAT | vokvCt | SCLC | rs1680695 | G | T | G | T | -0.0247 | -0.0954405 | 0.3473 | 0.343548 | 0.0629202 | 0.0513189 | 3.68E-07 | 0.004857608 | 25.85526398 | 0.000276594 |
| 25xisD | TSAT | vokvCt | SCLC | rs174547 | C | T | C | T | 0.0462 | -0.0376517 | 0.3883 | 0.414333 | 0.445755 | 0.0493785 | 6.57E-22 | 0.004802392 | 92.54836215 | 0.001013958 |
| 25xisD | TSAT | vokvCt | SCLC | rs17580 | A | T | A | T | 0.0762 | -0.129623 | 0.0333 | 0.00915574 | 0.611091 | 0.254905 | 1.19E-10 | 0.011831191 | 41.48136238 | 0.000373831 |
| 25xisD | TSAT | vokvCt | SCLC | rs17767742 | G | C | G | C | 0.0305 | -0.0384261 | 0.2828 | 0.310028 | 0.466526 | 0.0527728 | 1.95E-09 | 0.005081709 | 36.02301798 | 0.000377354 |
| 25xisD | TSAT | vokvCt | SCLC | rs192458696 | C | T | C | T | -0.1056 | 0.568051 | 0.0139 | 0.00301113 | 0.172948 | 0.416828 | 1.78E-07 | 0.020226173 | 27.25840258 | 0.000305699 |
| 25xisD | TSAT | vokvCt | SCLC | rs1927693 | A | G | A | G | 0.0367 | 0.0262961 | 0.382 | 0.369671 | 0.603544 | 0.0506366 | 2.02E-14 | 0.004797794 | 58.51253778 | 0.000635937 |
| 25xisD | TSAT | vokvCt | SCLC | rs1930884 | T | C | T | C | -0.0239 | 0.0350347 | 0.289 | 0.212083 | 0.565361 | 0.060941 | 7.36E-06 | 0.005331248 | 20.09731459 | 0.000234743 |
| 25xisD | TSAT | vokvCt | SCLC | rs199138 | A | G | A | G | 0.0807 | -0.0115439 | 0.0815 | 0.070768 | 0.904041 | 0.0957535 | 9.59E-21 | 0.008639815 | 87.24452509 | 0.000975021 |
| 25xisD | TSAT | vokvCt | SCLC | rs2088102 | T | C | T | C | -0.0213 | -0.0329212 | 0.4962 | 0.517011 | 0.501359 | 0.0489643 | 4.17E-06 | 0.004627694 | 21.18507172 | 0.000226832 |
| 25xisD | TSAT | vokvCt | SCLC | rs2236252 | T | C | T | C | 0.0359 | 0.0459945 | 0.1788 | 0.147783 | 0.501951 | 0.0685029 | 4.02E-09 | 0.006101947 | 34.61401957 | 0.000378473 |
| 25xisD | TSAT | vokvCt | SCLC | rs2305268 | T | C | T | C | 0.0956 | 0.129755 | 0.016 | 0.0472368 | 0.263421 | 0.116025 | 2.34E-07 | 0.018491028 | 26.72967462 | 0.00028778 |
| 25xisD | TSAT | vokvCt | SCLC | rs28411900 | T | C | T | C | -0.0319 | -0.0218911 | 0.1141 | 0.166606 | 0.738108 | 0.0654717 | 6.64E-06 | 0.007081157 | 20.29424594 | 0.000205722 |
| 25xisD | TSAT | vokvCt | SCLC | rs2875251 | C | T | C | T | -0.0257 | 0.124234 | 0.2637 | 0.247238 | 0.0275588 | 0.0563799 | 2.18E-06 | 0.005426544 | 22.42949983 | 0.000256485 |
| 25xisD | TSAT | vokvCt | SCLC | rs35570672 | T | C | T | C | -0.0429 | 0.0440149 | 0.1877 | 0.251006 | 0.433038 | 0.0561412 | 1.26E-13 | 0.005789228 | 54.9127605 | 0.00056121 |
| 25xisD | TSAT | vokvCt | SCLC | rs35769520 | G | A | G | A | 0.0264 | 0.0109256 | 0.4885 | 0.63611 | 0.829106 | 0.0506173 | 1.34E-08 | 0.004647164 | 32.27244397 | 0.000348296 |
| 25xisD | TSAT | vokvCt | SCLC | rs4671609 | G | A | G | A | -0.0219 | -0.0707414 | 0.3875 | 0.362292 | 0.162524 | 0.0506514 | 4.20E-06 | 0.004759595 | 21.17133433 | 0.000227665 |
| 25xisD | TSAT | vokvCt | SCLC | rs469882 | C | A | C | A | -0.0367 | -0.0112329 | 0.2221 | 0.21455 | 0.851727 | 0.0600959 | 1.03E-10 | 0.005678933 | 41.76366838 | 0.000465409 |
| 25xisD | TSAT | vokvCt | SCLC | rs4764934 | T | C | T | C | -0.0265 | -0.0209661 | 0.2013 | 0.282269 | 0.700156 | 0.0544419 | 6.90E-06 | 0.005893145 | 20.2207609 | 0.000225813 |
| 25xisD | TSAT | vokvCt | SCLC | rs4846335 | A | C | A | C | 0.0445 | 0.0904884 | 0.1105 | 0.144657 | 0.195083 | 0.0698381 | 2.87E-09 | 0.007493013 | 35.27012914 | 0.000389277 |
| 25xisD | TSAT | vokvCt | SCLC | rs4849902 | C | T | C | T | 0.0241 | -0.00693187 | 0.386 | 0.277878 | 0.899549 | 0.0549138 | 6.48E-07 | 0.004842923 | 24.76389518 | 0.000275309 |
| 25xisD | TSAT | vokvCt | SCLC | rs4854737 | G | C | G | C | 0.1444 | -0.0482375 | 0.4575 | 0.362928 | 0.33987 | 0.050541 | 1.00E-200 | 0.004692648 | 946.8876 | 0.010350354 |
| 25xisD | TSAT | vokvCt | SCLC | rs56195124 | A | G | A | G | 0.0882 | -0.0191208 | 0.0543 | 0.0321954 | 0.888466 | 0.136337 | 3.45E-16 | 0.010813521 | 66.52776205 | 0.000798951 |
| 25xisD | TSAT | vokvCt | SCLC | rs59950280 | A | G | A | G | 0.0328 | -0.078012 | 0.3195 | 0.242968 | 0.171853 | 0.0570984 | 5.87E-11 | 0.00500992 | 42.86334951 | 0.000467818 |
| 25xisD | TSAT | vokvCt | SCLC | rs6025 | T | C | T | C | -0.093 | -0.249639 | 0.0188 | 0.0201998 | 0.158164 | 0.176889 | 1.76E-08 | 0.016506701 | 31.74280695 | 0.000319089 |
| 25xisD | TSAT | vokvCt | SCLC | rs60721435 | C | T | C | T | 0.0372 | 0.0134329 | 0.1152 | 0.0857709 | 0.877312 | 0.087013 | 8.41E-08 | 0.006942755 | 28.70927332 | 0.000282107 |
| 25xisD | TSAT | vokvCt | SCLC | rs62183592 | T | C | T | C | 0.0496 | -0.113395 | 0.077 | 0.0397008 | 0.367278 | 0.125773 | 3.31E-08 | 0.008978684 | 30.51672885 | 0.000349692 |
| 25xisD | TSAT | vokvCt | SCLC | rs637828 | C | A | C | A | -0.0221 | -0.0409612 | 0.4011 | 0.40633 | 0.408955 | 0.0496057 | 2.76E-06 | 0.004714247 | 21.97656848 | 0.000234651 |
| 25xisD | TSAT | vokvCt | SCLC | rs715 | C | T | C | T | 0.0247 | -0.00293581 | 0.2726 | 0.313693 | 0.955828 | 0.0530025 | 1.54E-06 | 0.005139437 | 23.09738497 | 0.000241949 |
| 25xisD | TSAT | vokvCt | SCLC | rs722740 | C | T | C | T | 0.0236 | -0.00606721 | 0.3965 | 0.474954 | 0.901463 | 0.0490026 | 6.98E-07 | 0.004756224 | 24.62064385 | 0.000266547 |
| 25xisD | TSAT | vokvCt | SCLC | rs72840508 | T | A | T | A | -0.1212 | 0.0324469 | 0.034 | 0.0182603 | 0.85872 | 0.182283 | 3.40E-20 | 0.013166004 | 84.74171993 | 0.00096492 |
| 25xisD | TSAT | vokvCt | SCLC | rs7297861 | C | T | C | T | 0.0479 | -0.0236922 | 0.0794 | 0.122245 | 0.746956 | 0.0734287 | 3.99E-09 | 0.00813988 | 34.62860702 | 0.000335423 |
| 25xisD | TSAT | vokvCt | SCLC | rs7432894 | C | T | C | T | -0.0377 | 0.0739785 | 0.2955 | 0.272396 | 0.179877 | 0.0551612 | 1.52E-13 | 0.005104669 | 54.54406825 | 0.000591768 |
| 25xisD | TSAT | vokvCt | SCLC | rs7500731 | G | A | G | A | 0.0229 | 0.00618618 | 0.4891 | 0.303077 | 0.906871 | 0.0528794 | 9.45E-07 | 0.00467084 | 24.03703983 | 0.00026208 |
| 25xisD | TSAT | vokvCt | SCLC | rs780093 | T | C | T | C | 0.0235 | 0.0721465 | 0.3416 | 0.352396 | 0.15792 | 0.0510916 | 1.10E-06 | 0.00482264 | 23.74466445 | 0.000248412 |
| 25xisD | TSAT | vokvCt | SCLC | rs79364962 | A | G | A | G | 0.0364 | -0.00341346 | 0.1005 | 0.0635962 | 0.973219 | 0.101679 | 8.50E-06 | 0.008175757 | 19.82197014 | 0.000239552 |
| 25xisD | TSAT | vokvCt | SCLC | rs8062982 | C | G | C | G | 0.0255 | 0.0711767 | 0.3938 | 0.454514 | 0.147097 | 0.0490921 | 1.01E-07 | 0.004788808 | 28.35472965 | 0.000310457 |
| 25xisD | TSAT | vokvCt | SCLC | rs8177257 | T | C | T | C | -0.2878 | 0.0879017 | 0.0735 | 0.0661752 | 0.376352 | 0.0993646 | 1.39E-169 | 0.010368133 | 770.5138685 | 0.011280915 |
| 25xisD | TSAT | vokvCt | SCLC | rs854762 | G | A | G | A | 0.0216 | -0.0564814 | 0.3862 | 0.48629 | 0.250596 | 0.0491612 | 5.38E-06 | 0.004747883 | 20.6970041 | 0.000221196 |
| 25xisD | TSAT | vokvCt | SCLC | rs855791 | A | G | A | G | 0.026 | 0.0607898 | 0.406 | 0.34599 | 0.236556 | 0.0513584 | 2.88E-08 | 0.004685884 | 30.7867347 | 0.000326054 |
| 25xisD | TSAT | vokvCt | SCLC | rs9261848 | C | G | C | G | 0.0297 | 0.218816 | 0.1582 | 0.0859398 | 0.012096 | 0.0872008 | 5.63E-06 | 0.006542097 | 20.61004502 | 0.000234941 |
| 25xisD | TSAT | vokvCt | SCLC | rs9389269 | C | T | C | T | -0.0336 | 0.0405719 | 0.2792 | 0.342575 | 0.430678 | 0.0514851 | 9.10E-11 | 0.005184231 | 42.00585853 | 0.0004544 |
| 25xisD | TSAT | vokvCt | SCLC | rs968155 | C | T | C | T | -0.0547 | -0.00884124 | 0.4638 | 0.439937 | 0.857869 | 0.0493684 | 3.45E-31 | 0.004709348 | 134.9127779 | 0.001488203 |

| **Supplementary Table 6 Statistical characteristics of eligible instrumental variables in forward MR analysis.** | | | | | | | | | | | | | | | | | | |
| --- | --- | --- | --- | --- | --- | --- | --- | --- | --- | --- | --- | --- | --- | --- | --- | --- | --- | --- |
| **FERRITIN AND NSCLC** | | | | | | | | | | | | | | | | | | |
| **id.exposure** | **exposure** | **id.outcome** | **outcome** | **SNP** | **effect_allele.exposure** | **other_allele.exposure** | **effect_allele.outcome** | **other_allele.outcome** | **beta.exposure** | **beta.outcome** | **eaf.exposure** | **eaf.outcome** | **pval.outcome** | **se.outcome** | **pval.exposure** | **se.exposure** | **F** | **R2** |
| BTVhMb | FERRITIN | l2HVXz | NSCLC | rs10184004 | T | C | T | C | -0.0159 | -0.0229255 | 0.4707 | 0.35825 | 0.249047 | 0.0198891 | 5.13E-06 | 0.003487301 | 20.78812789 | 0.000125971 |
| BTVhMb | FERRITIN | l2HVXz | NSCLC | rs10220355 | A | T | A | T | 0.0157 | 0.0232859 | 0.4252 | 0.424637 | 0.229692 | 0.0193863 | 7.29E-06 | 0.003500526 | 20.11558603 | 0.000120487 |
| BTVhMb | FERRITIN | l2HVXz | NSCLC | rs10263500 | T | C | T | C | 0.0225 | -0.0079471 | 0.4605 | 0.49849 | 0.678138 | 0.0191494 | 6.59E-11 | 0.003445792 | 42.63702748 | 0.000251545 |
| BTVhMb | FERRITIN | l2HVXz | NSCLC | rs1065853 | T | G | T | G | 0.0361 | 0.0593178 | 0.0594 | 0.0520472 | 0.154202 | 0.041631 | 1.25E-07 | 0.006829323 | 27.94210347 | 0.000145625 |
| BTVhMb | FERRITIN | l2HVXz | NSCLC | rs107421 | G | A | G | A | -0.0224 | -0.00979217 | 0.2209 | 0.182255 | 0.690307 | 0.0245763 | 1.15E-07 | 0.004225403 | 28.10345819 | 0.000172709 |
| BTVhMb | FERRITIN | l2HVXz | NSCLC | rs10750215 | T | G | T | G | 0.0213 | -0.0163048 | 0.4199 | 0.393371 | 0.404143 | 0.0195444 | 3.66E-08 | 0.003868143 | 30.32174317 | 0.000221023 |
| BTVhMb | FERRITIN | l2HVXz | NSCLC | rs10801913 | A | G | A | G | 0.0237 | -0.00279553 | 0.3004 | 0.301052 | 0.892877 | 0.0207592 | 2.63E-10 | 0.003750502 | 39.93170824 | 0.000236089 |
| BTVhMb | FERRITIN | l2HVXz | NSCLC | rs10804630 | C | T | C | T | -0.0335 | 0.0109997 | 0.1022 | 0.144916 | 0.687276 | 0.0273248 | 3.71E-09 | 0.005681211 | 34.77024698 | 0.000205944 |
| BTVhMb | FERRITIN | l2HVXz | NSCLC | rs10838599 | T | G | T | G | -0.0182 | -0.0224499 | 0.2579 | 0.227123 | 0.323626 | 0.0227448 | 3.85E-06 | 0.003939974 | 21.33811563 | 0.00012679 |
| BTVhMb | FERRITIN | l2HVXz | NSCLC | rs10842942 | G | A | G | A | -0.0192 | 0.0331911 | 0.2173 | 0.156087 | 0.205323 | 0.0262063 | 5.19E-06 | 0.004213338 | 20.7658569 | 0.000125397 |
| BTVhMb | FERRITIN | l2HVXz | NSCLC | rs11125069 | A | G | A | G | 0.0157 | 0.0104798 | 0.4607 | 0.404337 | 0.591473 | 0.0195263 | 6.41E-06 | 0.003479306 | 20.36170086 | 0.000122484 |
| BTVhMb | FERRITIN | l2HVXz | NSCLC | rs111405904 | A | G | A | G | 0.0798 | 0.0056282 | 0.0109 | 0.0195065 | 0.934945 | 0.0689517 | 1.76E-06 | 0.016697379 | 22.84068754 | 0.00013731 |
| BTVhMb | FERRITIN | l2HVXz | NSCLC | rs1121985 | A | C | A | C | 0.018 | -0.0192446 | 0.3231 | 0.324806 | 0.347959 | 0.0205045 | 1.12E-06 | 0.003696638 | 23.70997866 | 0.000141722 |
| BTVhMb | FERRITIN | l2HVXz | NSCLC | rs112229166 | T | G | T | G | 0.0224 | -0.0290421 | 0.1285 | 0.153862 | 0.275593 | 0.0266375 | 9.02E-06 | 0.0050457 | 19.7084825 | 0.000112382 |
| BTVhMb | FERRITIN | l2HVXz | NSCLC | rs11230848 | T | C | T | C | -0.031 | 0.00684816 | 0.0895 | 0.155749 | 0.798438 | 0.0268167 | 2.98E-07 | 0.006049126 | 26.26262462 | 0.000156623 |
| BTVhMb | FERRITIN | l2HVXz | NSCLC | rs112579095 | T | C | T | C | 0.0357 | -0.086268 | 0.0596 | 0.0877306 | 0.0107798 | 0.0338338 | 3.83E-07 | 0.007031409 | 25.7781479 | 0.000142865 |
| BTVhMb | FERRITIN | l2HVXz | NSCLC | rs114007478 | G | A | G | A | 0.0379 | 0.0307596 | 0.0535 | 0.047043 | 0.500552 | 0.0456631 | 2.73E-06 | 0.008080759 | 21.99753973 | 0.000145473 |
| BTVhMb | FERRITIN | l2HVXz | NSCLC | rs11614077 | T | C | T | C | 0.022 | 0.0261158 | 0.2255 | 0.329852 | 0.200835 | 0.0204161 | 6.59E-08 | 0.004072562 | 29.18165726 | 0.000169061 |
| BTVhMb | FERRITIN | l2HVXz | NSCLC | rs11634990 | C | T | C | T | -0.0265 | -0.0913963 | 0.1708 | 0.167715 | 0.000409562 | 0.0258631 | 9.40E-09 | 0.004615748 | 32.96154556 | 0.000198916 |
| BTVhMb | FERRITIN | l2HVXz | NSCLC | rs11708600 | T | C | T | C | 0.03 | 0.0223409 | 0.0865 | 0.108881 | 0.469285 | 0.0308728 | 1.53E-06 | 0.006240539 | 23.10991277 | 0.000142232 |
| BTVhMb | FERRITIN | l2HVXz | NSCLC | rs117940828 | C | G | C | G | -0.0641 | 0.234587 | 0.0206 | 0.00825001 | 0.0247195 | 0.104458 | 4.41E-07 | 0.012692188 | 25.50604176 | 0.000165796 |
| BTVhMb | FERRITIN | l2HVXz | NSCLC | rs11977873 | G | A | G | A | -0.0276 | 0.0118596 | 0.1463 | 0.123065 | 0.682989 | 0.02904 | 6.76E-08 | 0.005113539 | 29.13231646 | 0.000190282 |
| BTVhMb | FERRITIN | l2HVXz | NSCLC | rs12143544 | A | T | A | T | 0.0182 | -0.0112235 | 0.4619 | 0.499812 | 0.563117 | 0.0194106 | 3.36E-07 | 0.003567196 | 26.03087887 | 0.000164658 |
| BTVhMb | FERRITIN | l2HVXz | NSCLC | rs12200559 | T | C | T | C | 0.0189 | -0.0375097 | 0.2305 | 0.217883 | 0.104177 | 0.0230839 | 4.94E-06 | 0.004138094 | 20.86041311 | 0.000126716 |
| BTVhMb | FERRITIN | l2HVXz | NSCLC | rs12419620 | G | T | G | T | -0.0312 | -0.0373471 | 0.1621 | 0.142087 | 0.1771 | 0.0276699 | 3.43E-11 | 0.004708142 | 43.91471638 | 0.000264432 |
| BTVhMb | FERRITIN | l2HVXz | NSCLC | rs1250244 | G | C | G | C | -0.0247 | -0.0134774 | 0.3476 | 0.200516 | 0.572539 | 0.0238826 | 3.56E-11 | 0.003730373 | 43.84190214 | 0.000276705 |
| BTVhMb | FERRITIN | l2HVXz | NSCLC | rs12568930 | C | T | C | T | 0.0345 | -0.0595369 | 0.185 | 0.0899527 | 0.0730146 | 0.0332101 | 1.88E-14 | 0.004504751 | 58.65386151 | 0.00035892 |
| BTVhMb | FERRITIN | l2HVXz | NSCLC | rs1260326 | T | C | T | C | 0.0254 | -0.0417272 | 0.3414 | 0.349672 | 0.0379254 | 0.0201031 | 1.48E-12 | 0.003589421 | 50.07473211 | 0.000290123 |
| BTVhMb | FERRITIN | l2HVXz | NSCLC | rs12685912 | A | C | A | C | 0.0193 | -0.0073943 | 0.2185 | 0.249283 | 0.737724 | 0.0220812 | 3.58E-06 | 0.004164522 | 21.47752814 | 0.000127211 |
| BTVhMb | FERRITIN | l2HVXz | NSCLC | rs12807014 | C | T | C | T | -0.0286 | 0.0264952 | 0.2431 | 0.223227 | 0.247256 | 0.0228991 | 2.72E-13 | 0.003913745 | 53.40070795 | 0.000301013 |
| BTVhMb | FERRITIN | l2HVXz | NSCLC | rs12892462 | G | C | G | C | 0.0186 | 0.0106087 | 0.3278 | 0.326894 | 0.604682 | 0.0204929 | 4.43E-07 | 0.003683543 | 25.49731213 | 0.000152463 |
| BTVhMb | FERRITIN | l2HVXz | NSCLC | rs12916360 | T | G | T | G | -0.027 | -0.0198367 | 0.1601 | 0.228729 | 0.382695 | 0.022724 | 1.98E-08 | 0.004809628 | 31.51407425 | 0.000196054 |
| BTVhMb | FERRITIN | l2HVXz | NSCLC | rs12979144 | A | G | A | G | 0.0197 | -0.0252727 | 0.3689 | 0.440842 | 0.189129 | 0.0192457 | 2.68E-08 | 0.003542432 | 30.92640277 | 0.000180705 |
| BTVhMb | FERRITIN | l2HVXz | NSCLC | rs13253974 | A | G | A | G | 0.0244 | 0.00543195 | 0.3396 | 0.291982 | 0.794806 | 0.020886 | 2.51E-11 | 0.003656646 | 44.52601375 | 0.000267045 |
| BTVhMb | FERRITIN | l2HVXz | NSCLC | rs13408333 | T | A | T | A | -0.0287 | -0.00629391 | 0.0951 | 0.088879 | 0.849214 | 0.0331046 | 1.64E-06 | 0.005987433 | 22.97642523 | 0.000141767 |
| BTVhMb | FERRITIN | l2HVXz | NSCLC | rs139977145 | T | G | T | G | 0.0288 | -0.00541087 | 0.0771 | 0.190432 | 0.825305 | 0.0245139 | 6.71E-06 | 0.006396182 | 20.27418242 | 0.000118039 |
| BTVhMb | FERRITIN | l2HVXz | NSCLC | rs143854232 | G | A | G | A | 0.0834 | -0.132561 | 0.0112 | 0.0131787 | 0.129306 | 0.0873926 | 7.98E-06 | 0.018675601 | 19.94265278 | 0.00015406 |
| BTVhMb | FERRITIN | l2HVXz | NSCLC | rs143922167 | A | G | A | G | 0.1148 | -0.0325953 | 0.015 | 0.00953036 | 0.734042 | 0.0959383 | 7.69E-16 | 0.014244884 | 64.94802394 | 0.000389441 |
| BTVhMb | FERRITIN | l2HVXz | NSCLC | rs144861591 | T | C | T | C | 0.1333 | 0.0349564 | 0.0671 | 0.037811 | 0.48509 | 0.0500708 | 7.35E-85 | 0.006828715 | 381.0503138 | 0.002224579 |
| BTVhMb | FERRITIN | l2HVXz | NSCLC | rs161044 | T | C | T | C | 0.0731 | 0.0162011 | 0.0415 | 0.0234760000000001 | 0.792029 | 0.061443 | 1.25E-19 | 0.00806429 | 82.16795527 | 0.000425114 |
| BTVhMb | FERRITIN | l2HVXz | NSCLC | rs165316 | G | A | G | A | 0.0226 | 0.0269422 | 0.2224 | 0.214985 | 0.250725 | 0.0234567 | 8.18E-08 | 0.004213971 | 28.7629743 | 0.00017666 |
| BTVhMb | FERRITIN | l2HVXz | NSCLC | rs16932140 | G | A | G | A | 0.0412 | -0.110628 | 0.0464 | 0.0218237 | 0.0920492 | 0.0656667 | 3.89E-07 | 0.008119405 | 25.74814953 | 0.000150213 |
| BTVhMb | FERRITIN | l2HVXz | NSCLC | rs1694067 | T | C | T | C | -0.0211 | -0.00635799 | 0.3983 | 0.371456 | 0.748826 | 0.019857 | 2.53E-09 | 0.003540561 | 35.5157302 | 0.000213395 |
| BTVhMb | FERRITIN | l2HVXz | NSCLC | rs17050272 | A | G | A | G | 0.0208 | 0.00520525 | 0.4169 | 0.536909 | 0.786287 | 0.019198 | 4.13E-09 | 0.003538076 | 34.56148184 | 0.000210345 |
| BTVhMb | FERRITIN | l2HVXz | NSCLC | rs17112021 | G | T | G | T | 0.0244 | 0.0456454 | 0.2784 | 0.20295 | 0.0539449 | 0.0236839 | 7.66E-10 | 0.003966308 | 37.84484936 | 0.000239208 |
| BTVhMb | FERRITIN | l2HVXz | NSCLC | rs17476364 | C | T | C | T | 0.0431 | -0.040453 | 0.0948 | 0.0588121 | 0.316065 | 0.040349 | 3.57E-14 | 0.00568919 | 57.39230461 | 0.000318814 |
| BTVhMb | FERRITIN | l2HVXz | NSCLC | rs17505399 | G | A | G | A | 0.0647 | 0.0980103 | 0.015 | 0.0357939 | 0.0562536 | 0.0513393 | 5.05E-06 | 0.014180201 | 20.81822929 | 0.000123699 |
| BTVhMb | FERRITIN | l2HVXz | NSCLC | rs17540759 | T | A | T | A | 0.0435 | -0.027957 | 0.0424 | 0.00997805 | 0.766466 | 0.0941308 | 5.70E-07 | 0.008698062 | 25.01114165 | 0.000153659 |
| BTVhMb | FERRITIN | l2HVXz | NSCLC | rs1765028 | G | A | G | A | -0.0186 | -0.0399502 | 0.4679 | 0.473957 | 0.0371321 | 0.0191671 | 7.54E-08 | 0.003458661 | 28.92077288 | 0.000172267 |
| BTVhMb | FERRITIN | l2HVXz | NSCLC | rs17676097 | G | A | G | A | -0.0272 | -0.0419897 | 0.3577 | 0.320659 | 0.04115 | 0.0205629 | 6.06E-14 | 0.003623392 | 56.35171863 | 0.000339958 |
| BTVhMb | FERRITIN | l2HVXz | NSCLC | rs181804134 | G | T | G | T | 0.0488 | 0.0772841 | 0.0303 | 0.0224704 | 0.226802 | 0.063943 | 7.89E-06 | 0.010921753 | 19.96433933 | 0.000139943 |
| BTVhMb | FERRITIN | l2HVXz | NSCLC | rs1848797 | G | A | G | A | -0.0167 | 0.0222419 | 0.4119 | 0.357444 | 0.263947 | 0.0199102 | 1.85E-06 | 0.003501668 | 22.74484636 | 0.000135116 |
| BTVhMb | FERRITIN | l2HVXz | NSCLC | rs1848887 | C | T | C | T | -0.0706 | 0.0198701 | 0.02 | 0.00654796 | 0.872028 | 0.123354 | 5.61E-08 | 0.012999911 | 29.49365827 | 0.000195387 |
| BTVhMb | FERRITIN | l2HVXz | NSCLC | rs1894692 | G | A | G | A | 0.1472 | 0.0505684 | 0.019 | 0.020118 | 0.465117 | 0.0692292 | 1.63E-37 | 0.011499594 | 163.8515691 | 0.000807734 |
| BTVhMb | FERRITIN | l2HVXz | NSCLC | rs191085215 | C | T | C | T | -0.0505 | 0.0816969 | 0.0232 | 0.0104632 | 0.38438 | 0.0939203 | 3.31E-06 | 0.010858857 | 21.62790874 | 0.000115586 |
| BTVhMb | FERRITIN | l2HVXz | NSCLC | rs191983110 | G | A | G | A | -0.0509 | -0.006915 | 0.0271 | 0.0160004 | 0.927575 | 0.0760756 | 5.39E-06 | 0.011189259 | 20.69345044 | 0.000136616 |
| BTVhMb | FERRITIN | l2HVXz | NSCLC | rs1929524 | T | A | T | A | -0.0183 | -0.0137908 | 0.3754 | 0.377705 | 0.483881 | 0.0196991 | 2.41E-07 | 0.003543378 | 26.67271415 | 0.000157047 |
| BTVhMb | FERRITIN | l2HVXz | NSCLC | rs199138 | A | G | A | G | -0.1464 | -0.036828 | 0.0815 | 0.070717 | 0.327436 | 0.0376068 | 5.02E-115 | 0.00642217 | 519.6591259 | 0.003208846 |
| BTVhMb | FERRITIN | l2HVXz | NSCLC | rs2008598 | G | A | G | A | -0.0205 | -0.0287776 | 0.3355 | 0.18039 | 0.245457 | 0.0247772 | 2.95E-08 | 0.003697439 | 30.74012906 | 0.000187381 |
| BTVhMb | FERRITIN | l2HVXz | NSCLC | rs200896712 | C | A | C | A | -0.0416 | 0.0407326 | 0.0124 | 0.0822151 | 0.23638 | 0.0344002 | 9.90E-07 | 0.008500872 | 23.94747383 | 4.23857E-05 |
| BTVhMb | FERRITIN | l2HVXz | NSCLC | rs2049722 | G | C | G | C | -0.0156 | -0.0546548 | 0.4284 | 0.435319 | 0.00469418 | 0.0193311 | 7.81E-06 | 0.003489677 | 19.98383037 | 0.000119185 |
| BTVhMb | FERRITIN | l2HVXz | NSCLC | rs2062059 | G | A | G | A | 0.0177 | 0.00560238 | 0.4535 | 0.426217 | 0.771879 | 0.019324 | 3.48E-07 | 0.003473719 | 25.96313697 | 0.00015529 |
| BTVhMb | FERRITIN | l2HVXz | NSCLC | rs2072576 | C | G | C | G | 0.0256 | 0.00620881 | 0.1173 | 0.120042 | 0.833824 | 0.029594 | 6.41E-06 | 0.005673264 | 20.36169574 | 0.000135713 |
| BTVhMb | FERRITIN | l2HVXz | NSCLC | rs2093202 | G | A | G | A | 0.0166 | 0.00624406 | 0.3503 | 0.430532 | 0.745837 | 0.0192638 | 3.53E-06 | 0.003579674 | 21.50449367 | 0.000125429 |
| BTVhMb | FERRITIN | l2HVXz | NSCLC | rs2126588 | G | A | G | A | 0.0177 | -0.0103477 | 0.3995 | 0.405888 | 0.59478 | 0.0194533 | 4.31E-07 | 0.003501671 | 25.55029118 | 0.000150316 |
| BTVhMb | FERRITIN | l2HVXz | NSCLC | rs2236953 | T | G | T | G | 0.0247 | -0.00758218 | 0.1129 | 0.157469 | 0.773207 | 0.0263104 | 3.01E-06 | 0.005288925 | 21.81017195 | 0.000122205 |
| BTVhMb | FERRITIN | l2HVXz | NSCLC | rs2473236 | C | T | C | T | 0.0199 | -0.0170774 | 0.251 | 0.213497 | 0.464636 | 0.0233542 | 7.07E-07 | 0.004012557 | 24.59595689 | 0.000148899 |
| BTVhMb | FERRITIN | l2HVXz | NSCLC | rs2529440 | T | C | T | C | -0.0346 | -0.0343165 | 0.4503 | 0.401883 | 0.0773393 | 0.0194281 | 4.60E-23 | 0.003498481 | 97.81222953 | 0.000592666 |
| BTVhMb | FERRITIN | l2HVXz | NSCLC | rs2595582 | A | G | A | G | -0.0195 | 0.032491 | 0.4151 | 0.329951 | 0.10871 | 0.020256 | 2.75E-08 | 0.003509308 | 30.87637095 | 0.000184643 |
| BTVhMb | FERRITIN | l2HVXz | NSCLC | rs2606177 | A | G | A | G | -0.0167 | 0.0399871 | 0.3495 | 0.368453 | 0.0433621 | 0.0197936 | 3.44E-06 | 0.003597098 | 21.554026 | 0.000126811 |
| BTVhMb | FERRITIN | l2HVXz | NSCLC | rs28406133 | G | C | G | C | -0.1036 | 0.243555 | 0.0061 | 0.0016946 | 0.260833 | 0.216604 | 4.13E-06 | 0.022498601 | 21.20354534 | 0.000130143 |
| BTVhMb | FERRITIN | l2HVXz | NSCLC | rs28590023 | G | A | G | A | 0.0184 | 0.019545 | 0.4754 | 0.491052 | 0.308724 | 0.0192012 | 9.89E-08 | 0.003452978 | 28.39540212 | 0.00016887 |
| BTVhMb | FERRITIN | l2HVXz | NSCLC | rs28678167 | T | G | T | G | 0.0192 | -0.0284975 | 0.2673 | 0.396996 | 0.148345 | 0.019716 | 4.92E-07 | 0.003817548 | 25.29492783 | 0.000144397 |
| BTVhMb | FERRITIN | l2HVXz | NSCLC | rs28715334 | T | G | T | G | 0.025 | 0.0142621 | 0.2041 | 0.153164 | 0.590281 | 0.0264883 | 8.22E-09 | 0.004337353 | 33.22235994 | 0.000203054 |
| BTVhMb | FERRITIN | l2HVXz | NSCLC | rs2943644 | T | C | T | C | -0.0185 | 0.00199946 | 0.4791 | 0.563002 | 0.917742 | 0.0193598 | 9.16E-08 | 0.003462704 | 28.54386111 | 0.000170826 |
| BTVhMb | FERRITIN | l2HVXz | NSCLC | rs2954029 | T | A | T | A | -0.0245 | 0.0178747 | 0.4905 | 0.458823 | 0.350685 | 0.0191529 | 1.42E-12 | 0.003459432 | 50.15596285 | 0.000300017 |
| BTVhMb | FERRITIN | l2HVXz | NSCLC | rs31474 | C | T | C | T | 0.0203 | 0.00383264 | 0.1891 | 0.379213 | 0.846149 | 0.0197525 | 2.14E-06 | 0.004282942 | 22.46506552 | 0.000126381 |
| BTVhMb | FERRITIN | l2HVXz | NSCLC | rs34220530 | T | C | T | C | -0.0152 | 0.0241218 | 0.4856 | 0.46909 | 0.207697 | 0.0191454 | 9.70E-06 | 0.003435995 | 19.56960814 | 0.000115424 |
| BTVhMb | FERRITIN | l2HVXz | NSCLC | rs34523089 | T | C | T | C | 0.0686 | 0.00134062 | 0.1637 | 0.15781 | 0.959544 | 0.0264286 | 3.16E-48 | 0.00470122 | 212.9252085 | 0.001288514 |
| BTVhMb | FERRITIN | l2HVXz | NSCLC | rs34787647 | G | A | G | A | 0.0192 | -0.0211641 | 0.27 | 0.283027 | 0.317445 | 0.02117 | 5.11E-07 | 0.003823075 | 25.2218432 | 0.000145318 |
| BTVhMb | FERRITIN | l2HVXz | NSCLC | rs34877685 | A | G | A | G | 0.0633 | 0.0375393 | 0.0181 | 0.00325585 | 0.827288 | 0.172055 | 4.35E-07 | 0.012527294 | 25.53247294 | 0.000142424 |
| BTVhMb | FERRITIN | l2HVXz | NSCLC | rs350415 | G | A | G | A | -0.0201 | 0.0281775 | 0.2726 | 0.228541 | 0.213077 | 0.0226298 | 2.56E-07 | 0.003900445 | 26.5560696 | 0.000160222 |
| BTVhMb | FERRITIN | l2HVXz | NSCLC | rs35107257 | A | G | A | G | 0.0431 | 0.0714758 | 0.0591 | 0.0368486 | 0.169602 | 0.0520399 | 3.27E-08 | 0.007799028 | 30.54031986 | 0.000206593 |
| BTVhMb | FERRITIN | l2HVXz | NSCLC | rs35583916 | A | G | A | G | 0.0671 | 0.029341 | 0.0113 | 0.013172 | 0.72687 | 0.0840012 | 6.46E-06 | 0.014875588 | 20.34683004 | 0.000100605 |
| BTVhMb | FERRITIN | l2HVXz | NSCLC | rs358383 | A | G | A | G | -0.0366 | 0.0721174 | 0.0423 | 0.0400782 | 0.13835 | 0.0486634 | 7.99E-06 | 0.008196259 | 19.94026032 | 0.000108533 |
| BTVhMb | FERRITIN | l2HVXz | NSCLC | rs36184164 | G | T | G | T | 0.0356 | 0.105589 | 0.1398 | 0.0909737 | 0.00101887 | 0.0321403 | 6.46E-12 | 0.005182616 | 47.18477984 | 0.000304815 |
| BTVhMb | FERRITIN | l2HVXz | NSCLC | rs370631 | C | T | C | T | -0.0257 | 0.0400196 | 0.2558 | 0.199429 | 0.0921425 | 0.0237618 | 3.27E-11 | 0.003874059 | 44.00821824 | 0.00025147 |
| BTVhMb | FERRITIN | l2HVXz | NSCLC | rs3738182 | A | G | A | G | -0.0233 | 0.0480681 | 0.1824 | 0.240949 | 0.0328526 | 0.0225262 | 1.35E-07 | 0.004419638 | 27.79319305 | 0.000161923 |
| BTVhMb | FERRITIN | l2HVXz | NSCLC | rs3765602 | A | C | A | C | -0.0239 | 0.0438804 | 0.1921 | 0.0907281 | 0.185611 | 0.0331504 | 9.00E-08 | 0.004470768 | 28.57798042 | 0.000177301 |
| BTVhMb | FERRITIN | l2HVXz | NSCLC | rs3791778 | C | T | C | T | -0.029 | 0.0699191 | 0.0862 | 0.100686 | 0.0273162 | 0.0316811 | 5.73E-06 | 0.006393136 | 20.57633914 | 0.00013249 |
| BTVhMb | FERRITIN | l2HVXz | NSCLC | rs4333182 | G | A | G | A | -0.0185 | 0.0237948 | 0.3548 | 0.299342 | 0.255284 | 0.0209165 | 4.58E-07 | 0.003668363 | 25.4330736 | 0.000156694 |
| BTVhMb | FERRITIN | l2HVXz | NSCLC | rs4433065 | A | G | A | G | -0.0159 | -0.0259268 | 0.4618 | 0.574784 | 0.180827 | 0.0193742 | 4.81E-06 | 0.003476999 | 20.91149667 | 0.000125667 |
| BTVhMb | FERRITIN | l2HVXz | NSCLC | rs4492136 | T | C | T | C | -0.0303 | -0.0211186 | 0.0612 | 0.0736619 | 0.566144 | 0.0368087 | 9.71E-06 | 0.006849729 | 19.56764127 | 0.000105497 |
| BTVhMb | FERRITIN | l2HVXz | NSCLC | rs45520632 | C | T | C | T | 0.1061 | 0.104979 | 0.0491 | 0.0222838 | 0.105616 | 0.0648735 | 2.39E-39 | 0.008084244 | 172.2471092 | 0.00105118 |
| BTVhMb | FERRITIN | l2HVXz | NSCLC | rs4655583 | A | C | A | C | 0.0198 | 0.0236129 | 0.2161 | 0.33404 | 0.245019 | 0.0203116 | 2.87E-06 | 0.00423085 | 21.90156375 | 0.000132824 |
| BTVhMb | FERRITIN | l2HVXz | NSCLC | rs4681485 | C | T | C | T | -0.0175 | 0.0028809 | 0.3448 | 0.391836 | 0.883554 | 0.0196694 | 1.76E-06 | 0.003661706 | 22.84068656 | 0.000138372 |
| BTVhMb | FERRITIN | l2HVXz | NSCLC | rs4789111 | T | C | T | C | 0.0343 | -0.0046419 | 0.1454 | 0.118387 | 0.874098 | 0.0292947 | 1.33E-12 | 0.004837014 | 50.28443229 | 0.000292379 |
| BTVhMb | FERRITIN | l2HVXz | NSCLC | rs4793897 | T | A | T | A | 0.0155 | -0.00517779 | 0.4137 | 0.372263 | 0.79401 | 0.0198301 | 9.18E-06 | 0.003494424 | 19.67488477 | 0.000116546 |
| BTVhMb | FERRITIN | l2HVXz | NSCLC | rs4808802 | C | G | C | G | 0.0275 | 0.0358894 | 0.2303 | 0.218757 | 0.120842 | 0.0231358 | 3.42E-11 | 0.004149535 | 43.92042394 | 0.000268109 |
| BTVhMb | FERRITIN | l2HVXz | NSCLC | rs4841429 | G | A | G | A | 0.0601 | 0.0577965 | 0.0791 | 0.0876069 | 0.0875891 | 0.0338337 | 8.21E-21 | 0.006423058 | 87.55183575 | 0.000526221 |
| BTVhMb | FERRITIN | l2HVXz | NSCLC | rs4910115 | A | T | A | T | -0.0183 | 0.0257783 | 0.465 | 0.447528 | 0.179851 | 0.0192202 | 9.94E-08 | 0.003434802 | 28.38564488 | 0.000166625 |
| BTVhMb | FERRITIN | l2HVXz | NSCLC | rs4938939 | A | G | A | G | 0.0224 | -0.0275605 | 0.2915 | 0.226588 | 0.225145 | 0.0227217 | 3.01E-09 | 0.003776733 | 35.17737841 | 0.000207255 |
| BTVhMb | FERRITIN | l2HVXz | NSCLC | rs55778511 | G | T | G | T | -0.0527 | 0.0206776 | 0.0584 | 0.0743737 | 0.571871 | 0.0365782 | 3.73E-12 | 0.007585952 | 48.26152597 | 0.000305443 |
| BTVhMb | FERRITIN | l2HVXz | NSCLC | rs56206139 | C | A | C | A | 0.0455 | -0.10959 | 0.0788 | 0.0342578 | 0.0358534 | 0.0522205 | 4.07E-12 | 0.006561179 | 48.09046993 | 0.000300561 |
| BTVhMb | FERRITIN | l2HVXz | NSCLC | rs56343080 | A | G | A | G | -0.0216 | 0.0346776 | 0.181 | 0.170813 | 0.175369 | 0.0255894 | 1.78E-06 | 0.004521741 | 22.81897521 | 0.000138325 |
| BTVhMb | FERRITIN | l2HVXz | NSCLC | rs590097 | T | G | T | G | -0.0198 | 0.0232617 | 0.3409 | 0.386603 | 0.237847 | 0.0197069 | 4.93E-08 | 0.003630485 | 29.74411743 | 0.000176173 |
| BTVhMb | FERRITIN | l2HVXz | NSCLC | rs6005258 | A | T | A | T | 0.0189 | 0.0271167 | 0.2096 | 0.175112 | 0.278601 | 0.0250277 | 9.82E-06 | 0.004274955 | 19.54611787 | 0.000118356 |
| BTVhMb | FERRITIN | l2HVXz | NSCLC | rs6059696 | G | C | G | C | -0.0374 | -0.0357478 | 0.1683 | 0.206374 | 0.131486 | 0.0237012 | 1.39E-15 | 0.004682994 | 63.78168696 | 0.000391583 |
| BTVhMb | FERRITIN | l2HVXz | NSCLC | rs60796549 | C | T | C | T | 0.0216 | -0.0209795 | 0.1647 | 0.125686 | 0.477459 | 0.0295322 | 9.38E-06 | 0.004874753 | 19.63370547 | 0.000128373 |
| BTVhMb | FERRITIN | l2HVXz | NSCLC | rs6089946 | G | A | G | A | 0.0205 | -0.0142681 | 0.2569 | 0.182595 | 0.568507 | 0.0250207 | 5.79E-07 | 0.004101564 | 24.98093774 | 0.000160453 |
| BTVhMb | FERRITIN | l2HVXz | NSCLC | rs61160702 | G | A | G | A | 0.0261 | 0.0107555 | 0.1146 | 0.134682 | 0.700898 | 0.0280011 | 1.03E-06 | 0.005341991 | 23.87122524 | 0.00013824 |
| BTVhMb | FERRITIN | l2HVXz | NSCLC | rs61405481 | G | A | G | A | 0.0162 | 0.0200937 | 0.4444 | 0.490972 | 0.297101 | 0.0192714 | 3.43E-06 | 0.003488949 | 21.55960436 | 0.000129597 |
| BTVhMb | FERRITIN | l2HVXz | NSCLC | rs62074125 | C | A | C | A | 0.0212 | 0.0118294 | 0.2904 | 0.241896 | 0.597086 | 0.0223789 | 4.79E-08 | 0.003883542 | 29.79996192 | 0.00018523 |
| BTVhMb | FERRITIN | l2HVXz | NSCLC | rs62337886 | A | G | A | G | 0.0228 | -0.0136822 | 0.1541 | 0.181142 | 0.582861 | 0.0249125 | 9.21E-07 | 0.004645659 | 24.08657428 | 0.000135526 |
| BTVhMb | FERRITIN | l2HVXz | NSCLC | rs653060 | T | C | T | C | 0.0176 | 0.0225401 | 0.4448 | 0.454599 | 0.24262 | 0.0192903 | 4.18E-07 | 0.003477868 | 25.60938483 | 0.000152992 |
| BTVhMb | FERRITIN | l2HVXz | NSCLC | rs6597 | G | T | G | T | 0.0222 | 0.0719099 | 0.1772 | 0.111686 | 0.0186638 | 0.0305716 | 1.78E-06 | 0.004647345 | 22.81897439 | 0.000143712 |
| BTVhMb | FERRITIN | l2HVXz | NSCLC | rs6704070 | C | T | C | T | -0.0181 | -0.0117176 | 0.4995 | 0.427414 | 0.543881 | 0.0193057 | 1.45E-07 | 0.003441851 | 27.65495899 | 0.000163805 |
| BTVhMb | FERRITIN | l2HVXz | NSCLC | rs672496 | A | G | A | G | 0.0239 | -0.00474554 | 0.1538 | 0.132753 | 0.867086 | 0.028355 | 1.16E-06 | 0.004915318 | 23.64245449 | 0.000148681 |
| BTVhMb | FERRITIN | l2HVXz | NSCLC | rs6750720 | G | C | G | C | -0.017 | -0.0198067 | 0.317 | 0.383924 | 0.313814 | 0.0196641 | 3.53E-06 | 0.003665931 | 21.50449607 | 0.000125143 |
| BTVhMb | FERRITIN | l2HVXz | NSCLC | rs6760824 | A | C | A | C | 0.0316 | -0.0126038 | 0.2436 | 0.245477 | 0.567496 | 0.0220445 | 8.53E-16 | 0.003927247 | 64.74373235 | 0.000367987 |
| BTVhMb | FERRITIN | l2HVXz | NSCLC | rs6822746 | A | G | A | G | -0.0214 | -0.00941787 | 0.3134 | 0.38224 | 0.631787 | 0.0196528 | 5.51E-09 | 0.003670047 | 34.000421 | 0.000197088 |
| BTVhMb | FERRITIN | l2HVXz | NSCLC | rs7009799 | T | C | T | C | -0.0172 | -0.0645807 | 0.3641 | 0.420037 | 0.000873796 | 0.0194035 | 1.63E-06 | 0.003587369 | 22.98819074 | 0.000136992 |
| BTVhMb | FERRITIN | l2HVXz | NSCLC | rs704017 | A | G | A | G | 0.018 | 0.0236951 | 0.3742 | 0.331488 | 0.241838 | 0.0202452 | 3.09E-07 | 0.003517086 | 26.19262556 | 0.000151745 |
| BTVhMb | FERRITIN | l2HVXz | NSCLC | rs7042119 | T | C | T | C | 0.0194 | -0.0110797 | 0.2238 | 0.236755 | 0.622616 | 0.022513 | 1.89E-06 | 0.004071487 | 22.7037374 | 0.000130758 |
| BTVhMb | FERRITIN | l2HVXz | NSCLC | rs7068127 | G | A | G | A | 0.0197 | -0.0049281 | 0.4108 | 0.375613 | 0.803232 | 0.0197784 | 1.69E-08 | 0.003492247 | 31.82163914 | 0.000187869 |
| BTVhMb | FERRITIN | l2HVXz | NSCLC | rs708686 | T | C | T | C | -0.0307 | -0.0262331 | 0.2304 | 0.334582 | 0.198037 | 0.0203806 | 1.96E-14 | 0.004011381 | 58.57184774 | 0.000334237 |
| BTVhMb | FERRITIN | l2HVXz | NSCLC | rs71537957 | T | C | T | C | 0.0228 | -0.00199753 | 0.2676 | 0.334195 | 0.921655 | 0.0203106 | 5.05E-09 | 0.003900425 | 34.17006706 | 0.000203767 |
| BTVhMb | FERRITIN | l2HVXz | NSCLC | rs71649623 | A | C | A | C | -0.0725 | -0.0356071 | 0.0147 | 0.0127023 | 0.675136 | 0.0849588 | 2.29E-07 | 0.014012072 | 26.77141292 | 0.000152262 |
| BTVhMb | FERRITIN | l2HVXz | NSCLC | rs72494581 | C | T | C | T | 0.0175 | -0.0255797 | 0.3179 | 0.343369 | 0.204925 | 0.0201789 | 2.83E-06 | 0.00373709 | 21.92850293 | 0.000132814 |
| BTVhMb | FERRITIN | l2HVXz | NSCLC | rs72606621 | A | G | A | G | 0.0214 | -0.000340719 | 0.2642 | 0.338351 | 0.98658 | 0.0202567 | 3.73E-08 | 0.00388866 | 30.28500161 | 0.000178053 |
| BTVhMb | FERRITIN | l2HVXz | NSCLC | rs72775768 | T | C | T | C | -0.0202 | -0.016709 | 0.2561 | 0.253384 | 0.447734 | 0.0220087 | 1.99E-07 | 0.003884414 | 27.04280381 | 0.000155474 |
| BTVhMb | FERRITIN | l2HVXz | NSCLC | rs72798422 | C | T | C | T | 0.0466 | 0.0671725 | 0.0283 | 0.0347674 | 0.202186 | 0.0526699 | 2.91E-06 | 0.009963498 | 21.87500482 | 0.000119432 |
| BTVhMb | FERRITIN | l2HVXz | NSCLC | rs72830456 | A | G | A | G | 0.0355 | -0.0282319 | 0.0599 | 0.0582155 | 0.485071 | 0.040437 | 5.85E-07 | 0.007105536 | 24.96105963 | 0.000141934 |
| BTVhMb | FERRITIN | l2HVXz | NSCLC | rs73034900 | C | T | C | T | 0.0179 | 0.0160941 | 0.3922 | 0.322298 | 0.435439 | 0.0206356 | 7.15E-07 | 0.003610877 | 24.57427263 | 0.000152758 |
| BTVhMb | FERRITIN | l2HVXz | NSCLC | rs730953 | A | C | A | C | -0.0163 | -0.0048399 | 0.3714 | 0.418982 | 0.80289 | 0.0193899 | 4.60E-06 | 0.003557205 | 20.99700834 | 0.000124057 |
| BTVhMb | FERRITIN | l2HVXz | NSCLC | rs735831 | G | T | G | T | 0.0486 | -0.0754066 | 0.0535 | 0.0308862 | 0.167788 | 0.0546684 | 2.64E-10 | 0.007691618 | 39.9242829 | 0.000239209 |
| BTVhMb | FERRITIN | l2HVXz | NSCLC | rs7358798 | A | C | A | C | 0.0178 | 0.00690315 | 0.3028 | 0.380224 | 0.725857 | 0.0196872 | 5.29E-06 | 0.003909557 | 20.72931284 | 0.000133778 |
| BTVhMb | FERRITIN | l2HVXz | NSCLC | rs7435318 | G | A | G | A | 0.0161 | 0.00401048 | 0.3703 | 0.281281 | 0.849743 | 0.0211694 | 7.95E-06 | 0.003604591 | 19.94985594 | 0.000120884 |
| BTVhMb | FERRITIN | l2HVXz | NSCLC | rs7478136 | G | C | G | C | 0.0202 | -0.012881 | 0.2685 | 0.276166 | 0.545319 | 0.0212983 | 1.78E-07 | 0.003869022 | 27.25839876 | 0.000160284 |
| BTVhMb | FERRITIN | l2HVXz | NSCLC | rs7503385 | T | G | T | G | 0.0188 | 0.0314166 | 0.4315 | 0.544172 | 0.102556 | 0.0192434 | 6.10E-08 | 0.003471296 | 29.33137368 | 0.000173403 |
| BTVhMb | FERRITIN | l2HVXz | NSCLC | rs75848318 | G | T | G | T | 0.0463 | 0.0180623 | 0.0355 | 0.0158781 | 0.812178 | 0.0760141 | 6.95E-06 | 0.010299843 | 20.20695026 | 0.000146799 |
| BTVhMb | FERRITIN | l2HVXz | NSCLC | rs75939744 | A | G | A | G | 0.0286 | -0.00693623 | 0.0823 | 0.141736 | 0.798762 | 0.0272063 | 3.02E-06 | 0.006124912 | 21.80380853 | 0.000123556 |
| BTVhMb | FERRITIN | l2HVXz | NSCLC | rs7596205 | A | G | A | G | 0.0843 | 0.00878675 | 0.1021 | 0.0969603 | 0.784784 | 0.0321754 | 5.62E-49 | 0.005731075 | 216.3629855 | 0.001302983 |
| BTVhMb | FERRITIN | l2HVXz | NSCLC | rs75965181 | A | T | A | T | -0.1191 | -0.099381 | 0.0283 | 0.0122551 | 0.254947 | 0.0872976 | 3.70E-26 | 0.01125737 | 111.9308088 | 0.000780139 |
| BTVhMb | FERRITIN | l2HVXz | NSCLC | rs77178111 | T | C | T | C | 0.0232 | 0.000195767 | 0.1672 | 0.118513 | 0.9947 | 0.0294728 | 4.90E-07 | 0.004612154 | 25.3027876 | 0.000149894 |
| BTVhMb | FERRITIN | l2HVXz | NSCLC | rs7719875 | G | A | G | A | -0.0174 | -0.0334724 | 0.4187 | 0.376791 | 0.0908929 | 0.0197979 | 8.60E-07 | 0.003535698 | 24.21855185 | 0.000147378 |
| BTVhMb | FERRITIN | l2HVXz | NSCLC | rs77805826 | T | C | T | C | 0.0249 | -0.0244168 | 0.1563 | 0.0999056 | 0.440653 | 0.0316652 | 3.82E-07 | 0.00490378 | 25.78319529 | 0.000163522 |
| BTVhMb | FERRITIN | l2HVXz | NSCLC | rs78180894 | C | G | C | G | 0.0383 | -0.154731 | 0.0571 | 0.0179541 | 0.0331207 | 0.0726226 | 9.54E-08 | 0.007178637 | 28.46515558 | 0.000157954 |
| BTVhMb | FERRITIN | l2HVXz | NSCLC | rs7865362 | T | C | T | C | 0.0246 | -0.0262006 | 0.3458 | 0.329375 | 0.198414 | 0.0203724 | 1.03E-11 | 0.003616452 | 46.27056512 | 0.000273801 |
| BTVhMb | FERRITIN | l2HVXz | NSCLC | rs79092958 | C | T | C | T | 0.0407 | 0.0496519 | 0.0451 | 0.0398415 | 0.31134 | 0.0490431 | 6.32E-07 | 0.008170766 | 24.81208569 | 0.000142677 |
| BTVhMb | FERRITIN | l2HVXz | NSCLC | rs79242214 | A | G | A | G | 0.0465 | 0.0193288 | 0.028 | 0.0236482 | 0.752613 | 0.0613232 | 5.81E-06 | 0.010257681 | 20.54979661 | 0.000117696 |
| BTVhMb | FERRITIN | l2HVXz | NSCLC | rs79496834 | T | C | T | C | 0.0482 | -0.0674735 | 0.0459 | 0.0286102 | 0.238403 | 0.05723 | 8.86E-08 | 0.009011576 | 28.60833463 | 0.000203484 |
| BTVhMb | FERRITIN | l2HVXz | NSCLC | rs7959071 | G | C | G | C | 0.0168 | -0.000462938 | 0.4522 | 0.466514 | 0.980734 | 0.0191703 | 1.20E-06 | 0.003459896 | 23.57721339 | 0.00013983 |
| BTVhMb | FERRITIN | l2HVXz | NSCLC | rs79787985 | T | A | T | A | 0.0279 | -0.013455 | 0.0957 | 0.0863996 | 0.693921 | 0.0341896 | 5.82E-06 | 0.006155102 | 20.54650215 | 0.00013473 |
| BTVhMb | FERRITIN | l2HVXz | NSCLC | rs8075680 | T | C | T | C | -0.0163 | -0.0360745 | 0.3986 | 0.330185 | 0.0756014 | 0.0203031 | 3.70E-06 | 0.003522375 | 21.41430721 | 0.000127381 |
| BTVhMb | FERRITIN | l2HVXz | NSCLC | rs815271 | T | C | T | C | -0.0224 | -0.0168453 | 0.1752 | 0.189997 | 0.491561 | 0.0244905 | 6.55E-07 | 0.004503189 | 24.74318983 | 0.000145014 |
| BTVhMb | FERRITIN | l2HVXz | NSCLC | rs8177259 | C | T | C | T | -0.0168 | -0.00876962 | 0.2701 | 0.305102 | 0.671327 | 0.0206671 | 8.39E-06 | 0.003771059 | 19.8468647 | 0.000111285 |
| BTVhMb | FERRITIN | l2HVXz | NSCLC | rs820037 | A | G | A | G | 0.0178 | 0.0180666 | 0.2803 | 0.364534 | 0.365956 | 0.0199835 | 4.37E-06 | 0.003875495 | 21.09529693 | 0.000127833 |
| BTVhMb | FERRITIN | l2HVXz | NSCLC | rs830553 | G | T | G | T | 0.0158 | 0.00274496 | 0.46 | 0.503225 | 0.886191 | 0.0191786 | 5.49E-06 | 0.003476244 | 20.65825668 | 0.000124021 |
| BTVhMb | FERRITIN | l2HVXz | NSCLC | rs838112 | T | C | T | C | 0.0167 | -0.00251583 | 0.4955 | 0.424815 | 0.896408 | 0.0193228 | 1.37E-06 | 0.003458044 | 23.32232873 | 0.000139434 |
| BTVhMb | FERRITIN | l2HVXz | NSCLC | rs855791 | A | G | A | G | -0.0444 | 0.0302434 | 0.406 | 0.346069 | 0.130875 | 0.0200199 | 6.14E-37 | 0.003496875 | 161.2151025 | 0.000950842 |
| BTVhMb | FERRITIN | l2HVXz | NSCLC | rs859788 | G | A | G | A | -0.0188 | -0.00792559 | 0.4544 | 0.472948 | 0.678983 | 0.0191508 | 4.58E-08 | 0.003438884 | 29.88688452 | 0.00017525 |
| BTVhMb | FERRITIN | l2HVXz | NSCLC | rs9346719 | G | A | G | A | -0.0225 | -0.0311504 | 0.174 | 0.200406 | 0.189698 | 0.0237522 | 4.73E-07 | 0.004466986 | 25.37089883 | 0.000145521 |
| BTVhMb | FERRITIN | l2HVXz | NSCLC | rs9512463 | T | C | T | C | 0.0247 | -0.0121661 | 0.2237 | 0.257972 | 0.581692 | 0.0220834 | 3.12E-09 | 0.004168666 | 35.10748397 | 0.000211894 |
| BTVhMb | FERRITIN | l2HVXz | NSCLC | rs970079 | G | A | G | A | -0.0199 | -0.0252623 | 0.4602 | 0.343694 | 0.212238 | 0.0202514 | 8.31E-09 | 0.003453634 | 33.20118086 | 0.00019675 |
| BTVhMb | FERRITIN | l2HVXz | NSCLC | rs9921222 | C | T | C | T | 0.0248 | 0.0246452 | 0.4287 | 0.522667 | 0.199006 | 0.0191882 | 1.09E-12 | 0.003483813 | 50.67499224 | 0.000301267 |
| BTVhMb | FERRITIN | l2HVXz | NSCLC | rs996347 | C | T | C | T | 0.0486 | 0.0190598 | 0.3408 | 0.344305 | 0.343825 | 0.0201342 | 2.99E-41 | 0.003612802 | 180.960679 | 0.001061254 |

| **Supplementary Table 7 Statistical characteristics of eligible instrumental variables in forward MR analysis.** | | | | | | | | | | | | | | | | | | |
| --- | --- | --- | --- | --- | --- | --- | --- | --- | --- | --- | --- | --- | --- | --- | --- | --- | --- | --- |
| **SERUM AND NSCLC** | | | | | | | | | | | | | | | | | | |
| **id.exposure** | **exposure** | **id.outcome** | **outcome** | **SNP** | **effect_allele.exposure** | **other_allele.exposure** | **effect_allele.outcome** | **other_allele.outcome** | **beta.exposure** | **beta.outcome** | **eaf.exposure** | **eaf.outcome** | **pval.outcome** | **se.outcome** | **pval.exposure** | **se.exposure** | **F** | **R2** |
| IJ0ZJz | SERUM | eTy19K | NSCLC | rs10027255 | T | C | T | C | -0.0197 | 0.0209933 | 0.3055 | 0.356525 | 0.525412 | 0.033059 | 8.17E-06 | 0.004416367 | 19.89766616 | 0.000164682 |
| IJ0ZJz | SERUM | eTy19K | NSCLC | rs10138634 | G | A | G | A | 0.0187 | 0.0302324 | 0.3719 | 0.305269 | 0.374408 | 0.0340361 | 6.31E-06 | 0.004141083 | 20.39178966 | 0.000163368 |
| IJ0ZJz | SERUM | eTy19K | NSCLC | rs10159312 | T | C | T | C | -0.0258 | -0.0406523 | 0.1573 | 0.0845973 | 0.467313 | 0.0559289 | 3.48E-06 | 0.005560054 | 21.53185234 | 0.00017647 |
| IJ0ZJz | SERUM | eTy19K | NSCLC | rs10261327 | T | C | T | C | 0.0189 | 0.0101572 | 0.437 | 0.387191 | 0.754969 | 0.0325454 | 2.92E-06 | 0.004041598 | 21.8684187 | 0.000175769 |
| IJ0ZJz | SERUM | eTy19K | NSCLC | rs10421599 | A | G | A | G | -0.0295 | -0.00164346 | 0.2051 | 0.219 | 0.965776 | 0.0383034 | 1.46E-09 | 0.004877064 | 36.58702714 | 0.000283761 |
| IJ0ZJz | SERUM | eTy19K | NSCLC | rs10822143 | C | T | C | T | -0.0235 | -0.0425585 | 0.4548 | 0.472659 | 0.179551 | 0.0317095 | 4.62E-09 | 0.004010026 | 34.34324654 | 0.000273868 |
| IJ0ZJz | SERUM | eTy19K | NSCLC | rs10824743 | C | A | C | A | -0.0206 | -0.00646947 | 0.247 | 0.208939 | 0.867793 | 0.0388643 | 9.36E-06 | 0.004648588 | 19.63777714 | 0.000157854 |
| IJ0ZJz | SERUM | eTy19K | NSCLC | rs10922469 | A | G | A | G | 0.0468 | 0.0158328 | 0.0419 | 0.0361359 | 0.852937 | 0.0854103 | 6.75E-06 | 0.010396712 | 20.26281031 | 0.000175852 |
| IJ0ZJz | SERUM | eTy19K | NSCLC | rs114081780 | G | A | G | A | -0.0559 | 0.0185273 | 0.0252 | 0.0138382 | 0.888157 | 0.131739 | 3.15E-06 | 0.011993678 | 21.72295217 | 0.000153522 |
| IJ0ZJz | SERUM | eTy19K | NSCLC | rs114165349 | C | G | C | G | 0.0721 | -0.178334 | 0.0133 | 0.037648 | 0.0368375 | 0.0854273 | 4.83E-06 | 0.015769768 | 20.90354391 | 0.000136439 |
| IJ0ZJz | SERUM | eTy19K | NSCLC | rs114708114 | T | C | T | C | 0.1412 | -0.0510408 | 0.0282 | 0.00399419 | 0.845095 | 0.261238 | 7.52E-32 | 0.012022457 | 137.9376836 | 0.001092762 |
| IJ0ZJz | SERUM | eTy19K | NSCLC | rs114786620 | T | C | T | C | -0.0608 | -0.144526 | 0.0372 | 0.0306953 | 0.125715 | 0.094386 | 2.46E-07 | 0.011781299 | 26.63304373 | 0.000264799 |
| IJ0ZJz | SERUM | eTy19K | NSCLC | rs115083160 | A | G | A | G | -0.0586 | 0.0505884 | 0.0205 | 0.0454451 | 0.505323 | 0.0759429 | 5.56E-06 | 0.012900482 | 20.63399675 | 0.000137906 |
| IJ0ZJz | SERUM | eTy19K | NSCLC | rs116009877 | A | G | A | G | 0.2599 | 0.0902353 | 0.0647 | 0.0356033 | 0.296115 | 0.086366 | 1.00E-200 | 0.007875124 | 1089.175233 | 0.008175188 |
| IJ0ZJz | SERUM | eTy19K | NSCLC | rs116169498 | G | C | G | C | 0.1592 | -0.179795 | 0.0144 | 0.0117361 | 0.215723 | 0.145232 | 6.24E-21 | 0.016961669 | 88.09453328 | 0.000719415 |
| IJ0ZJz | SERUM | eTy19K | NSCLC | rs116583876 | A | G | A | G | -0.0374 | 0.162551 | 0.0619 | 0.0280424 | 0.0811727 | 0.09321 | 6.52E-06 | 0.008294918 | 20.32914016 | 0.000162447 |
| IJ0ZJz | SERUM | eTy19K | NSCLC | rs117084494 | A | G | A | G | -0.0859 | 0.0182679 | 0.0106 | 0.0084037 | 0.915399 | 0.171965 | 8.24E-06 | 0.019265051 | 19.88135598 | 0.000154773 |
| IJ0ZJz | SERUM | eTy19K | NSCLC | rs117157488 | A | G | A | G | -0.0429 | 0.0387343 | 0.0453 | 0.0280697 | 0.679338 | 0.0937044 | 5.46E-06 | 0.009436267 | 20.66874669 | 0.000159188 |
| IJ0ZJz | SERUM | eTy19K | NSCLC | rs117718169 | T | C | T | C | -0.0718 | 0.199773 | 0.0326 | 0.0190481 | 0.0809189 | 0.114459 | 3.28E-11 | 0.010823982 | 44.002238 | 0.000325164 |
| IJ0ZJz | SERUM | eTy19K | NSCLC | rs117753190 | G | C | G | C | 0.0727 | -0.0544958 | 0.0283 | 0.0105402 | 0.71554 | 0.149539 | 3.37E-10 | 0.011575099 | 39.44749169 | 0.000290682 |
| IJ0ZJz | SERUM | eTy19K | NSCLC | rs12034310 | G | A | G | A | 0.0179 | -0.0116111 | 0.4938 | 0.541342 | 0.71409 | 0.0316924 | 7.60E-06 | 0.00399897 | 20.03594218 | 0.00016018 |
| IJ0ZJz | SERUM | eTy19K | NSCLC | rs12143966 | A | G | A | G | -0.0205 | 0.008311 | 0.3796 | 0.391118 | 0.796632 | 0.0322496 | 6.35E-07 | 0.004116254 | 24.80295302 | 0.000197941 |
| IJ0ZJz | SERUM | eTy19K | NSCLC | rs12206204 | T | C | T | C | 0.1732 | -0.144172 | 0.0107 | 0.0157139 | 0.227422 | 0.119444 | 2.94E-22 | 0.017850942 | 94.13984904 | 0.000635093 |
| IJ0ZJz | SERUM | eTy19K | NSCLC | rs1223763 | T | G | T | G | 0.0234 | 0.0511174 | 0.2304 | 0.19907 | 0.195363 | 0.0394767 | 1.74E-06 | 0.004893871 | 22.86265591 | 0.000194182 |
| IJ0ZJz | SERUM | eTy19K | NSCLC | rs12407066 | A | C | A | C | -0.0226 | 0.00788472 | 0.2551 | 0.281147 | 0.822355 | 0.0351186 | 8.95E-07 | 0.004599645 | 24.1417223 | 0.000194113 |
| IJ0ZJz | SERUM | eTy19K | NSCLC | rs12633819 | G | A | G | A | 0.0258 | -0.0251154 | 0.3879 | 0.276921 | 0.473583 | 0.0350449 | 5.34E-10 | 0.004155411 | 38.54885135 | 0.000316091 |
| IJ0ZJz | SERUM | eTy19K | NSCLC | rs12718598 | C | T | C | T | 0.0266 | 0.0584252 | 0.4286 | 0.398448 | 0.0698538 | 0.0322282 | 3.69E-11 | 0.004020544 | 43.77172224 | 0.000346566 |
| IJ0ZJz | SERUM | eTy19K | NSCLC | rs12975762 | G | A | G | A | -0.0289 | -0.00724696 | 0.328 | 0.232222 | 0.846469 | 0.0374277 | 1.70E-11 | 0.004294389 | 45.28901989 | 0.000368187 |
| IJ0ZJz | SERUM | eTy19K | NSCLC | rs13007705 | T | C | T | C | 0.0289 | 0.0038828 | 0.3745 | 0.38328 | 0.904682 | 0.0324245 | 2.01E-12 | 0.004108739 | 49.4741768 | 0.000391295 |
| IJ0ZJz | SERUM | eTy19K | NSCLC | rs13008165 | T | A | T | A | 0.0192 | -8.67764E-05 | 0.3223 | 0.24525 | 0.998094 | 0.0363238 | 6.57E-06 | 0.004259885 | 20.31452665 | 0.000161039 |
| IJ0ZJz | SERUM | eTy19K | NSCLC | rs13209546 | C | T | C | T | -0.0626 | -0.0271555 | 0.0192 | 0.0118355 | 0.84694 | 0.140685 | 7.24E-06 | 0.013952945 | 20.12875444 | 0.000147591 |
| IJ0ZJz | SERUM | eTy19K | NSCLC | rs13266821 | T | C | T | C | 0.0248 | 0.025065 | 0.1701 | 0.115643 | 0.610038 | 0.0491452 | 6.06E-06 | 0.005481529 | 20.4691557 | 0.000173645 |
| IJ0ZJz | SERUM | eTy19K | NSCLC | rs140047638 | C | T | C | T | 0.1002 | -0.192951 | 0.0054 | 0.00544278 | 0.352951 | 0.207724 | 4.64E-06 | 0.02187563 | 20.98042612 | 0.000107847 |
| IJ0ZJz | SERUM | eTy19K | NSCLC | rs140393761 | G | A | G | A | 0.1057 | 0.159628 | 0.0199 | 0.0117425 | 0.268273 | 0.144192 | 3.34E-13 | 0.014519376 | 52.99733977 | 0.000435816 |
| IJ0ZJz | SERUM | eTy19K | NSCLC | rs144063236 | T | C | T | C | 0.1032 | 0.0668698 | 0.006 | 0.042338 | 0.403083 | 0.0799755 | 6.03E-06 | 0.022804943 | 20.47865419 | 0.000127036 |
| IJ0ZJz | SERUM | eTy19K | NSCLC | rs145096438 | C | G | C | G | -0.0754 | 0.0188048 | 0.0135 | 0.0235033 | 0.862047 | 0.108217 | 5.51E-06 | 0.016591962 | 20.65129083 | 0.000151427 |
| IJ0ZJz | SERUM | eTy19K | NSCLC | rs145496147 | T | C | T | C | 0.1634 | 0.0821744 | 0.0081 | 0.0205873 | 0.466455 | 0.112837 | 2.25E-15 | 0.020613808 | 62.83297906 | 0.000429029 |
| IJ0ZJz | SERUM | eTy19K | NSCLC | rs146284844 | A | G | A | G | 0.0545 | -0.355146 | 0.03 | 0.00749108 | 0.0443466 | 0.176619 | 7.85E-06 | 0.012194482 | 19.97405726 | 0.000172869 |
| IJ0ZJz | SERUM | eTy19K | NSCLC | rs148978261 | G | C | G | C | 0.0813 | -0.166531 | 0.0153 | 0.00331163 | 0.535945 | 0.26905 | 6.69E-06 | 0.018053347 | 20.27989171 | 0.000199162 |
| IJ0ZJz | SERUM | eTy19K | NSCLC | rs149830227 | T | C | T | C | 0.0566 | -0.0949425 | 0.028 | 0.00839348 | 0.589624 | 0.176021 | 8.21E-06 | 0.012691626 | 19.88833062 | 0.000174376 |
| IJ0ZJz | SERUM | eTy19K | NSCLC | rs16871037 | G | C | G | C | 0.0746 | 0.119509 | 0.0151 | 0.00281888 | 0.65711 | 0.26922 | 1.17E-06 | 0.015347736 | 23.62593553 | 0.00016553 |
| IJ0ZJz | SERUM | eTy19K | NSCLC | rs17019886 | G | T | G | T | 0.0497 | -0.0346999 | 0.0348 | 0.029397 | 0.712756 | 0.0942529 | 4.39E-06 | 0.01082315 | 21.08654478 | 0.000165936 |
| IJ0ZJz | SERUM | eTy19K | NSCLC | rs17767386 | C | T | C | T | -0.0219 | 0.0504379 | 0.2159 | 0.173678 | 0.222564 | 0.0413514 | 5.29E-06 | 0.004810073 | 20.72931231 | 0.000162384 |
| IJ0ZJz | SERUM | eTy19K | NSCLC | rs1958078 | A | C | A | C | -0.0321 | -0.0425542 | 0.133 | 0.080573 | 0.464802 | 0.0582166 | 2.48E-08 | 0.005758189 | 31.07693888 | 0.000237635 |
| IJ0ZJz | SERUM | eTy19K | NSCLC | rs2042919 | G | A | G | A | -0.0225 | -0.00644883 | 0.2494 | 0.207576 | 0.867629 | 0.0386921 | 1.01E-06 | 0.004601528 | 23.90897171 | 0.00018954 |
| IJ0ZJz | SERUM | eTy19K | NSCLC | rs2043192 | G | A | G | A | -0.0192 | -0.0369377 | 0.4445 | 0.448287 | 0.242759 | 0.0316215 | 2.58E-06 | 0.004083626 | 22.10601889 | 0.000182049 |
| IJ0ZJz | SERUM | eTy19K | NSCLC | rs2158799 | C | G | C | G | -0.0211 | 0.00370784 | 0.403 | 0.456575 | 0.906982 | 0.0317329 | 2.73E-07 | 0.004104106 | 26.43186286 | 0.000214227 |
| IJ0ZJz | SERUM | eTy19K | NSCLC | rs2613522 | G | A | G | A | -0.0212 | -0.0344462 | 0.2834 | 0.265983 | 0.337408 | 0.0359077 | 2.09E-06 | 0.004468312 | 22.51047927 | 0.000182549 |
| IJ0ZJz | SERUM | eTy19K | NSCLC | rs28929474 | T | C | T | C | 0.0922 | -0.157807 | 0.0082 | 0.0197606 | 0.162126 | 0.112884 | 3.66E-07 | 0.018128761 | 25.86578094 | 0.000138271 |
| IJ0ZJz | SERUM | eTy19K | NSCLC | rs41272158 | T | C | T | C | 0.04 | 0.078518 | 0.0675 | 0.0680325 | 0.225954 | 0.0648455 | 4.31E-07 | 0.00791338 | 25.55029628 | 0.00020142 |
| IJ0ZJz | SERUM | eTy19K | NSCLC | rs4774514 | T | C | T | C | -0.0491 | -0.0993066 | 0.0665 | 0.039001 | 0.231952 | 0.0830778 | 2.87E-09 | 0.008267571 | 35.27013466 | 0.000299315 |
| IJ0ZJz | SERUM | eTy19K | NSCLC | rs4817984 | A | C | A | C | 0.0213 | 0.0339361 | 0.3116 | 0.260346 | 0.344123 | 0.0358714 | 1.19E-06 | 0.004385156 | 23.59332288 | 0.000194638 |
| IJ0ZJz | SERUM | eTy19K | NSCLC | rs4854760 | G | A | G | A | 0.053 | 0.00751035 | 0.2639 | 0.295816 | 0.827171 | 0.0343988 | 5.67E-33 | 0.004430974 | 143.0715677 | 0.001091335 |
| IJ0ZJz | SERUM | eTy19K | NSCLC | rs55709272 | C | T | C | T | -0.0202 | 0.0465088 | 0.489 | 0.322305 | 0.169787 | 0.0338766 | 4.16E-07 | 0.003990922 | 25.61865111 | 0.000203921 |
| IJ0ZJz | SERUM | eTy19K | NSCLC | rs563027675 | G | C | G | C | -0.0718 | -0.0169601 | 0.0257 | 0.0303508 | 0.855008 | 0.0928143 | 3.13E-08 | 0.012974329 | 30.62521001 | 0.000258169 |
| IJ0ZJz | SERUM | eTy19K | NSCLC | rs563076 | C | A | C | A | 0.0189 | 0.00490854 | 0.381 | 0.464525 | 0.876543 | 0.031596 | 4.16E-06 | 0.004105818 | 21.18967129 | 0.000168488 |
| IJ0ZJz | SERUM | eTy19K | NSCLC | rs56912861 | A | G | A | G | -0.0337 | -0.0344828 | 0.3658 | 0.470138 | 0.275925 | 0.0316495 | 1.02E-15 | 0.004199676 | 64.39145357 | 0.000526938 |
| IJ0ZJz | SERUM | eTy19K | NSCLC | rs58658771 | A | T | A | T | 0.0246 | -0.0300806 | 0.1654 | 0.26486 | 0.400971 | 0.035815 | 3.65E-06 | 0.005312742 | 21.44039401 | 0.000167076 |
| IJ0ZJz | SERUM | eTy19K | NSCLC | rs7216369 | T | C | T | C | -0.0338 | -0.0875392 | 0.0923 | 0.0438683 | 0.250279 | 0.0761429 | 1.88E-06 | 0.00709203 | 22.71392945 | 0.000191429 |
| IJ0ZJz | SERUM | eTy19K | NSCLC | rs73181000 | A | G | A | G | 0.0483 | 0.0417724 | 0.0519 | 0.0466202 | 0.583296 | 0.0761471 | 8.35E-07 | 0.009803117 | 24.27537176 | 0.000229586 |
| IJ0ZJz | SERUM | eTy19K | NSCLC | rs7385804 | C | A | C | A | -0.0572 | 0.00535565 | 0.3516 | 0.448509 | 0.866057 | 0.0317524 | 9.42E-43 | 0.004173527 | 187.8389287 | 0.001491811 |
| IJ0ZJz | SERUM | eTy19K | NSCLC | rs739936 | T | C | T | C | -0.0254 | -0.0186821 | 0.2141 | 0.28894 | 0.593418 | 0.0349925 | 1.34E-07 | 0.004816728 | 27.80757955 | 0.000217111 |
| IJ0ZJz | SERUM | eTy19K | NSCLC | rs74293923 | C | T | C | T | -0.0635 | 0.0075753 | 0.0188 | 0.00997339 | 0.961362 | 0.15637 | 9.87E-06 | 0.014366509 | 19.53641859 | 0.000148762 |
| IJ0ZJz | SERUM | eTy19K | NSCLC | rs74338506 | A | G | A | G | -0.0649 | -0.0887089 | 0.0201 | 0.0223642 | 0.421646 | 0.110394 | 2.14E-06 | 0.013692754 | 22.4650703 | 0.000165919 |
| IJ0ZJz | SERUM | eTy19K | NSCLC | rs7574777 | C | T | C | T | 0.0266 | -0.0671033 | 0.144 | 0.127604 | 0.156951 | 0.0474094 | 7.64E-06 | 0.005944092 | 20.02590852 | 0.000174433 |
| IJ0ZJz | SERUM | eTy19K | NSCLC | rs77034634 | T | C | T | C | 0.0369 | -0.0178505 | 0.0607 | 0.0961939 | 0.742308 | 0.0542899 | 4.13E-06 | 0.008013498 | 21.20354453 | 0.000155266 |
| IJ0ZJz | SERUM | eTy19K | NSCLC | rs77283192 | C | T | C | T | 0.0512 | 0.00943237 | 0.033 | 0.0274627 | 0.920725 | 0.0947779 | 8.15E-06 | 0.011476719 | 19.90235302 | 0.000167306 |
| IJ0ZJz | SERUM | eTy19K | NSCLC | rs7837764 | C | G | C | G | 0.0223 | 0.0333165 | 0.4021 | 0.378465 | 0.304722 | 0.0324609 | 4.98E-08 | 0.004090225 | 29.72455273 | 0.000239113 |
| IJ0ZJz | SERUM | eTy19K | NSCLC | rs9265878 | A | G | A | G | -0.0265 | -0.0120178 | 0.2404 | 0.279367 | 0.731161 | 0.034978 | 9.33E-09 | 0.004614731 | 32.97607537 | 0.000256473 |
| IJ0ZJz | SERUM | eTy19K | NSCLC | rs9295767 | A | G | A | G | -0.0649 | 0.0734517 | 0.0531 | 0.0316882 | 0.411059 | 0.0893539 | 4.90E-12 | 0.009394301 | 47.726595 | 0.000423563 |
| IJ0ZJz | SERUM | eTy19K | NSCLC | rs9399136 | C | T | C | T | 0.0574 | -0.0473938 | 0.2695 | 0.343836 | 0.157217 | 0.0335058 | 1.08E-36 | 0.004536556 | 160.0925906 | 0.001297277 |
| IJ0ZJz | SERUM | eTy19K | NSCLC | rs983828 | G | A | G | A | -0.0223 | -0.00961179 | 0.2625 | 0.350169 | 0.77267 | 0.0332721 | 9.56E-07 | 0.004550569 | 24.01476373 | 0.000192544 |

| **Supplementary Table 8 Statistical characteristics of eligible instrumental variables in forward MR analysis.** | | | | | | | | | | | | | | | | | | |
| --- | --- | --- | --- | --- | --- | --- | --- | --- | --- | --- | --- | --- | --- | --- | --- | --- | --- | --- |
| **TIBC AND NSCLC** | | | | | | | | | | | | | | | | | | |
| **id.exposure** | **exposure** | **id.outcome** | **outcome** | **SNP** | **effect_allele.exposure** | **other_allele.exposure** | **effect_allele.outcome** | **other_allele.outcome** | **beta.exposure** | **beta.outcome** | **eaf.exposure** | **eaf.outcome** | **pval.outcome** | **se.outcome** | **pval.exposure** | **se.exposure** | **F** | **R2** |
| YgJTec | TIBC | b7vI1p | NSCLC | rs10045483 | G | A | G | A | -0.0755 | -0.396647 | 0.0196 | 0.00410362 | 0.107605 | 0.24651 | 4.12E-06 | 1.64E-02 | 2.12E+01 | 2.19E-04 |
| YgJTec | TIBC | b7vI1p | NSCLC | rs1106735 | G | A | G | A | 0.0791 | -0.00461101 | 0.1078 | 0.152287 | 0.916046 | 0.0437411 | 2.92E-27 | 7.31E-03 | 1.17E+02 | 1.20E-03 |
| YgJTec | TIBC | b7vI1p | NSCLC | rs11125072 | A | T | A | T | 0.0259 | -0.0488367 | 0.4578 | 0.456355 | 0.125366 | 0.0318646 | 3.25E-08 | 4.69E-03 | 3.06E+01 | 3.33E-04 |
| YgJTec | TIBC | b7vI1p | NSCLC | rs111302943 | A | G | A | G | 0.0328 | 0.0484615 | 0.1258 | 0.225606 | 0.202996 | 0.0380669 | 3.54E-06 | 7.07E-03 | 2.15E+01 | 2.37E-04 |
| YgJTec | TIBC | b7vI1p | NSCLC | rs112466891 | C | A | C | A | 0.0453 | 0.0620558 | 0.1791 | 0.243674 | 0.0955939 | 0.0372348 | 1.63E-13 | 6.14E-03 | 5.44E+01 | 6.03E-04 |
| YgJTec | TIBC | b7vI1p | NSCLC | rs112727702 | T | G | T | G | 0.0433 | 0.0429512 | 0.1963 | 0.215052 | 0.264212 | 0.0384698 | 2.08E-14 | 5.66E-03 | 5.85E+01 | 5.92E-04 |
| YgJTec | TIBC | b7vI1p | NSCLC | rs115421711 | G | A | G | A | -0.0598 | 0.136133 | 0.0402 | 0.0296748 | 0.136357 | 0.0913952 | 3.66E-07 | 1.18E-02 | 2.59E+01 | 2.76E-04 |
| YgJTec | TIBC | b7vI1p | NSCLC | rs116272812 | C | T | C | T | -0.2414 | -0.0028075 | 0.1181 | 0.128953 | 0.952391 | 0.0470233 | 1.00E-200 | 7.03E-03 | 1.18E+03 | 1.21E-02 |
| YgJTec | TIBC | b7vI1p | NSCLC | rs118030488 | T | G | T | G | -0.0808 | 0.0654486 | 0.0253 | 0.029747 | 0.498764 | 0.0967551 | 4.08E-07 | 1.60E-02 | 2.57E+01 | 3.22E-04 |
| YgJTec | TIBC | b7vI1p | NSCLC | rs118066080 | T | C | T | C | 0.0769 | 0.106538 | 0.0206 | 0.017512 | 0.387201 | 0.123207 | 8.78E-06 | 1.73E-02 | 1.98E+01 | 2.39E-04 |
| YgJTec | TIBC | b7vI1p | NSCLC | rs11918732 | T | C | T | C | 0.0223 | 0.0434008 | 0.361 | 0.355341 | 0.188266 | 0.0329862 | 4.61E-06 | 4.87E-03 | 2.10E+01 | 2.29E-04 |
| YgJTec | TIBC | b7vI1p | NSCLC | rs1198439 | A | T | A | T | -0.0371 | -0.028277 | 0.1232 | 0.112128 | 0.572191 | 0.0500631 | 2.14E-07 | 7.15E-03 | 2.69E+01 | 2.97E-04 |
| YgJTec | TIBC | b7vI1p | NSCLC | rs12206077 | A | G | A | G | -0.0665 | -0.02933 | 0.2838 | 0.282544 | 0.402517 | 0.0350361 | 2.07E-38 | 5.13E-03 | 1.68E+02 | 1.80E-03 |
| YgJTec | TIBC | b7vI1p | NSCLC | rs12879801 | G | A | G | A | 0.0252 | -0.0182303 | 0.4676 | 0.510411 | 0.563953 | 0.0315961 | 6.11E-08 | 4.65E-03 | 2.93E+01 | 3.16E-04 |
| YgJTec | TIBC | b7vI1p | NSCLC | rs12976652 | C | T | C | T | -0.0446 | -0.0826726 | 0.0979 | 0.06336 | 0.191923 | 0.063355 | 2.55E-09 | 7.49E-03 | 3.55E+01 | 3.51E-04 |
| YgJTec | TIBC | b7vI1p | NSCLC | rs13008704 | C | T | C | T | -0.0369 | -0.0240849 | 0.4876 | 0.512601 | 0.445958 | 0.0316004 | 3.44E-15 | 4.69E-03 | 6.20E+01 | 6.80E-04 |
| YgJTec | TIBC | b7vI1p | NSCLC | rs13084306 | A | C | A | C | 0.1043 | -0.298178 | 0.0253 | 0.0118546 | 0.0458131 | 0.149305 | 1.32E-10 | 1.62E-02 | 4.13E+01 | 5.37E-04 |
| YgJTec | TIBC | b7vI1p | NSCLC | rs1435167 | T | A | T | A | 0.0242 | 0.0227859 | 0.3786 | 0.305855 | 0.503936 | 0.0340947 | 7.14E-07 | 4.88E-03 | 2.46E+01 | 2.76E-04 |
| YgJTec | TIBC | b7vI1p | NSCLC | rs143530446 | T | C | T | C | -0.1591 | 0.0608636 | 0.0299 | 0.00920429 | 0.706767 | 0.161784 | 2.96E-26 | 1.50E-02 | 1.12E+02 | 1.47E-03 |
| YgJTec | TIBC | b7vI1p | NSCLC | rs143878994 | A | G | A | G | 0.0567 | -0.0275956 | 0.0451 | 0.0285859 | 0.76771 | 0.093427 | 8.26E-07 | 1.15E-02 | 2.43E+01 | 2.77E-04 |
| YgJTec | TIBC | b7vI1p | NSCLC | rs1672992 | C | T | C | T | -0.033 | -0.0927989 | 0.1513 | 0.140064 | 0.0413124 | 0.0454811 | 5.33E-07 | 6.58E-03 | 2.51E+01 | 2.80E-04 |
| YgJTec | TIBC | b7vI1p | NSCLC | rs1680695 | G | T | G | T | -0.0247 | 0.0353943 | 0.3473 | 0.343652 | 0.287511 | 0.0332779 | 3.68E-07 | 4.86E-03 | 2.59E+01 | 2.77E-04 |
| YgJTec | TIBC | b7vI1p | NSCLC | rs174547 | C | T | C | T | 0.0462 | -0.0700578 | 0.3883 | 0.414257 | 0.0282742 | 0.0319393 | 6.57E-22 | 4.80E-03 | 9.25E+01 | 1.01E-03 |
| YgJTec | TIBC | b7vI1p | NSCLC | rs17580 | A | T | A | T | 0.0762 | 0.120339 | 0.0333 | 0.00916819 | 0.456221 | 0.161511 | 1.19E-10 | 1.18E-02 | 4.15E+01 | 3.74E-04 |
| YgJTec | TIBC | b7vI1p | NSCLC | rs17767742 | G | C | G | C | 0.0305 | -0.0180991 | 0.2828 | 0.310032 | 0.594903 | 0.0340371 | 1.95E-09 | 5.08E-03 | 3.60E+01 | 3.77E-04 |
| YgJTec | TIBC | b7vI1p | NSCLC | rs192458696 | C | T | C | T | -0.1056 | -0.265959 | 0.0139 | 0.00300385 | 0.324734 | 0.270071 | 1.78E-07 | 2.02E-02 | 2.73E+01 | 3.06E-04 |
| YgJTec | TIBC | b7vI1p | NSCLC | rs1927693 | A | G | A | G | 0.0367 | -0.0742568 | 0.382 | 0.369552 | 0.0231937 | 0.0327089 | 2.02E-14 | 4.80E-03 | 5.85E+01 | 6.36E-04 |
| YgJTec | TIBC | b7vI1p | NSCLC | rs1930884 | T | C | T | C | -0.0239 | 0.0168681 | 0.289 | 0.212093 | 0.668092 | 0.039341 | 7.36E-06 | 5.33E-03 | 2.01E+01 | 2.35E-04 |
| YgJTec | TIBC | b7vI1p | NSCLC | rs199138 | A | G | A | G | 0.0807 | -0.0799501 | 0.0815 | 0.070731 | 0.200678 | 0.0624794 | 9.59E-21 | 8.64E-03 | 8.72E+01 | 9.75E-04 |
| YgJTec | TIBC | b7vI1p | NSCLC | rs2088102 | T | C | T | C | -0.0213 | -0.0226626 | 0.4962 | 0.516986 | 0.473218 | 0.0315964 | 4.17E-06 | 4.63E-03 | 2.12E+01 | 2.27E-04 |
| YgJTec | TIBC | b7vI1p | NSCLC | rs2236252 | T | C | T | C | 0.0359 | -0.0156238 | 0.1788 | 0.147756 | 0.725279 | 0.0444599 | 4.02E-09 | 6.10E-03 | 3.46E+01 | 3.78E-04 |
| YgJTec | TIBC | b7vI1p | NSCLC | rs2305268 | T | C | T | C | 0.0956 | 0.118076 | 0.016 | 0.0472369 | 0.122581 | 0.0764726 | 2.34E-07 | 1.85E-02 | 2.67E+01 | 2.88E-04 |
| YgJTec | TIBC | b7vI1p | NSCLC | rs28411900 | T | C | T | C | -0.0319 | 0.0455152 | 0.1141 | 0.166646 | 0.282495 | 0.0423502 | 6.64E-06 | 7.08E-03 | 2.03E+01 | 2.06E-04 |
| YgJTec | TIBC | b7vI1p | NSCLC | rs2875251 | C | T | C | T | -0.0257 | 0.0577574 | 0.2637 | 0.247248 | 0.114597 | 0.0366048 | 2.18E-06 | 5.43E-03 | 2.24E+01 | 2.56E-04 |
| YgJTec | TIBC | b7vI1p | NSCLC | rs35570672 | T | C | T | C | -0.0429 | -0.0698952 | 0.1877 | 0.25091 | 0.053889 | 0.0362578 | 1.26E-13 | 5.79E-03 | 5.49E+01 | 5.61E-04 |
| YgJTec | TIBC | b7vI1p | NSCLC | rs35769520 | G | A | G | A | 0.0264 | 0.0495667 | 0.4885 | 0.636144 | 0.128468 | 0.032606 | 1.34E-08 | 4.65E-03 | 3.23E+01 | 3.48E-04 |
| YgJTec | TIBC | b7vI1p | NSCLC | rs4671609 | G | A | G | A | -0.0219 | -0.0263766 | 0.3875 | 0.362316 | 0.419913 | 0.0327021 | 4.20E-06 | 4.76E-03 | 2.12E+01 | 2.28E-04 |
| YgJTec | TIBC | b7vI1p | NSCLC | rs469882 | C | A | C | A | -0.0367 | -0.00926007 | 0.2221 | 0.214527 | 0.811538 | 0.0388357 | 1.03E-10 | 5.68E-03 | 4.18E+01 | 4.65E-04 |
| YgJTec | TIBC | b7vI1p | NSCLC | rs4764934 | T | C | T | C | -0.0265 | 0.0665298 | 0.2013 | 0.282344 | 0.0582962 | 0.0351366 | 6.90E-06 | 5.89E-03 | 2.02E+01 | 2.26E-04 |
| YgJTec | TIBC | b7vI1p | NSCLC | rs4846335 | A | C | A | C | 0.0445 | 0.04709 | 0.1105 | 0.144664 | 0.295378 | 0.045002 | 2.87E-09 | 7.49E-03 | 3.53E+01 | 3.89E-04 |
| YgJTec | TIBC | b7vI1p | NSCLC | rs4849902 | C | T | C | T | 0.0241 | -0.021913 | 0.386 | 0.277874 | 0.536523 | 0.0354533 | 6.48E-07 | 4.84E-03 | 2.48E+01 | 2.75E-04 |
| YgJTec | TIBC | b7vI1p | NSCLC | rs4854737 | G | C | G | C | 0.1444 | -0.0370233 | 0.4575 | 0.362936 | 0.256903 | 0.0326558 | 1.00E-200 | 4.69E-03 | 9.47E+02 | 1.04E-02 |
| YgJTec | TIBC | b7vI1p | NSCLC | rs56195124 | A | G | A | G | 0.0882 | -0.0437394 | 0.0543 | 0.0321978 | 0.617528 | 0.0875915 | 3.45E-16 | 1.08E-02 | 6.65E+01 | 7.99E-04 |
| YgJTec | TIBC | b7vI1p | NSCLC | rs59950280 | A | G | A | G | 0.0328 | -0.0113471 | 0.3195 | 0.243016 | 0.75733 | 0.0367234 | 5.87E-11 | 5.01E-03 | 4.29E+01 | 4.68E-04 |
| YgJTec | TIBC | b7vI1p | NSCLC | rs6025 | T | C | T | C | -0.093 | 0.250939 | 0.0188 | 0.0202383 | 0.0271957 | 0.113614 | 1.76E-08 | 1.65E-02 | 3.17E+01 | 3.19E-04 |
| YgJTec | TIBC | b7vI1p | NSCLC | rs60721435 | C | T | C | T | 0.0372 | -0.000910403 | 0.1152 | 0.0857705 | 0.987072 | 0.0561864 | 8.41E-08 | 6.94E-03 | 2.87E+01 | 2.82E-04 |
| YgJTec | TIBC | b7vI1p | NSCLC | rs62183592 | T | C | T | C | 0.0496 | -0.0755994 | 0.077 | 0.0397071 | 0.343159 | 0.0797512 | 3.31E-08 | 8.98E-03 | 3.05E+01 | 3.50E-04 |
| YgJTec | TIBC | b7vI1p | NSCLC | rs637828 | C | A | C | A | -0.0221 | -0.0169958 | 0.4011 | 0.406345 | 0.595626 | 0.0320253 | 2.76E-06 | 4.71E-03 | 2.20E+01 | 2.35E-04 |
| YgJTec | TIBC | b7vI1p | NSCLC | rs715 | C | T | C | T | 0.0247 | 0.051515 | 0.2726 | 0.313735 | 0.133104 | 0.0342982 | 1.54E-06 | 5.14E-03 | 2.31E+01 | 2.42E-04 |
| YgJTec | TIBC | b7vI1p | NSCLC | rs722740 | C | T | C | T | 0.0236 | -0.00484402 | 0.3965 | 0.474941 | 0.878396 | 0.0316597 | 6.98E-07 | 4.76E-03 | 2.46E+01 | 2.67E-04 |
| YgJTec | TIBC | b7vI1p | NSCLC | rs72840508 | T | A | T | A | -0.1212 | 0.175349 | 0.034 | 0.0182779 | 0.136193 | 0.117674 | 3.40E-20 | 1.32E-02 | 8.47E+01 | 9.65E-04 |
| YgJTec | TIBC | b7vI1p | NSCLC | rs7297861 | C | T | C | T | 0.0479 | 0.0248633 | 0.0794 | 0.122286 | 0.600026 | 0.0474161 | 3.99E-09 | 8.14E-03 | 3.46E+01 | 3.35E-04 |
| YgJTec | TIBC | b7vI1p | NSCLC | rs7432894 | C | T | C | T | -0.0377 | 0.0207897 | 0.2955 | 0.272417 | 0.558074 | 0.0354953 | 1.52E-13 | 5.10E-03 | 5.45E+01 | 5.92E-04 |
| YgJTec | TIBC | b7vI1p | NSCLC | rs7500731 | G | A | G | A | 0.0229 | -0.012086 | 0.4891 | 0.303083 | 0.723248 | 0.0341296 | 9.45E-07 | 4.67E-03 | 2.40E+01 | 2.62E-04 |
| YgJTec | TIBC | b7vI1p | NSCLC | rs780093 | T | C | T | C | 0.0235 | -0.0105656 | 0.3416 | 0.352333 | 0.749918 | 0.0331471 | 1.10E-06 | 4.82E-03 | 2.37E+01 | 2.48E-04 |
| YgJTec | TIBC | b7vI1p | NSCLC | rs79364962 | A | G | A | G | 0.0364 | -0.0135064 | 0.1005 | 0.0635945 | 0.835546 | 0.0650617 | 8.50E-06 | 8.18E-03 | 1.98E+01 | 2.40E-04 |
| YgJTec | TIBC | b7vI1p | NSCLC | rs8062982 | C | G | C | G | 0.0255 | -0.00417997 | 0.3938 | 0.454466 | 0.895431 | 0.0318024 | 1.01E-07 | 4.79E-03 | 2.84E+01 | 3.10E-04 |
| YgJTec | TIBC | b7vI1p | NSCLC | rs8177257 | T | C | T | C | -0.2878 | -0.0109817 | 0.0735 | 0.0661493 | 0.864579 | 0.0643904 | 1.39E-169 | 1.04E-02 | 7.71E+02 | 1.13E-02 |
| YgJTec | TIBC | b7vI1p | NSCLC | rs854762 | G | A | G | A | 0.0216 | 0.0243389 | 0.3862 | 0.486335 | 0.444799 | 0.0318524 | 5.38E-06 | 4.75E-03 | 2.07E+01 | 2.21E-04 |
| YgJTec | TIBC | b7vI1p | NSCLC | rs855791 | A | G | A | G | 0.026 | 0.0558405 | 0.406 | 0.346039 | 0.0906964 | 0.0330078 | 2.88E-08 | 4.69E-03 | 3.08E+01 | 3.26E-04 |
| YgJTec | TIBC | b7vI1p | NSCLC | rs9261848 | C | G | C | G | 0.0297 | -0.0404923 | 0.1582 | 0.0858854 | 0.470858 | 0.0561548 | 5.63E-06 | 6.54E-03 | 2.06E+01 | 2.35E-04 |
| YgJTec | TIBC | b7vI1p | NSCLC | rs9389269 | C | T | C | T | -0.0336 | -0.0410532 | 0.2792 | 0.342476 | 0.219182 | 0.0334117 | 9.10E-11 | 5.18E-03 | 4.20E+01 | 4.54E-04 |
| YgJTec | TIBC | b7vI1p | NSCLC | rs968155 | C | T | C | T | -0.0547 | 0.0682801 | 0.4638 | 0.440075 | 0.0314109 | 0.0317312 | 3.45E-31 | 4.71E-03 | 1.35E+02 | 1.49E-03 |

| **Supplementary Table 9 Statistical characteristics of eligible instrumental variables in forward MR analysis.** | | | | | | | | | | | | | | | | | | |
| --- | --- | --- | --- | --- | --- | --- | --- | --- | --- | --- | --- | --- | --- | --- | --- | --- | --- | --- |
| **TSAT AND NSCLC** | | | | | | | | | | | | | | | | | | |
| **id.exposure** | **exposure** | **id.outcome** | **outcome** | **SNP** | **effect_allele.exposure** | **other_allele.exposure** | **effect_allele.outcome** | **other_allele.outcome** | **beta.exposure** | **beta.outcome** | **eaf.exposure** | **eaf.outcome** | **pval.outcome** | **se.outcome** | **pval.exposure** | **se.exposure** | **F** | **R2** |
| 25xisD | TSAT | k9YkBa | NSCLC | rs10006312 | C | G | C | G | -0.0341 | 0.0197607 | 0.0855 | 0.123898 | 0.690349 | 0.0496025 | 5.72E-06 | 0.007516836 | 20.57967954 | 0.00018184 |
| 25xisD | TSAT | k9YkBa | NSCLC | rs10421599 | A | G | A | G | -0.03 | -0.00164346 | 0.2051 | 0.219 | 0.965776 | 0.0383034 | 4.95E-08 | 0.005501461 | 29.73626595 | 0.000293461 |
| 25xisD | TSAT | k9YkBa | NSCLC | rs10754329 | G | C | G | C | 0.0224 | 0.0112913 | 0.2984 | 0.301096 | 0.741589 | 0.0342421 | 5.59E-06 | 0.004932474 | 20.62369262 | 0.000210094 |
| 25xisD | TSAT | k9YkBa | NSCLC | rs10808546 | T | C | T | C | -0.0227 | 0.035338 | 0.4779 | 0.430609 | 0.267009 | 0.0318368 | 5.89E-07 | 0.004544736 | 24.94792291 | 0.000257142 |
| 25xisD | TSAT | k9YkBa | NSCLC | rs10815156 | A | G | A | G | -0.0217 | 0.0358904 | 0.4959 | 0.617135 | 0.27734 | 0.0330387 | 5.92E-06 | 0.004791105 | 20.5138932 | 0.000235429 |
| 25xisD | TSAT | k9YkBa | NSCLC | rs10822143 | C | T | C | T | -0.0227 | -0.0425585 | 0.4548 | 0.472659 | 0.179551 | 0.0317095 | 5.80E-07 | 0.004542035 | 24.97760317 | 0.000255539 |
| 25xisD | TSAT | k9YkBa | NSCLC | rs114708114 | T | C | T | C | 0.1608 | -0.0510408 | 0.0282 | 0.00399419 | 0.845095 | 0.261238 | 4.03E-32 | 0.013630229 | 139.1764594 | 0.00141719 |
| 25xisD | TSAT | k9YkBa | NSCLC | rs115312664 | A | G | A | G | 0.0772 | -0.062085 | 0.0169 | 0.0125938 | 0.659859 | 0.141068 | 2.58E-06 | 0.016419581 | 22.10601497 | 0.000198038 |
| 25xisD | TSAT | k9YkBa | NSCLC | rs115314215 | T | C | T | C | -0.0559 | -0.0490369 | 0.0653 | 0.0329492 | 0.573687 | 0.0871565 | 1.20E-08 | 0.009807489 | 32.48687642 | 0.000381451 |
| 25xisD | TSAT | k9YkBa | NSCLC | rs11576708 | A | G | A | G | -0.03 | -0.0145128 | 0.1421 | 0.0747087 | 0.807332 | 0.0595107 | 4.85E-06 | 0.006562866 | 20.89562836 | 0.000219434 |
| 25xisD | TSAT | k9YkBa | NSCLC | rs116272812 | C | T | C | T | 0.2459 | -0.0028075 | 0.1181 | 0.128953 | 0.952391 | 0.0470233 | 1.00E-200 | 0.006857077 | 1285.994185 | 0.012595526 |
| 25xisD | TSAT | k9YkBa | NSCLC | rs116493005 | G | A | G | A | 0.0842 | -0.0388706 | 0.0139 | 0.0247919 | 0.713648 | 0.105926 | 8.18E-06 | 0.018877155 | 19.89532781 | 0.000194352 |
| 25xisD | TSAT | k9YkBa | NSCLC | rs117718169 | T | C | T | C | -0.0694 | 0.199773 | 0.0326 | 0.0190481 | 0.0809189 | 0.114459 | 9.82E-09 | 0.012103647 | 32.87656555 | 0.000303789 |
| 25xisD | TSAT | k9YkBa | NSCLC | rs1223763 | T | G | T | G | 0.0272 | 0.0511174 | 0.2304 | 0.19907 | 0.195363 | 0.0394767 | 1.29E-06 | 0.005618339 | 23.43807436 | 0.000262371 |
| 25xisD | TSAT | k9YkBa | NSCLC | rs12478088 | G | A | G | A | 0.0263 | -0.0123036 | 0.3885 | 0.428951 | 0.700932 | 0.0320353 | 1.75E-08 | 0.004667211 | 31.75386735 | 0.000328646 |
| 25xisD | TSAT | k9YkBa | NSCLC | rs12633819 | G | A | G | A | 0.0285 | -0.0251154 | 0.3879 | 0.276921 | 0.473583 | 0.0350449 | 1.66E-09 | 0.004727935 | 36.33680394 | 0.000385711 |
| 25xisD | TSAT | k9YkBa | NSCLC | rs12975762 | G | A | G | A | -0.032 | -0.00724696 | 0.328 | 0.232222 | 0.846469 | 0.0374277 | 5.83E-11 | 0.004886964 | 42.87673204 | 0.000451412 |
| 25xisD | TSAT | k9YkBa | NSCLC | rs12976652 | C | T | C | T | 0.0325 | -0.0826726 | 0.0979 | 0.06336 | 0.191923 | 0.063355 | 8.92E-06 | 0.007316816 | 19.72978963 | 0.000186567 |
| 25xisD | TSAT | k9YkBa | NSCLC | rs13007705 | T | C | T | C | 0.0331 | 0.0038828 | 0.3745 | 0.38328 | 0.904682 | 0.0324245 | 1.10E-12 | 0.004650589 | 50.6570701 | 0.000513293 |
| 25xisD | TSAT | k9YkBa | NSCLC | rs13112934 | T | G | T | G | 0.0229 | 0.0588827 | 0.4649 | 0.435057 | 0.0656311 | 0.0319853 | 9.39E-07 | 0.004669648 | 24.04931305 | 0.000260913 |
| 25xisD | TSAT | k9YkBa | NSCLC | rs144063236 | T | C | T | C | 0.1137 | 0.0668698 | 0.006 | 0.042338 | 0.403083 | 0.0799755 | 5.20E-06 | 0.024953075 | 20.76217205 | 0.000154201 |
| 25xisD | TSAT | k9YkBa | NSCLC | rs149830227 | T | C | T | C | 0.0649 | -0.0949425 | 0.028 | 0.00839348 | 0.589624 | 0.176021 | 7.90E-06 | 0.014525917 | 19.96191845 | 0.000229268 |
| 25xisD | TSAT | k9YkBa | NSCLC | rs174560 | C | T | C | T | -0.022 | -0.0877495 | 0.3531 | 0.388931 | 0.00657976 | 0.0322917 | 4.51E-06 | 0.004796813 | 21.03486772 | 0.000221111 |
| 25xisD | TSAT | k9YkBa | NSCLC | rs188601727 | A | G | A | G | -0.108 | -0.112688 | 0.0102 | 0.0272948 | 0.262399 | 0.100548 | 6.37E-06 | 0.023927044 | 20.3736769 | 0.000235519 |
| 25xisD | TSAT | k9YkBa | NSCLC | rs1958078 | A | C | A | C | -0.0336 | -0.0425542 | 0.133 | 0.080573 | 0.464802 | 0.0582166 | 2.05E-07 | 0.006468074 | 26.98538353 | 0.000260363 |
| 25xisD | TSAT | k9YkBa | NSCLC | rs218264 | T | A | T | A | 0.0263 | -0.0789741 | 0.2509 | 0.250444 | 0.0317154 | 0.0367667 | 4.62E-07 | 0.005216746 | 25.41628745 | 0.000260005 |
| 25xisD | TSAT | k9YkBa | NSCLC | rs2510072 | A | G | A | G | 0.0349 | -0.0305268 | 0.1217 | 0.111189 | 0.543029 | 0.0501889 | 7.58E-07 | 0.007056376 | 24.46174449 | 0.000260384 |
| 25xisD | TSAT | k9YkBa | NSCLC | rs28487964 | A | T | A | T | -0.0692 | 0.139473 | 0.037 | 0.0156678 | 0.277871 | 0.128533 | 3.05E-08 | 0.012494263 | 30.67544714 | 0.000341248 |
| 25xisD | TSAT | k9YkBa | NSCLC | rs2900478 | A | T | A | T | -0.0283 | 0.0375631 | 0.1754 | 0.218506 | 0.328092 | 0.0384095 | 2.62E-06 | 0.00602312 | 22.07648057 | 0.000231673 |
| 25xisD | TSAT | k9YkBa | NSCLC | rs4133213 | A | C | A | C | 0.0245 | -0.0128981 | 0.4436 | 0.311617 | 0.704883 | 0.0340557 | 1.01E-07 | 0.004601012 | 28.35472482 | 0.000296306 |
| 25xisD | TSAT | k9YkBa | NSCLC | rs4712972 | A | G | A | G | -0.1016 | 0.0196022 | 0.0896 | 0.085775 | 0.723203 | 0.0553451 | 4.57E-46 | 0.007130498 | 203.0241454 | 0.00168406 |
| 25xisD | TSAT | k9YkBa | NSCLC | rs4774514 | T | C | T | C | -0.069 | -0.0993066 | 0.0665 | 0.039001 | 0.231952 | 0.0830778 | 3.17E-13 | 0.009468954 | 53.09995682 | 0.000591104 |
| 25xisD | TSAT | k9YkBa | NSCLC | rs4817984 | A | C | A | C | 0.0228 | 0.0339361 | 0.3116 | 0.260346 | 0.344123 | 0.0358714 | 4.57E-06 | 0.004974237 | 21.00954982 | 0.000223017 |
| 25xisD | TSAT | k9YkBa | NSCLC | rs55980600 | C | G | C | G | 0.0414 | -0.0191673 | 0.0738 | 0.0564489 | 0.778033 | 0.067997 | 1.40E-06 | 0.008580303 | 23.28066777 | 0.000234311 |
| 25xisD | TSAT | k9YkBa | NSCLC | rs565454423 | T | C | T | C | 0.0754 | 0.121253 | 0.0212 | 0.0223845 | 0.269881 | 0.109896 | 2.15E-06 | 0.015911241 | 22.45611295 | 0.000235941 |
| 25xisD | TSAT | k9YkBa | NSCLC | rs56912861 | A | G | A | G | -0.038 | -0.0344828 | 0.3658 | 0.470138 | 0.275925 | 0.0316495 | 1.16E-15 | 0.004744886 | 64.13803172 | 0.000669988 |
| 25xisD | TSAT | k9YkBa | NSCLC | rs57933581 | A | T | A | T | 0.1094 | -0.293683 | 0.0098 | 0.0012664 | 0.493791 | 0.429177 | 4.20E-06 | 0.023776243 | 21.171332 | 0.000232281 |
| 25xisD | TSAT | k9YkBa | NSCLC | rs6025 | T | C | T | C | 0.0841 | 0.250939 | 0.0188 | 0.0202383 | 0.0271957 | 0.113614 | 1.71E-07 | 0.016085282 | 27.33597907 | 0.000260938 |
| 25xisD | TSAT | k9YkBa | NSCLC | rs6077462 | C | T | C | T | -0.0233 | 0.0394207 | 0.2452 | 0.217247 | 0.30189 | 0.038184 | 7.36E-06 | 0.005197409 | 20.09731566 | 0.000200953 |
| 25xisD | TSAT | k9YkBa | NSCLC | rs62258925 | T | G | T | G | -0.0274 | -0.0488164 | 0.1895 | 0.155358 | 0.259177 | 0.0432639 | 2.19E-06 | 0.005786632 | 22.42071184 | 0.000230618 |
| 25xisD | TSAT | k9YkBa | NSCLC | rs62396224 | G | A | G | A | -0.0597 | 0.0515404 | 0.0507 | 0.0671286 | 0.420316 | 0.0639559 | 7.81E-09 | 0.010342124 | 33.32185584 | 0.000343076 |
| 25xisD | TSAT | k9YkBa | NSCLC | rs6592965 | A | G | A | G | 0.0262 | 0.0246161 | 0.4005 | 0.32058 | 0.467501 | 0.0338808 | 1.09E-08 | 0.004583549 | 32.67373825 | 0.000329628 |
| 25xisD | TSAT | k9YkBa | NSCLC | rs72838865 | A | C | A | C | 0.1029 | 0.181118 | 0.0339 | 0.0178777 | 0.128661 | 0.119203 | 3.19E-15 | 0.013053009 | 62.14546967 | 0.000693558 |
| 25xisD | TSAT | k9YkBa | NSCLC | rs72978402 | C | T | C | T | -0.0334 | -0.0223307 | 0.0867 | 0.115159 | 0.651582 | 0.0494517 | 9.60E-06 | 0.007546331 | 19.58940444 | 0.000176667 |
| 25xisD | TSAT | k9YkBa | NSCLC | rs73060317 | G | T | G | T | -0.0235 | -0.0536109 | 0.2805 | 0.309596 | 0.117999 | 0.034295 | 2.99E-06 | 0.005030498 | 21.82296535 | 0.00022291 |
| 25xisD | TSAT | k9YkBa | NSCLC | rs7385804 | C | A | C | A | -0.0618 | 0.00535565 | 0.3516 | 0.448509 | 0.866057 | 0.0317524 | 2.87E-39 | 0.004713807 | 171.8831706 | 0.001741401 |
| 25xisD | TSAT | k9YkBa | NSCLC | rs74338506 | A | G | A | G | -0.085 | -0.0887089 | 0.0201 | 0.0223642 | 0.421646 | 0.110394 | 2.87E-08 | 0.015317557 | 30.79348493 | 0.000284607 |
| 25xisD | TSAT | k9YkBa | NSCLC | rs7514450 | T | C | T | C | -0.0224 | 0.0269991 | 0.4507 | 0.452916 | 0.395531 | 0.0317775 | 1.03E-06 | 0.004584697 | 23.87122791 | 0.000248441 |
| 25xisD | TSAT | k9YkBa | NSCLC | rs75965181 | A | T | A | T | -0.0691 | -0.185978 | 0.0283 | 0.0122635 | 0.189495 | 0.141744 | 2.47E-06 | 0.014669074 | 22.18966246 | 0.000262606 |
| 25xisD | TSAT | k9YkBa | NSCLC | rs7613393 | C | A | C | A | -0.0472 | -0.0575951 | 0.0411 | 0.0809951 | 0.324668 | 0.0584775 | 9.93E-06 | 0.010681894 | 19.52483935 | 0.000175602 |
| 25xisD | TSAT | k9YkBa | NSCLC | rs7648210 | A | G | A | G | 0.0371 | 0.0421019 | 0.2444 | 0.245102 | 0.250649 | 0.0366494 | 2.53E-12 | 0.005298766 | 49.02282533 | 0.00050836 |
| 25xisD | TSAT | k9YkBa | NSCLC | rs7837764 | C | G | C | G | 0.0261 | 0.0333165 | 0.4021 | 0.378465 | 0.304722 | 0.0324609 | 1.61E-08 | 0.004619952 | 31.91582594 | 0.000327547 |
| 25xisD | TSAT | k9YkBa | NSCLC | rs79385984 | A | G | A | G | 0.0844 | 0.0669728 | 0.0173 | 0.0094175 | 0.67959 | 0.162153 | 8.56E-06 | 0.018963407 | 19.80852605 | 0.000242204 |
| 25xisD | TSAT | k9YkBa | NSCLC | rs8176044 | C | T | C | T | 0.0231 | -0.0210886 | 0.3204 | 0.315697 | 0.535038 | 0.0339957 | 1.63E-06 | 0.00481792 | 22.98819069 | 0.000232381 |
| 25xisD | TSAT | k9YkBa | NSCLC | rs8177271 | A | G | A | G | -0.0977 | 0.00839919 | 0.2701 | 0.305103 | 0.805123 | 0.034043 | 5.03E-87 | 0.004940942 | 390.9935703 | 0.003763631 |
| 25xisD | TSAT | k9YkBa | NSCLC | rs9399136 | C | T | C | T | 0.0673 | -0.0473938 | 0.2695 | 0.343836 | 0.157217 | 0.0335058 | 5.32E-39 | 0.005151746 | 170.6558748 | 0.00178336 |
| 25xisD | TSAT | k9YkBa | NSCLC | rs9610638 | T | C | T | C | -0.1561 | 0.00127914 | 0.3972 | 0.401799 | 0.968253 | 0.0321395 | 1.00E-200 | 0.004609473 | 1146.841118 | 0.011668587 |
| 25xisD | TSAT | k9YkBa | NSCLC | rs997355 | T | A | T | A | -0.0275 | -0.0327306 | 0.1919 | 0.301617 | 0.344967 | 0.0346577 | 2.37E-06 | 0.005827499 | 22.26901318 | 0.00023455 |

| **Supplementary Table 10** | | | | | | |
| --- | --- | --- | --- | --- | --- | --- |
| **Reverse MR Analysis of FERRITIN and SCLC** | | | | | | |
| **Exposure** | **Outcome** | **Method** | **nSNP** | **β** | **95%CI** | **P-val** |
| SCLC | FERRITIN | MR Egger | 22 | -0.006 | 0.981,1.007 | 0.399 |
|  |  | Weighted median | 22 | -0.002 | 0.990,1.006 | 0.689 |
|  |  | Inverse variance weighted | 22 | -0.001 | 0.993,1.005 | 0.68 |
|  |  | Simple mode | 22 | -0.001 | 0.983,1.005 | 0.875 |
|  |  | Weighted mode | 22 | -0.006 | 0.981,1.008 | 0.419 |
| nSNP=number of Single Nucleotide Polymorphisms,SCLC=small cell lung cancer, NSCLC=non-small cell lung cancer. | | | | | | |

| **Supplementary Table 11** | | | | | |
| --- | --- | --- | --- | --- | --- |
| **Sensitivity analyses for the reverse MR of SCLC on FERRITIN** | | | | | |
| **Exposure** | **Outcome** | **Heterogeneity test** | | **Pleiotropy test** | **MR-PRESSO** |
| SCLC | FERRITIN | **Cochran’s Q test(P-value) Inverse variance weighted** | **Rucker’s Q test (P-value) MR Egger** | **Egger intercept (P-value) MR Egger** | **Global Test (P-value)** |
| SCLC | FERRITIN | 0.558 | 0.521 | 0.547 | 0.58 |
| SCLC=small cell lung cancer | | | | | |

| **Supplementary Table 12** | | | |
| --- | --- | --- | --- |
| **Summary of Benjamini –Hochberg FDR-adjusted p-values** | | | |
| **Exposure** | **Outcome** | **p (raw)** | **p (FDR-adj)** |
| FERRITIN | SCLC | 0.045 | 0.18 |
| SERUM | SCLC | 0.657 | 0.801 |
| TIBC | SCLC | 0.801 | 0.801 |
| TIBC | SCLC | 0.801 | 0.801 |
| FERRITIN | NSCLC | 0.127 | 0.507 |
| SERUM | NSCLC | 0.938 | 0.938 |
| TIBC | NSCLC | 0.189 | 0.507 |
| TIBC | NSCLC | 0.923 | 0.938 |
