# Supplementary material for: Investigating the genetic causal link between iron regulation and lung cancer risk: A 2-sample Mendelian randomization analysis
Source: Medicine (Baltimore). 2025 Oct 24;104(43):e45518. doi: 10.1097/MD.0000000000045518 (PMC12558328; doi:10.1097/MD.0000000000045518)

**FigureS1.**Leave-one-out analysis of the association between ferritin and small cell lung cancer risk. Each black dot represents the result of the IVW method after excluding a specific SNP, and the red dot represents the IVW estimate for all SNPs.


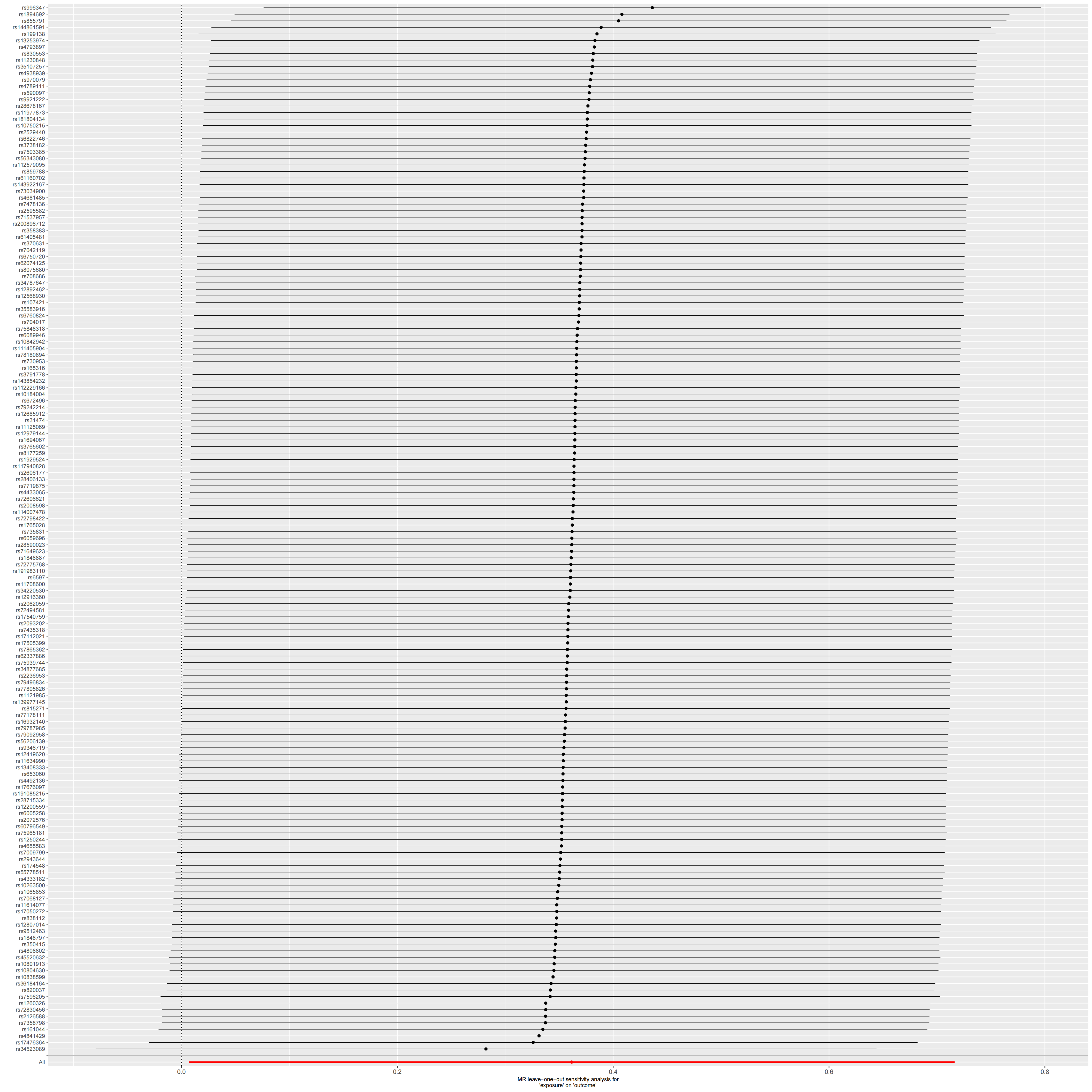


**FigureS2.**Leave-one-out analysis of the association between serum iron and small cell lung cancer risk. Each black dot represents the result of the IVW method after excluding a specific SNP, and the red dot represents the IVW estimate for all SNPs.


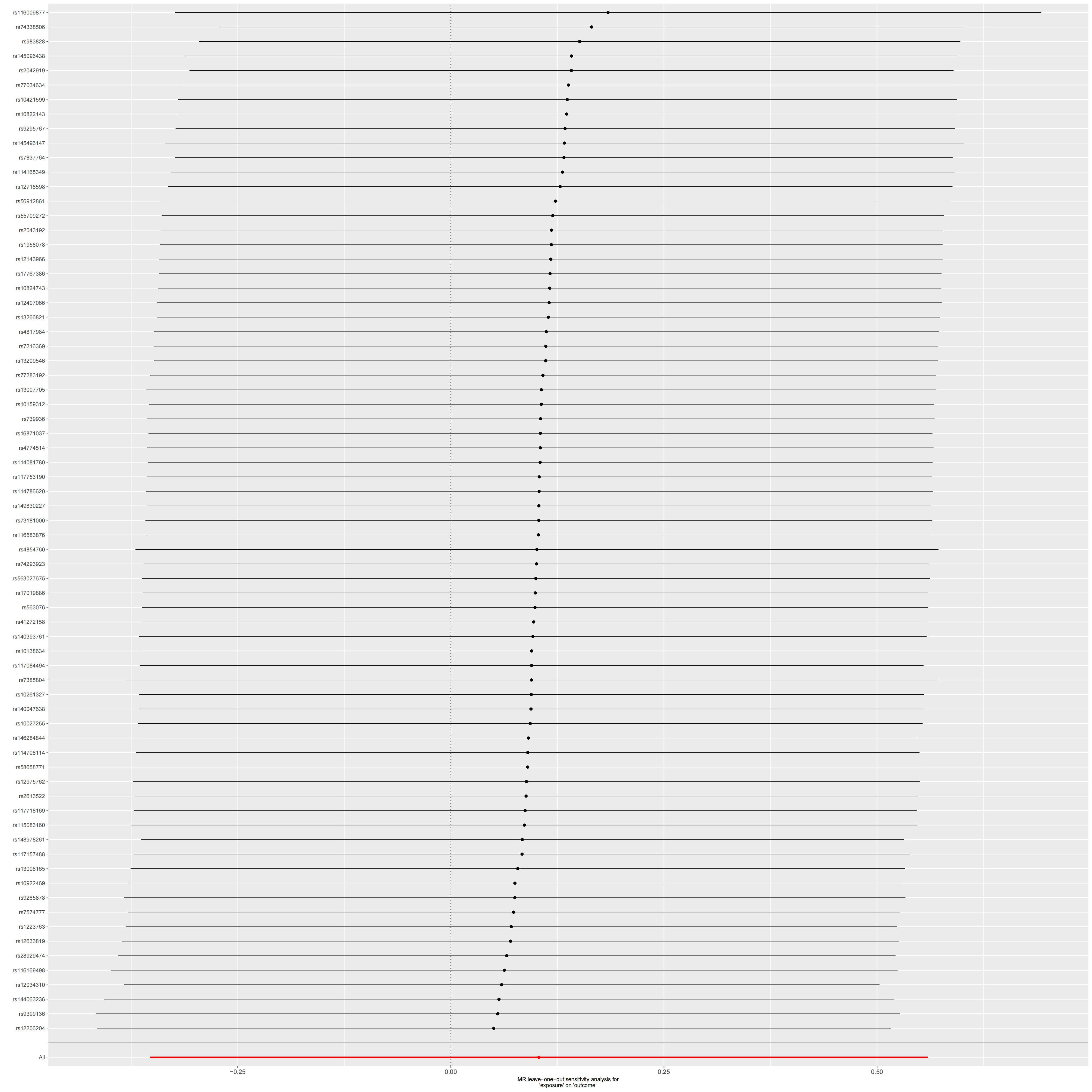


**FigureS3.**Leave-one-out analysis of the association between TIBC (Total Iron-Binding Capacity) and small cell lung cancer risk. Each black dot represents the result of the IVW method after excluding a specific SNP, and the red dot represents the IVW estimate for all SNPs.


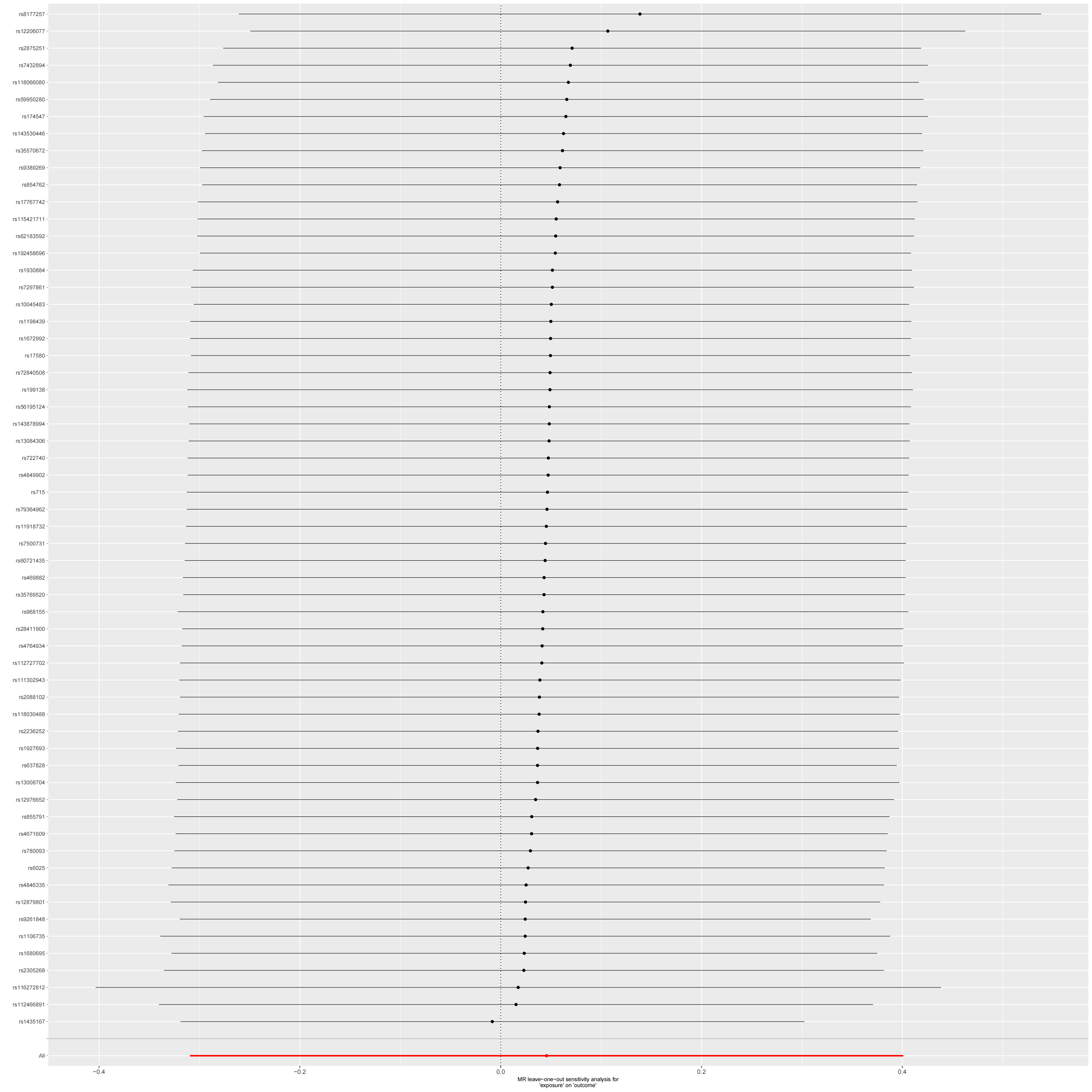


**FigureS4.**Leave-one-out analysis of the association between TSAT (Transferrin Saturation) and small cell lung cancer risk. Each black dot represents the result of the IVW method after excluding a specific SNP, and the red dot represents the IVW estimate for all SNPs.


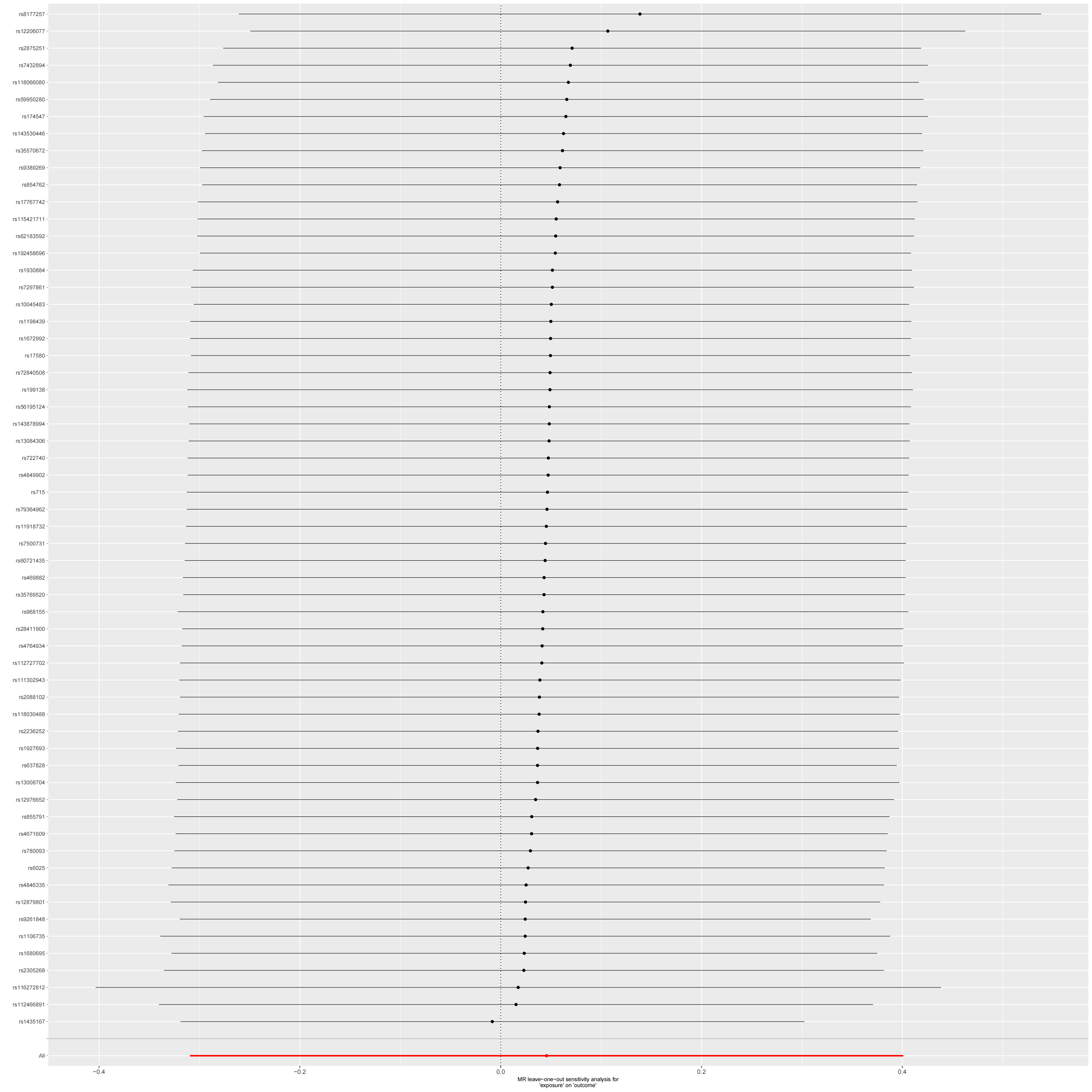


**FigureS5.**Leave-one-out analysis of the association between ferritin and non-small cell lung cancer risk. Each black dot represents the result of the IVW method after excluding a specific SNP, and the red dot represents the IVW estimate for all SNPs.


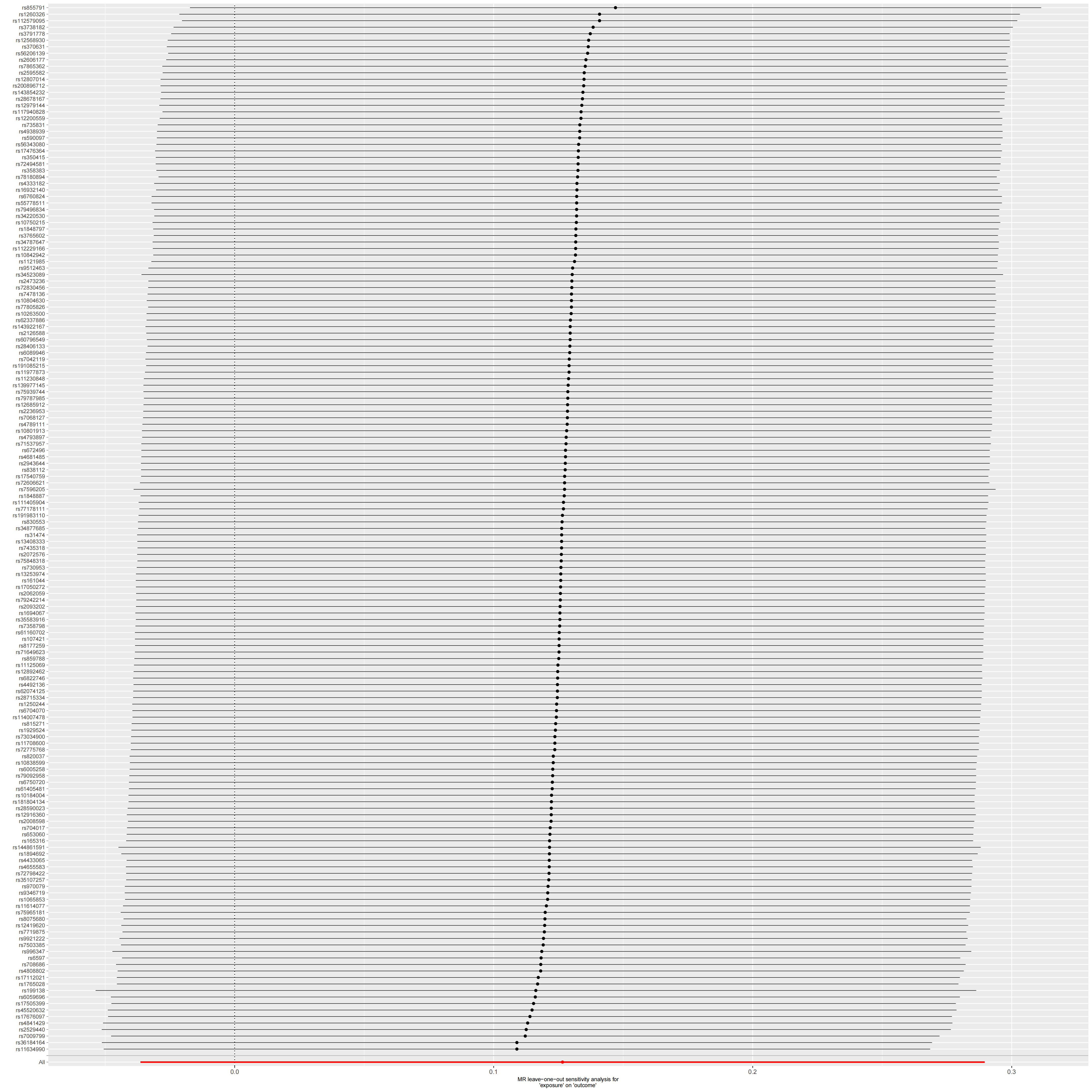


**FigureS6.**Leave-one-out analysis of the association between serum iron and non-small cell lung cancer risk. Each black dot represents the result of the IVW method after excluding a specific SNP, and the red dot represents the IVW estimate for all SNPs.


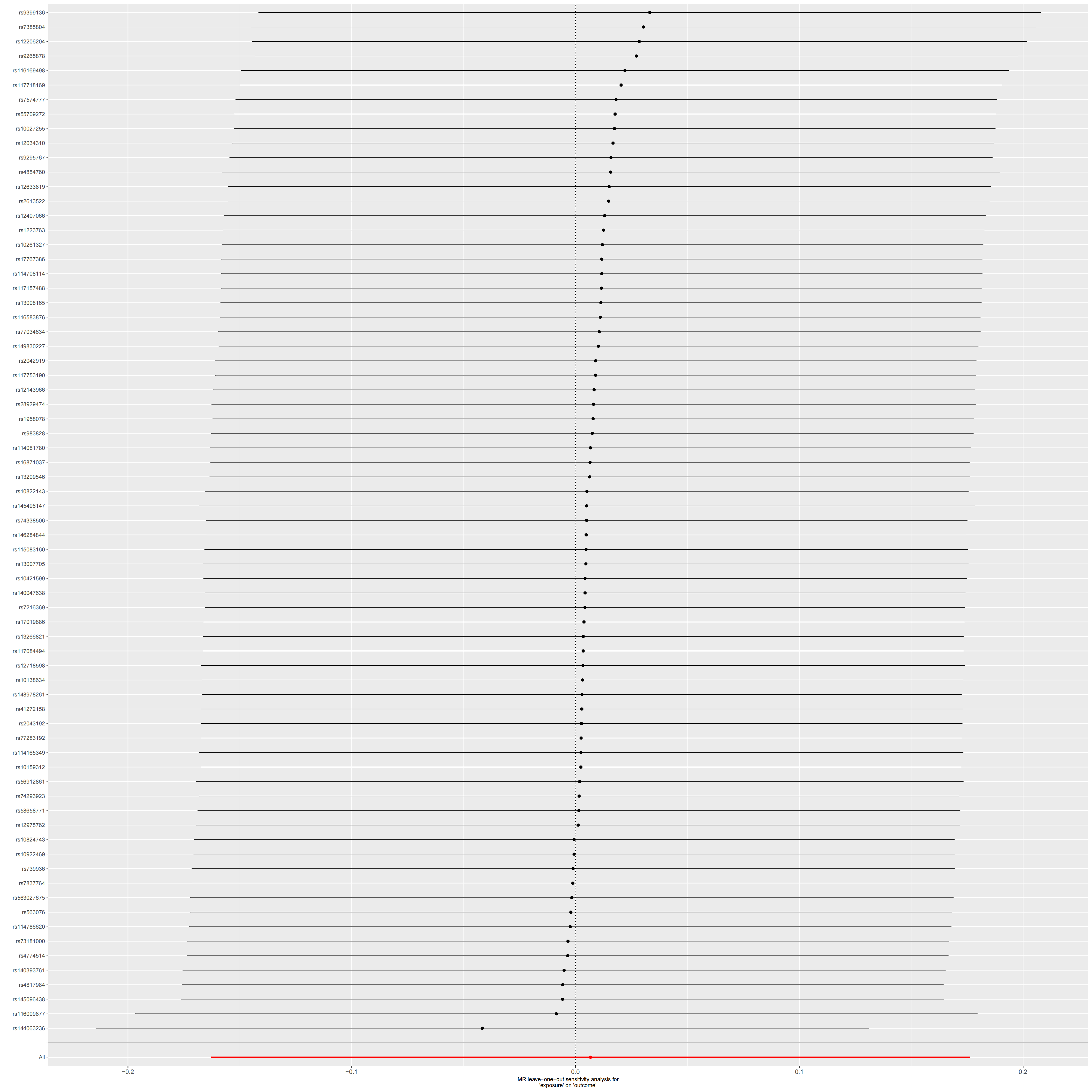


**FigureS7.**Leave-one-out analysis of the association between TIBC (Total Iron Binding Capacity) and non-small cell lung cancer risk. Each black dot represents the result of the IVW method after excluding a specific SNP, and the red dot represents the IVW estimate for all SNPs.


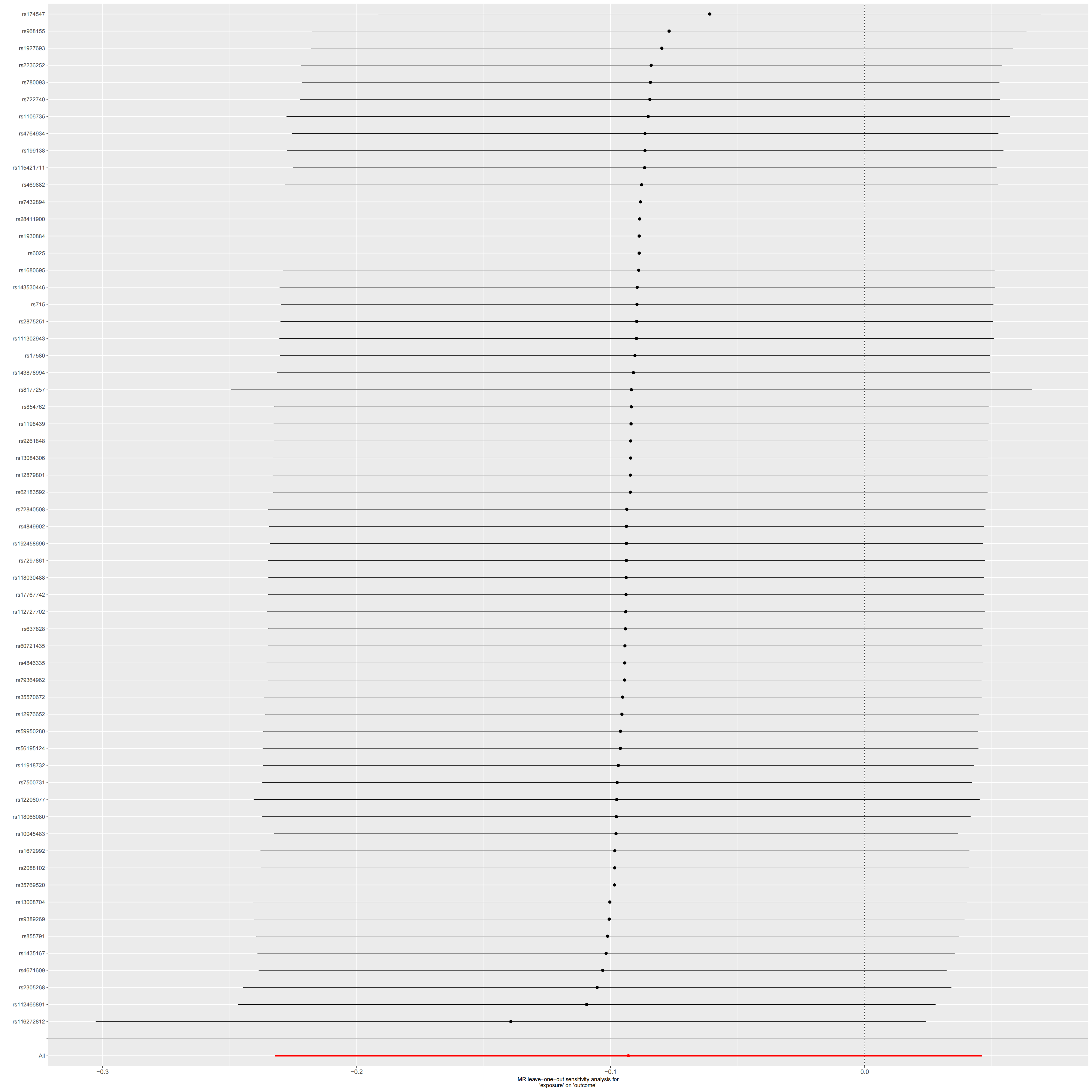


**FigureS8.**Leave-one-out analysis of the association between TSAT (Transferrin Saturation) and non-small cell lung cancer risk. Each black dot represents the result of the IVW method after excluding a specific SNP, and the red dot represents the IVW estimate for all SNPs.


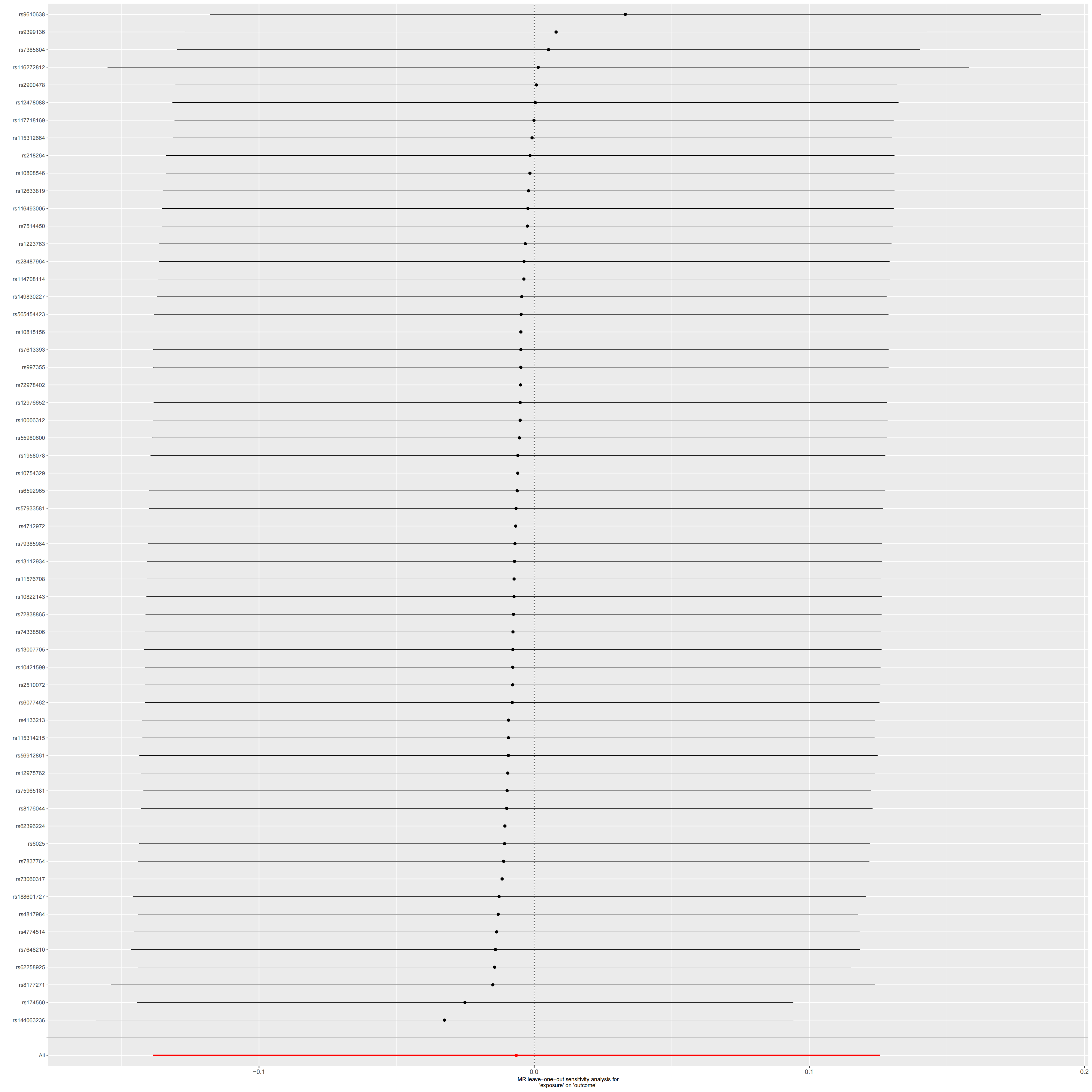

Supplement: Supplementary file 2 [file medi-104-e45518-s002.docx]
